# Supplementary material for: Genetically modified macrophages accelerate myelin repair
Source: EMBO Mol Med. 2022 Jul 13;14(8):e14759. doi: 10.15252/emmm.202114759 (PMC9358396; doi:10.15252/emmm.202114759)
Supplement: Supplementary file 1 — Appendix [file EMMM-14-e14759-s010.pdf]

APPENDIX-SUPPLEMENTARY DATA

Table of content:

Appendix Figure S1 and legend: Scheme illustrating lentiviral vector constructions used in the study

Appendix Figure S2 and legend: Viability and proliferation/differentiation following Sema3F transduction in vitro

Appendix Figure S3 and legend: Oil Red O staining in chimeric mouse spinal cord sections at 7 days post lesion

Appendix Figure S4 and legend: Morphology of GFP+ cells in the spinal cord

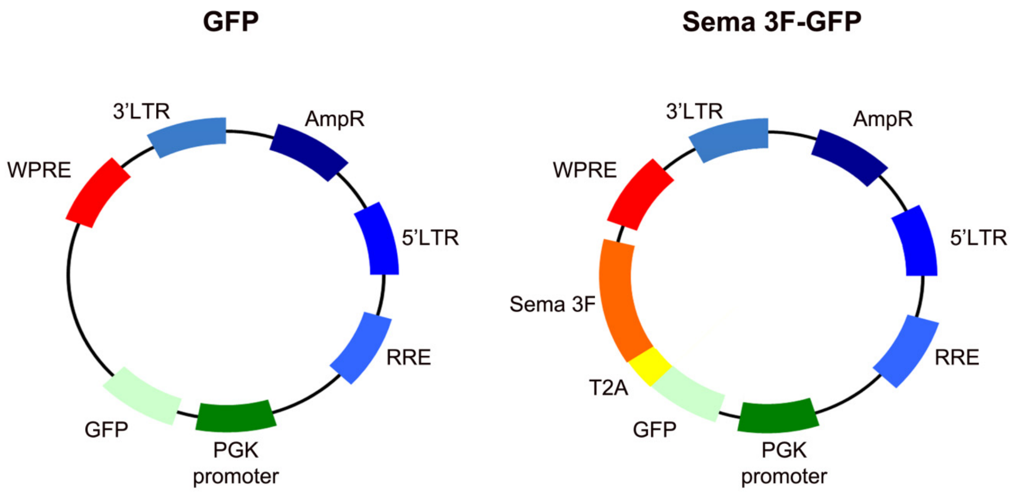

Appendix Figure S1. Scheme illustrating lentiviral vector constructions used in the study. WPRE-Woodchuck hepatitis virus Posttranscriptional Regulatory Element; LTR-Long Terminal Repeat; AmpR-Ampicillin Resistance; RRE- Rev Response Element; PGK-PhosphoGlycerate Kinase; GFP-Green Fluorescent protein; Sema-Semaphorin.

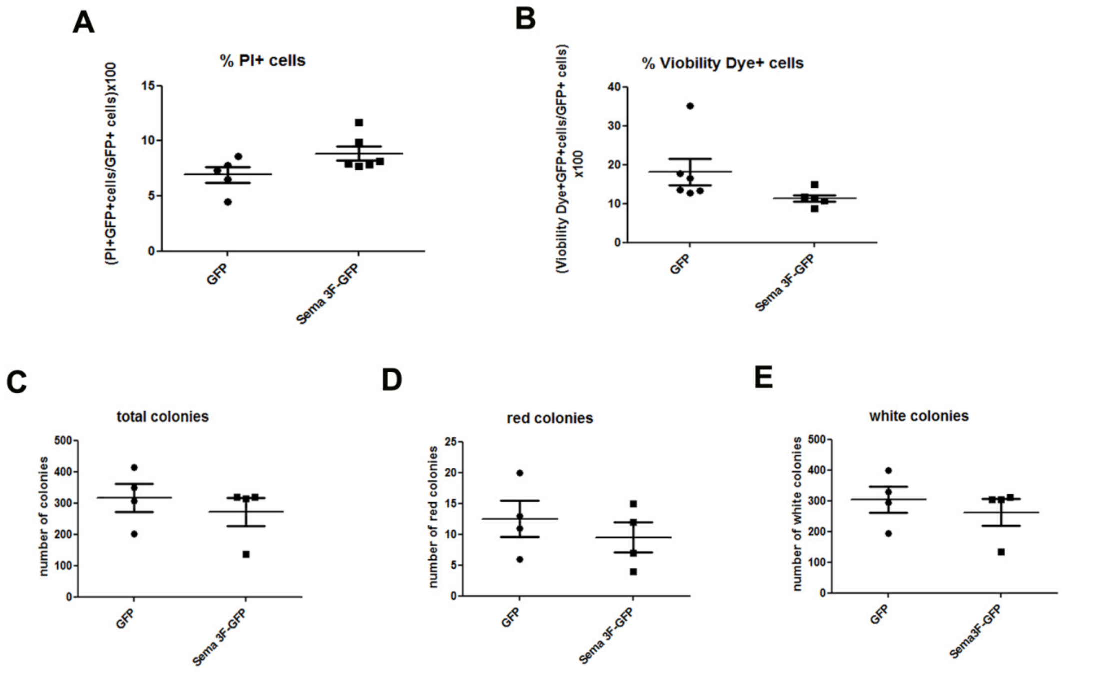

Appendix Fig S2. Viability and proliferation/differentiation following Sema3F transduction in vitro. A-B. Flow cytometry analyses of cell death. Hematopoietic stem/progenitor cell preparation was transduced in vitro using PGK-GFP and PGK-Sema3F-GFP lentiviral vectors and expanded for 5 days. Dead/apoptotic cells were labeled using Propidium Iodide (PI) and Viobility 405/520 Fixable Dye. n= 5-6 independent experiments. **A**. PI labelling. **B**. Viobility Dye labelling. **C-E**. Colony assay. n = 2 replicates from each of 2 independent experiments. C. Total colonies formed by transduced cell preparations. D. Red colonies formed by transduced cell preparations. E. White colonies formed by transduced cell preparations.

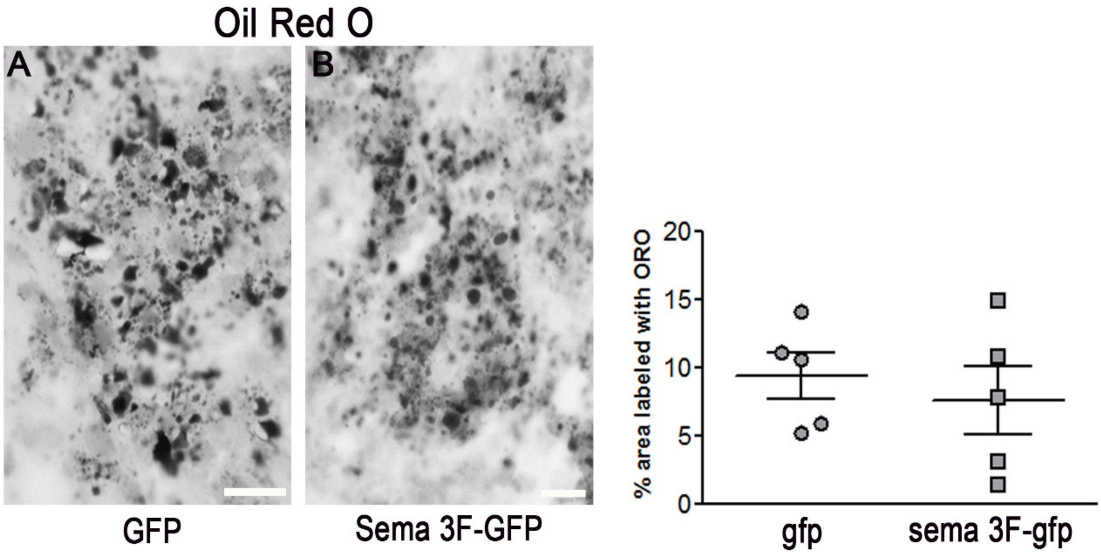

Appendix Figure S3. Oil Red O staining in chimeric mouse spinal cord sections at 7 days post lesion. A. GFP mouse. **B**. Sema3F-GFP mouse. C. The extent of labelling is similar between the two groups. n = 5 mice/group. Scale bars = 50 µm.

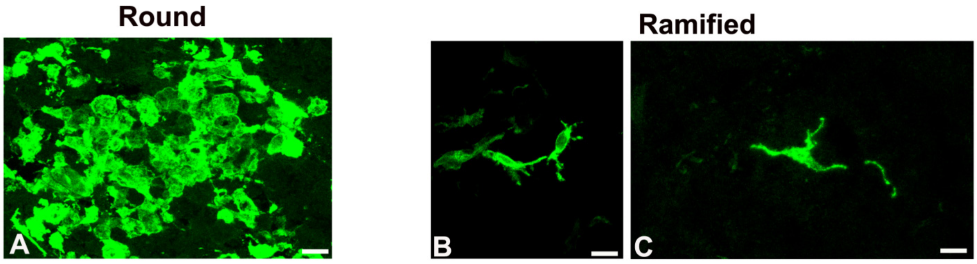

Appendix Figure S4. Morphology of GFP+ cells in the spinal cord. A. Large, round cells in a lesion at 7 dpl. **B-C**. Ramified cells observed in the lesion-neighbouring tissue at 7 and 10 dpl, but also in the lesions at 60 dpl. Scale bars A=20 µm, B=10 µm, and C=10 µm.
