# Supplementary figures and images for: Genetically modified macrophages accelerate myelin repair
Source: EMBO Mol Med. 2022 Jul 13;14(8):e14759. doi: 10.15252/emmm.202114759 (PMC9358396; doi:10.15252/emmm.202114759)

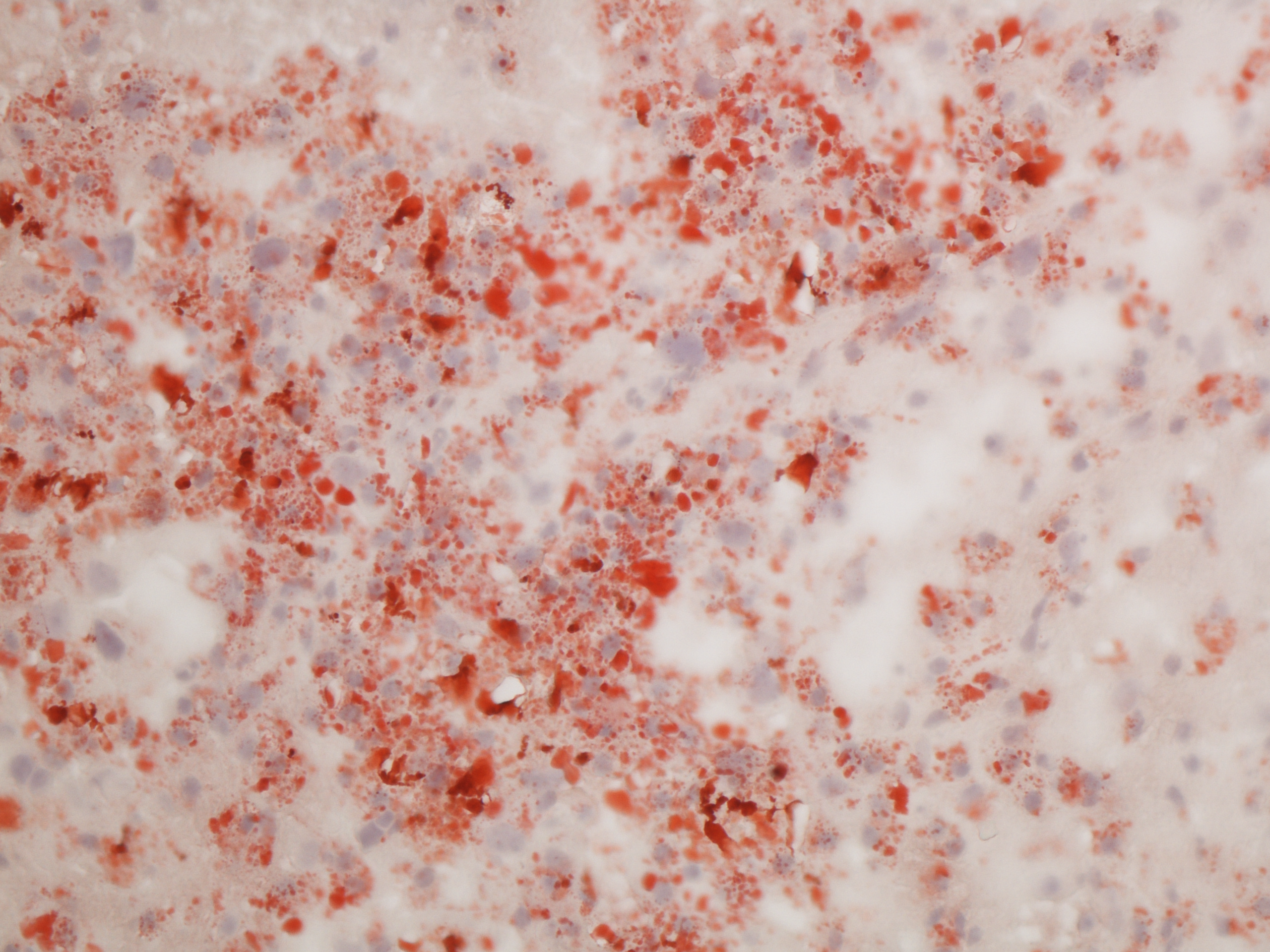

Supplement: Supplementary file 2 — Source Data for Appendix [file EMMM-14-e14759-s004.zip › Appendix Supplementary Data/Appendix Supplementary Figure 3/Oil Red O images/907A_15122021_40X.tif]

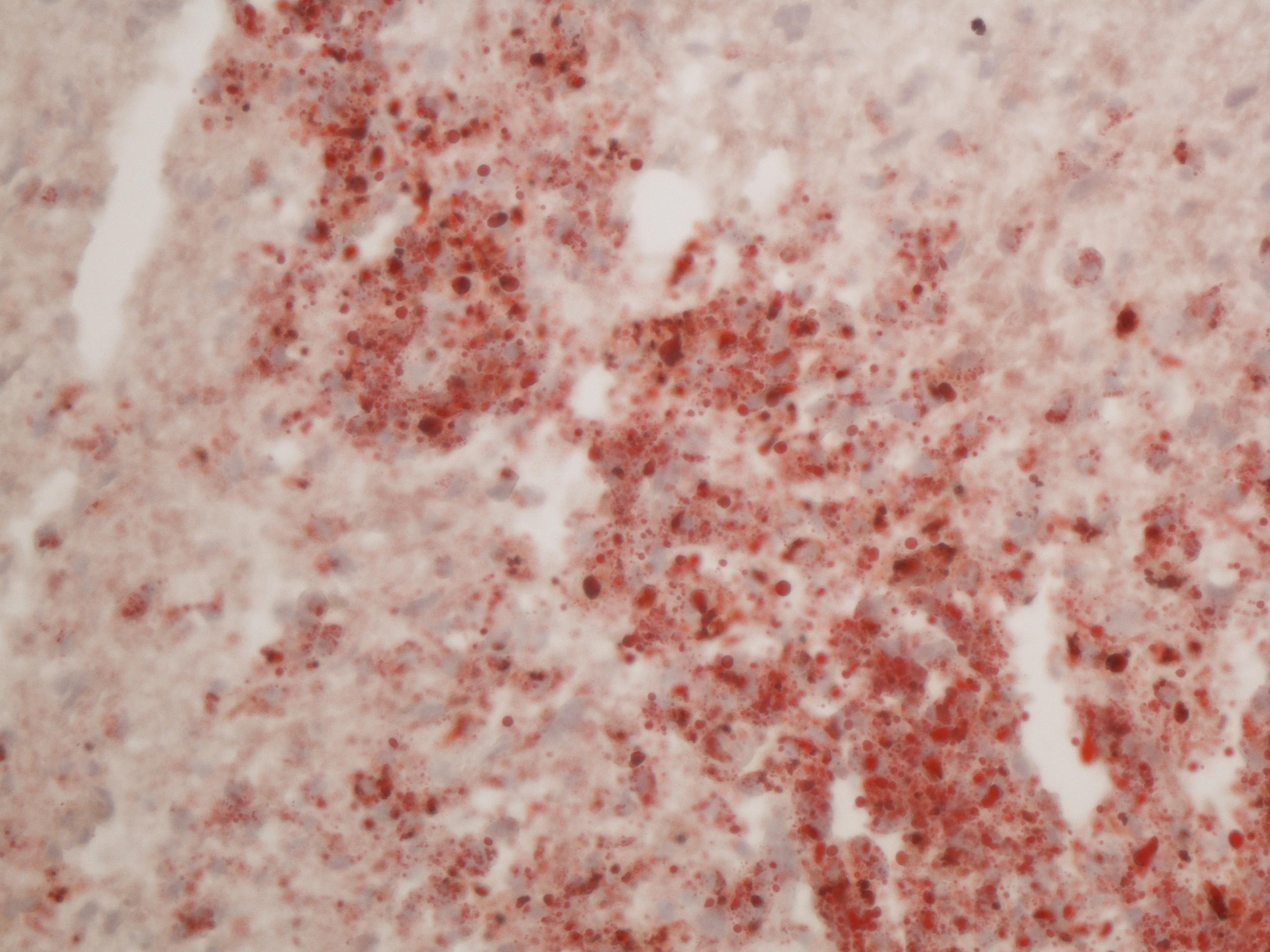

Supplement: Supplementary file 2 — Source Data for Appendix [file EMMM-14-e14759-s004.zip › Appendix Supplementary Data/Appendix Supplementary Figure 3/Oil Red O images/SEMA409A_40X.tif]

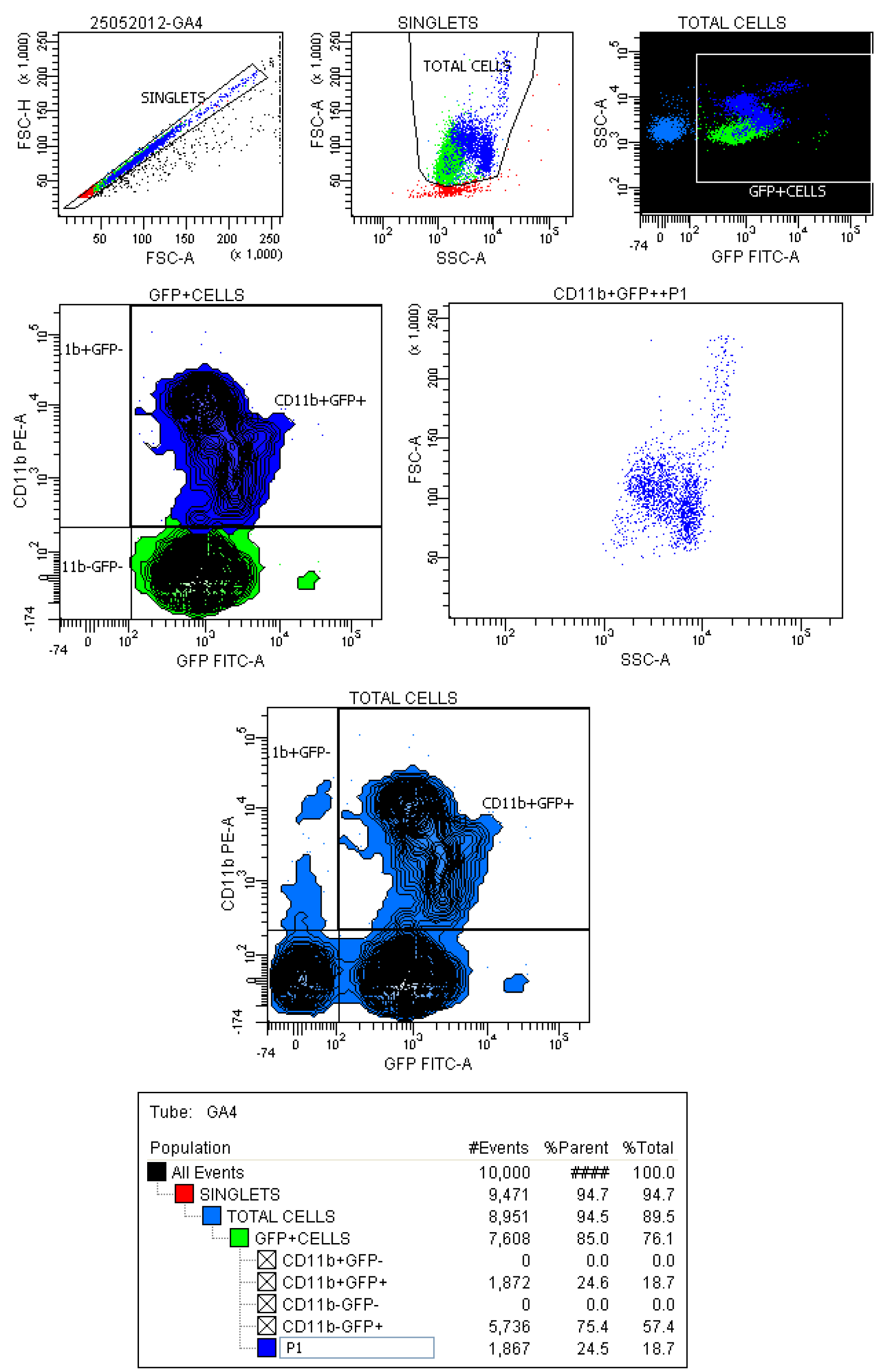

Supplement: Supplementary file 3 — Source Data for Figure 1 [file EMMM-14-e14759-s003.zip › Fig1/FACS GFP CD11b GA4.tif]

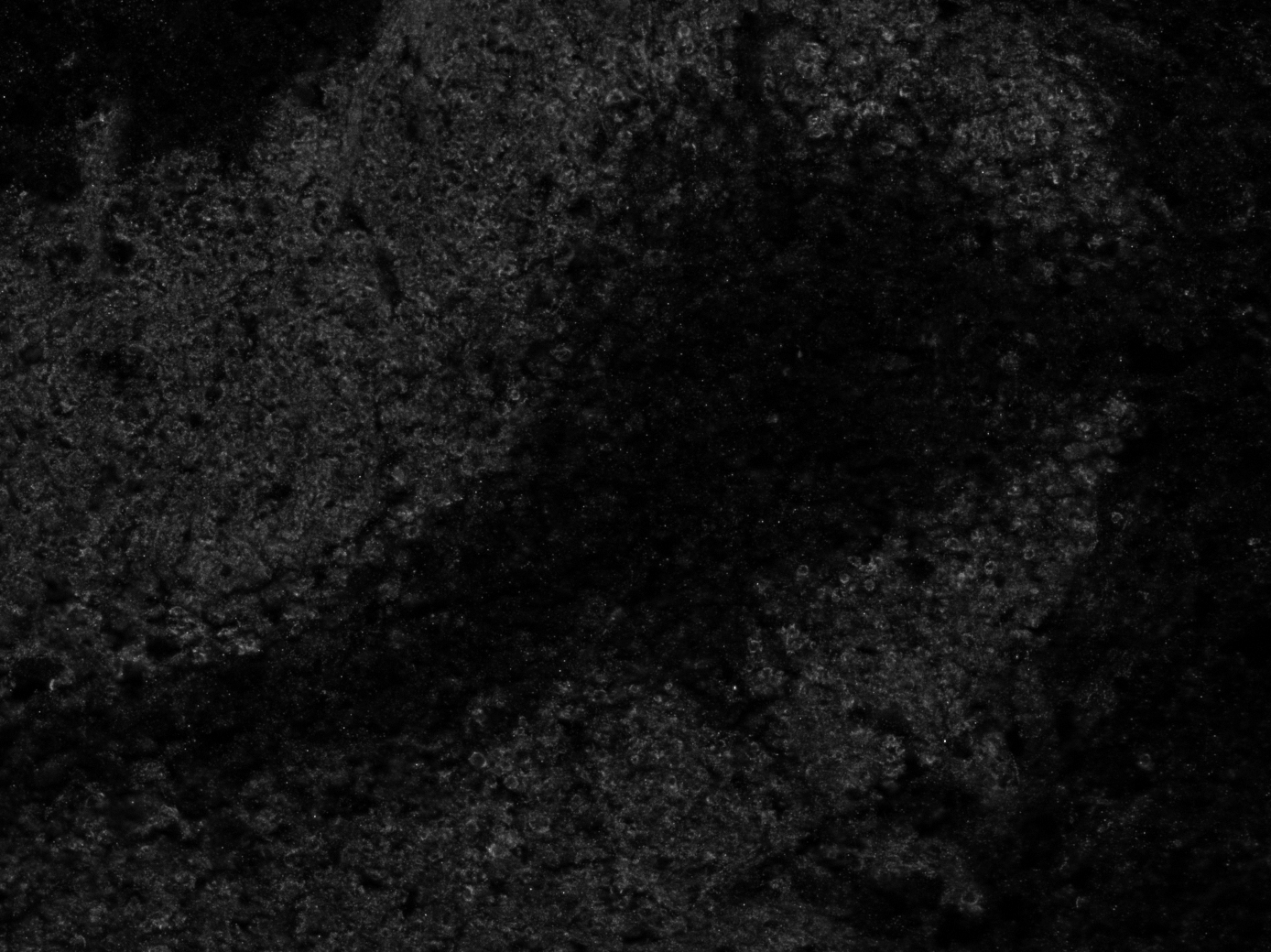

Supplement: Supplementary file 3 — Source Data for Figure 1 [file EMMM-14-e14759-s003.zip › Fig1/HSC3 3-3 x20 gfp cy5mbp red CM8_Cy5.tif]

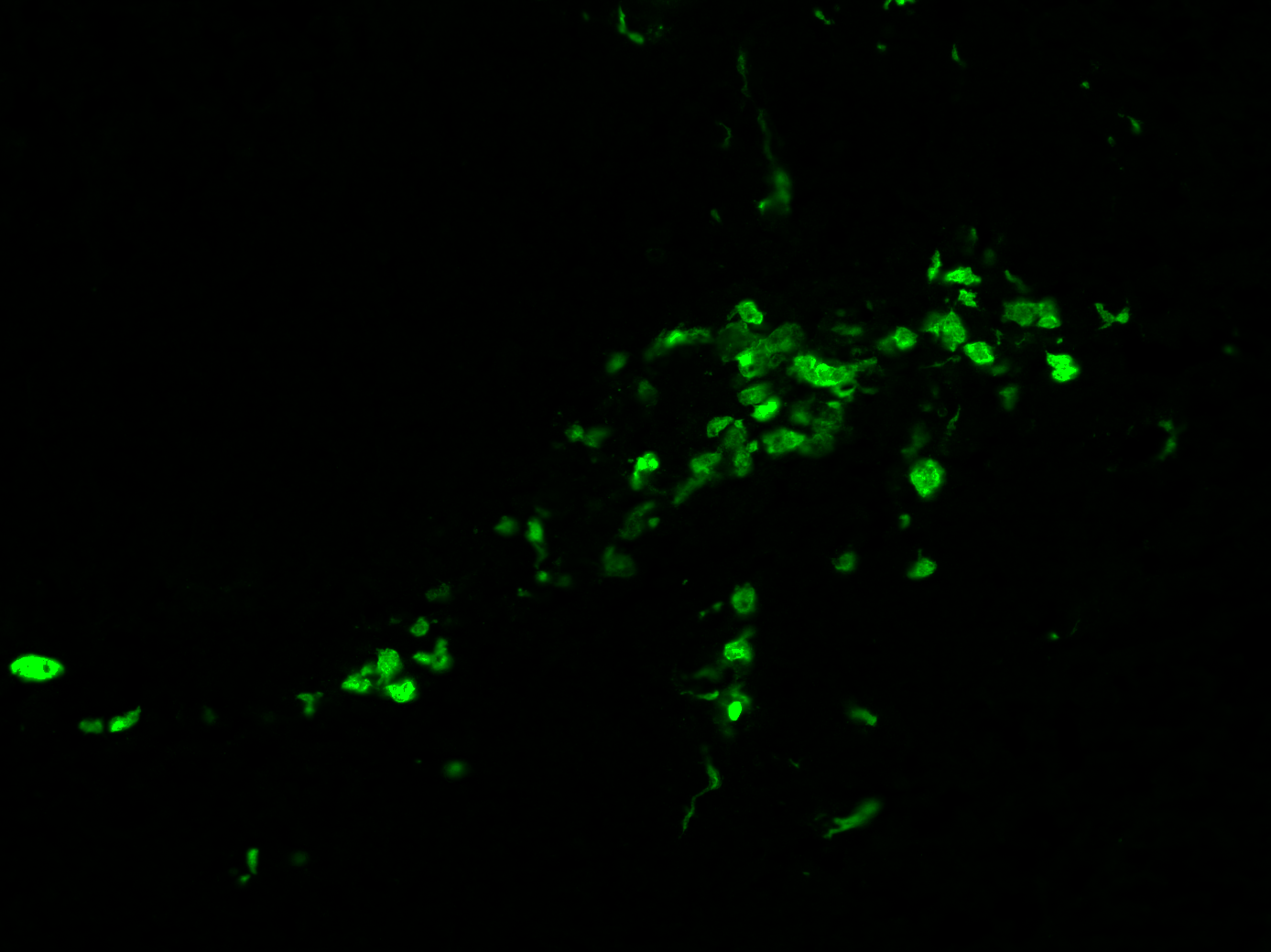

Supplement: Supplementary file 3 — Source Data for Figure 1 [file EMMM-14-e14759-s003.zip › Fig1/HSC3 3-3 x20 gfp cy5mbp red CM8_FITC.tif]

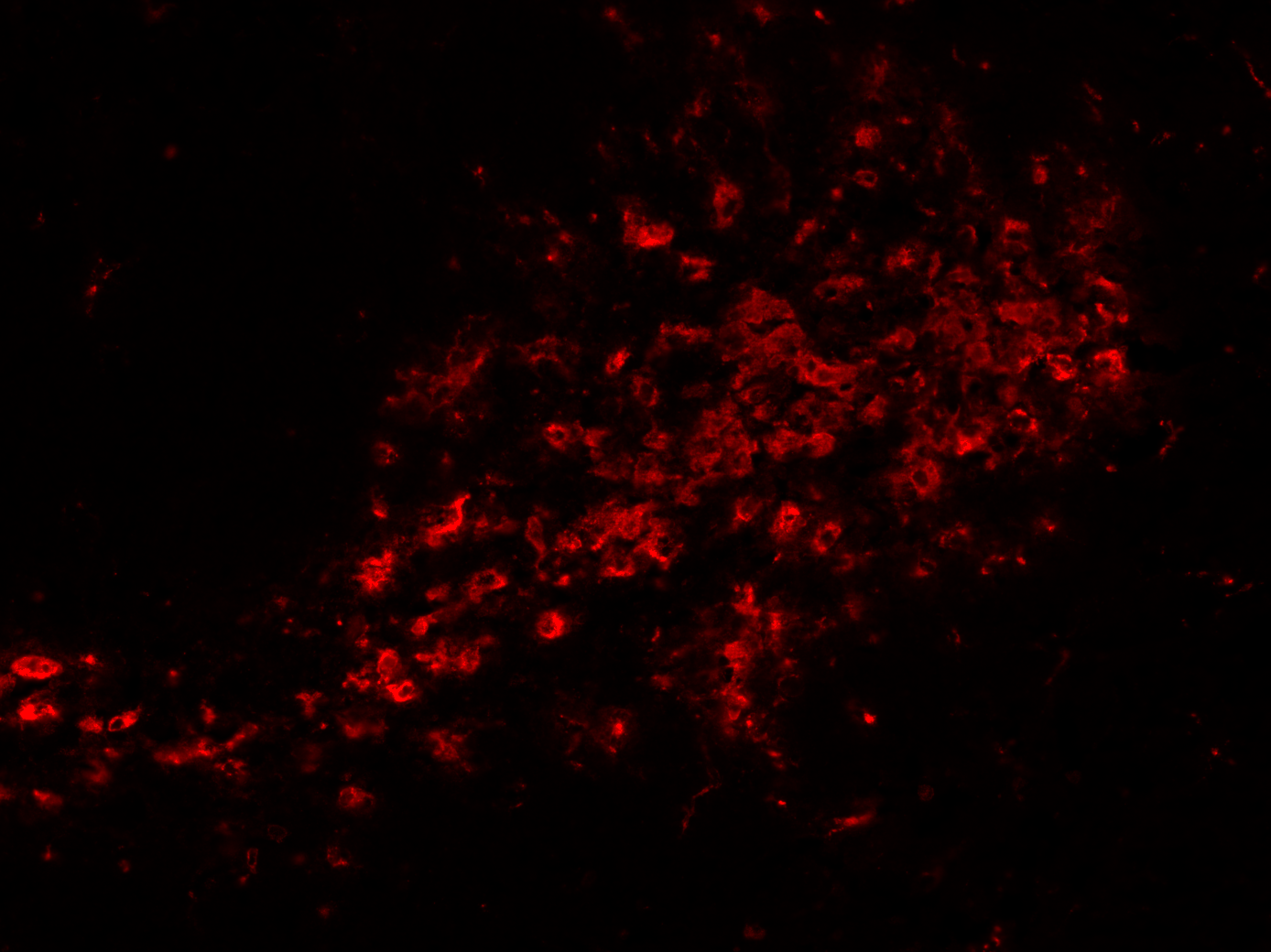

Supplement: Supplementary file 3 — Source Data for Figure 1 [file EMMM-14-e14759-s003.zip › Fig1/HSC3 3-3 x20 gfp cy5mbp red CM8_TRITC.tif]

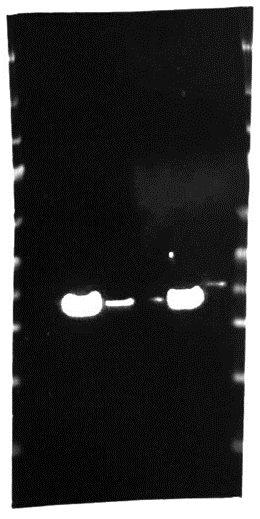

Supplement: Supplementary file 4 — Source Data for Figure 2 [file EMMM-14-e14759-s002.zip › Fig 2/Fig 2-images/WB GFP.png]

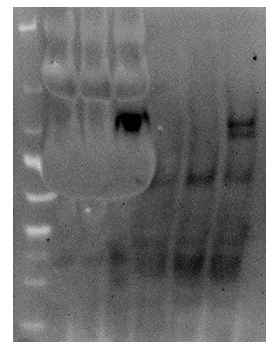

Supplement: Supplementary file 4 — Source Data for Figure 2 [file EMMM-14-e14759-s002.zip › Fig 2/Fig 2-images/WB Sema.png]

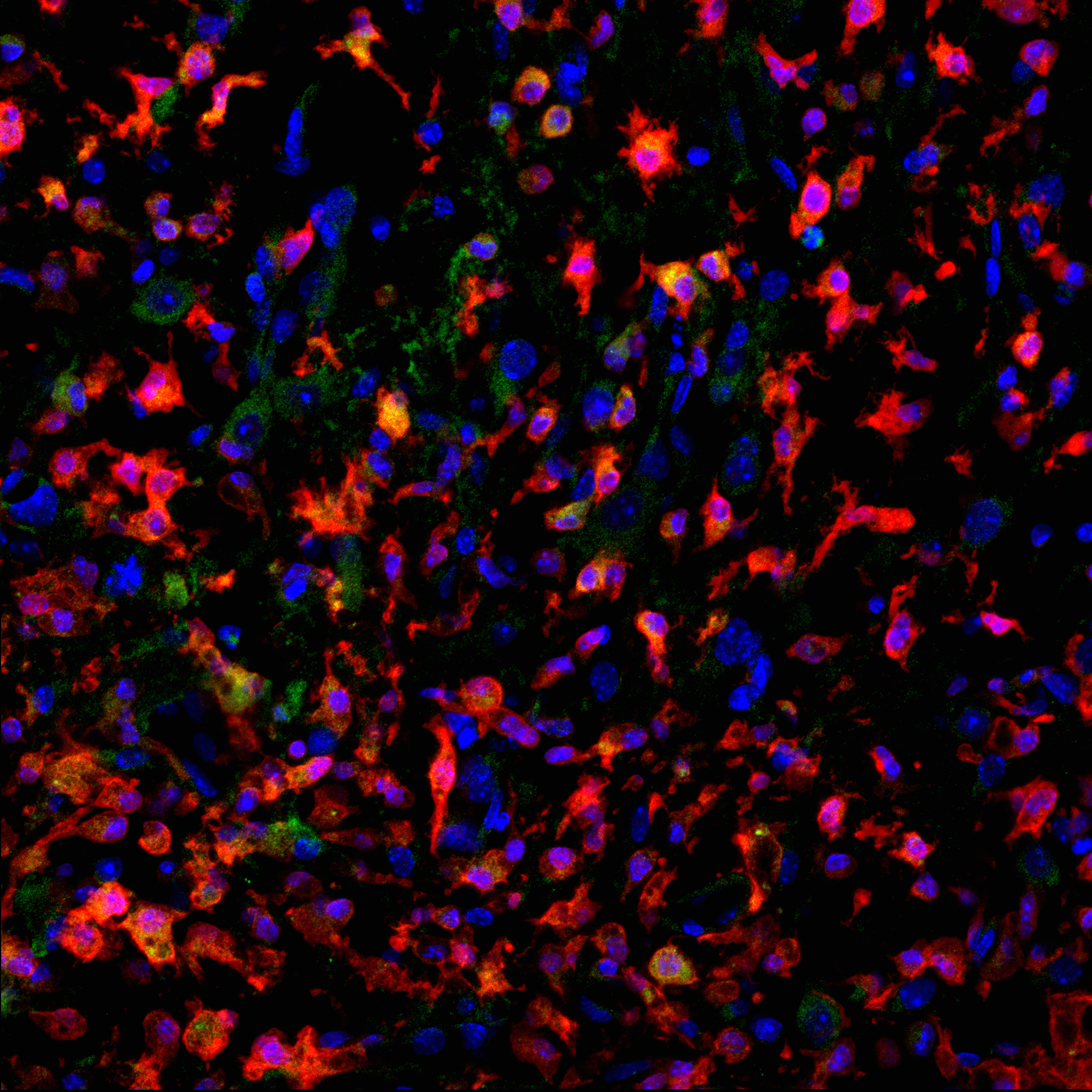

Supplement: Supplementary file 5 — Source Data for Figure 3 [file EMMM-14-e14759-s008.zip › Fig 3/images/Iba1(R)_Sema3F(G)_DAPI(B)_156_2_2m_4dpl.tif]

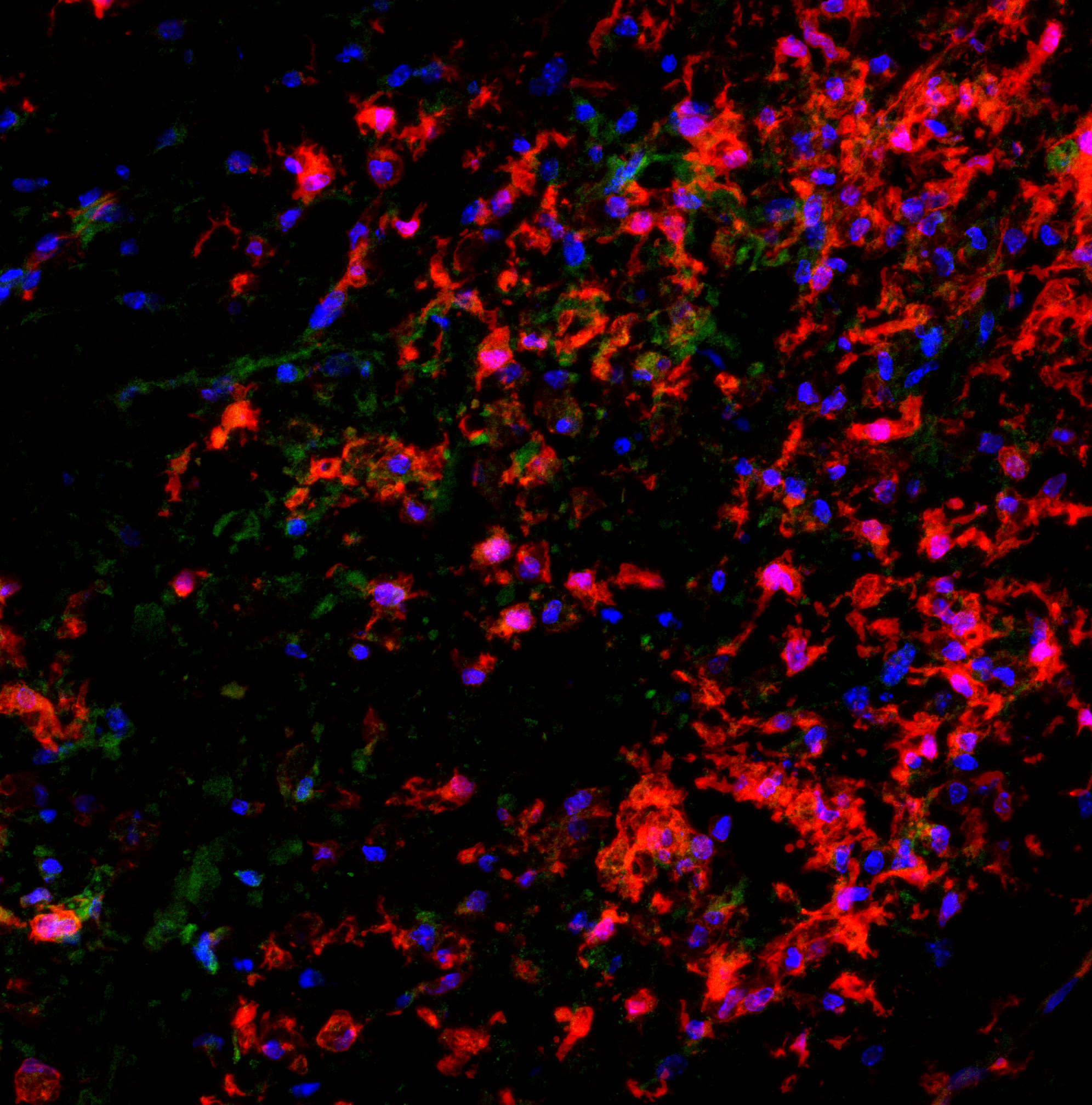

Supplement: Supplementary file 5 — Source Data for Figure 3 [file EMMM-14-e14759-s008.zip › Fig 3/images/Iba1(R)_Sema3F(G)_DAPI(B)_510_1_12m_4dpl.tif]

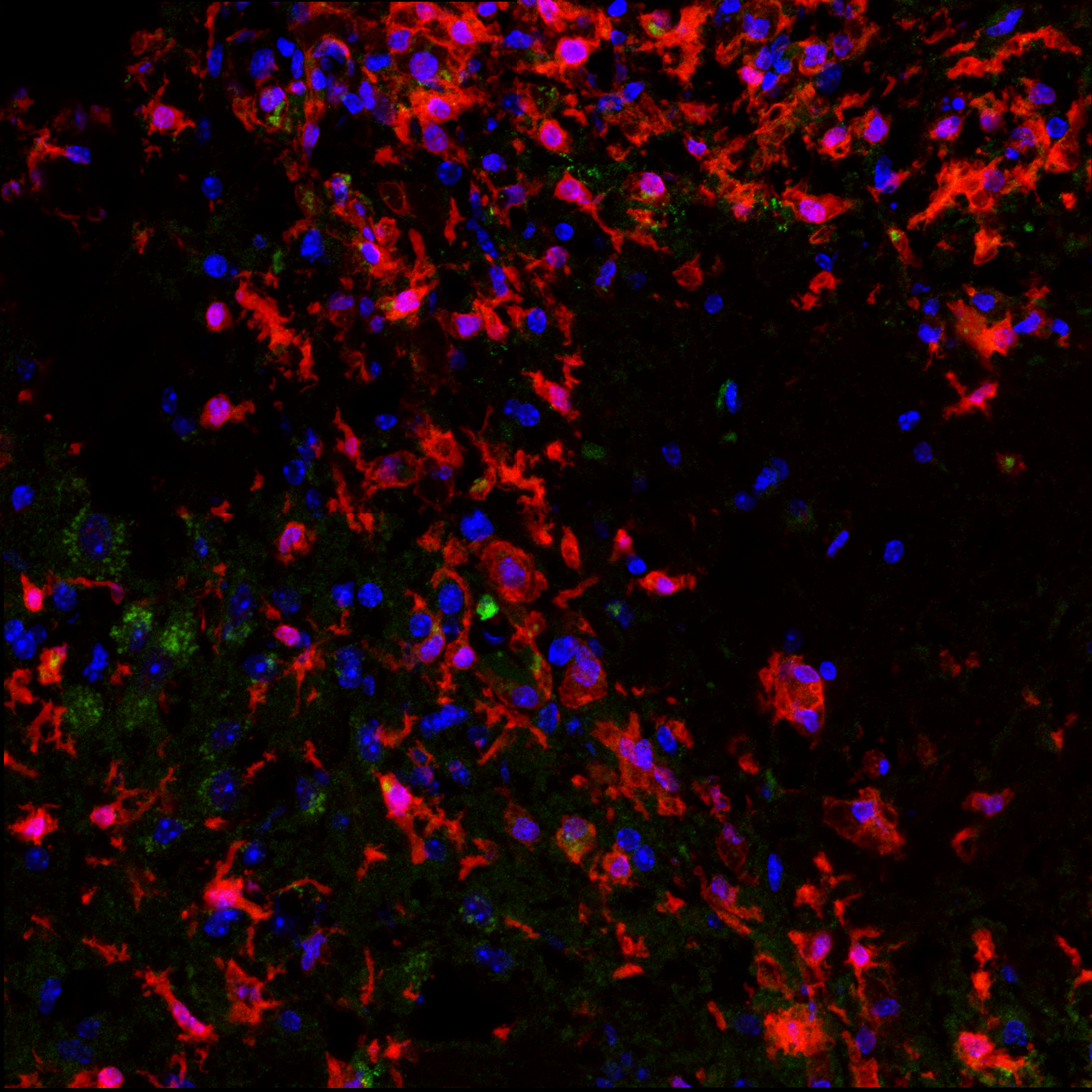

Supplement: Supplementary file 5 — Source Data for Figure 3 [file EMMM-14-e14759-s008.zip › Fig 3/images/Iba1(R)_Sema3F(G)_DAPI(B)_601_1_18m_4dpl.tiff]

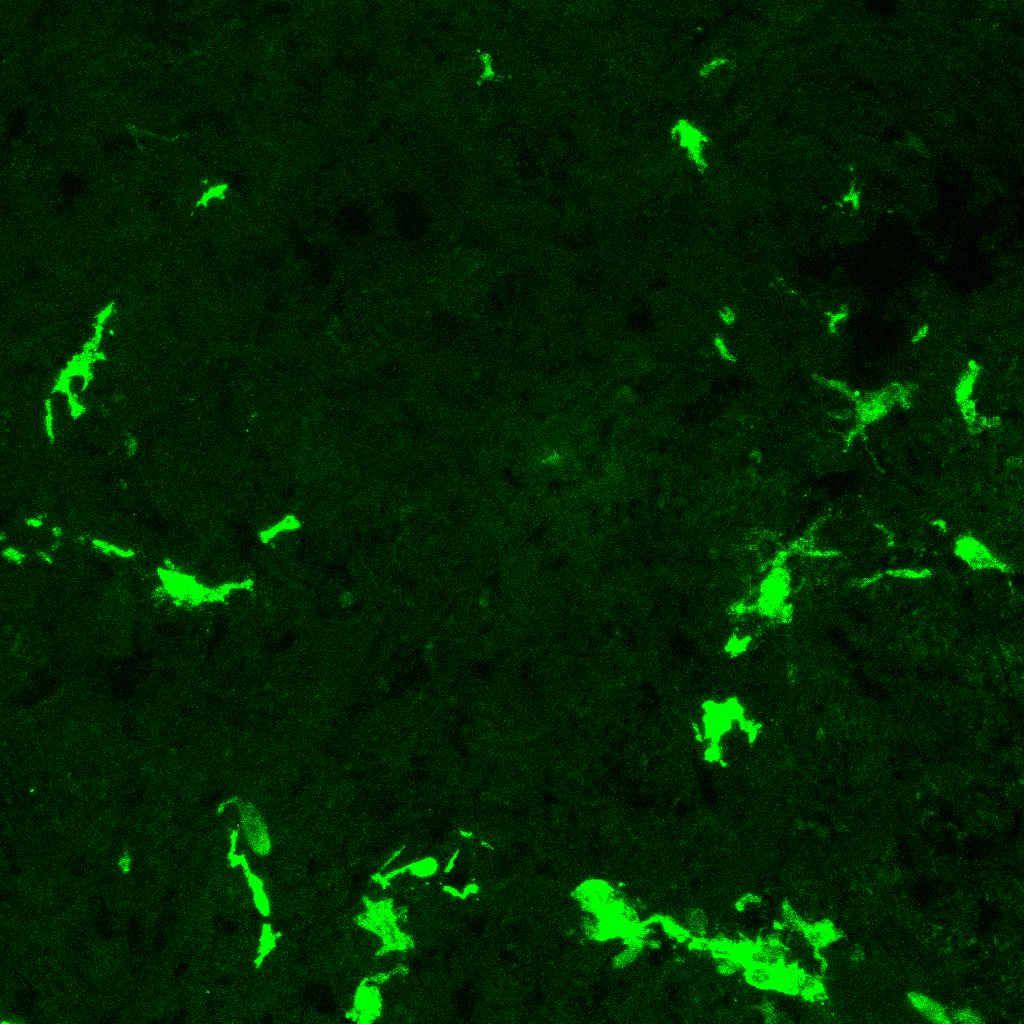

Supplement: Supplementary file 7 — Source Data for Figure 5 [file EMMM-14-e14759-s007.zip › Fig 5/Fig 5-images/GFP-CD220/C2-MAX_sema 7d gfp mbp b220 cd45r.lif - 909 gfpmbp b220 cd45r occasional B cell and not gfp001.tif]

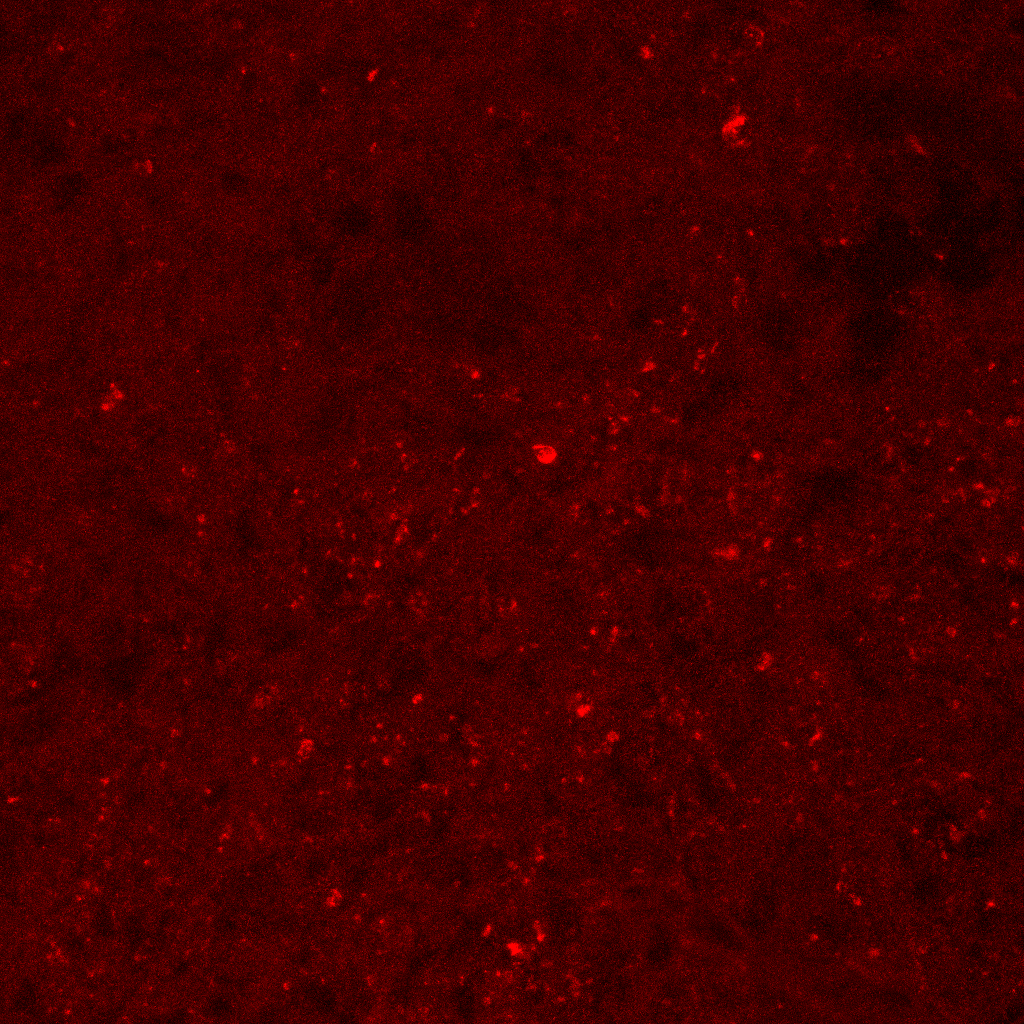

Supplement: Supplementary file 7 — Source Data for Figure 5 [file EMMM-14-e14759-s007.zip › Fig 5/Fig 5-images/GFP-CD220/C4-MAX_sema 7d gfp mbp b220 cd45r.lif - 909 gfpmbp b220 cd45r occasional B cell and not gfp001.tif]

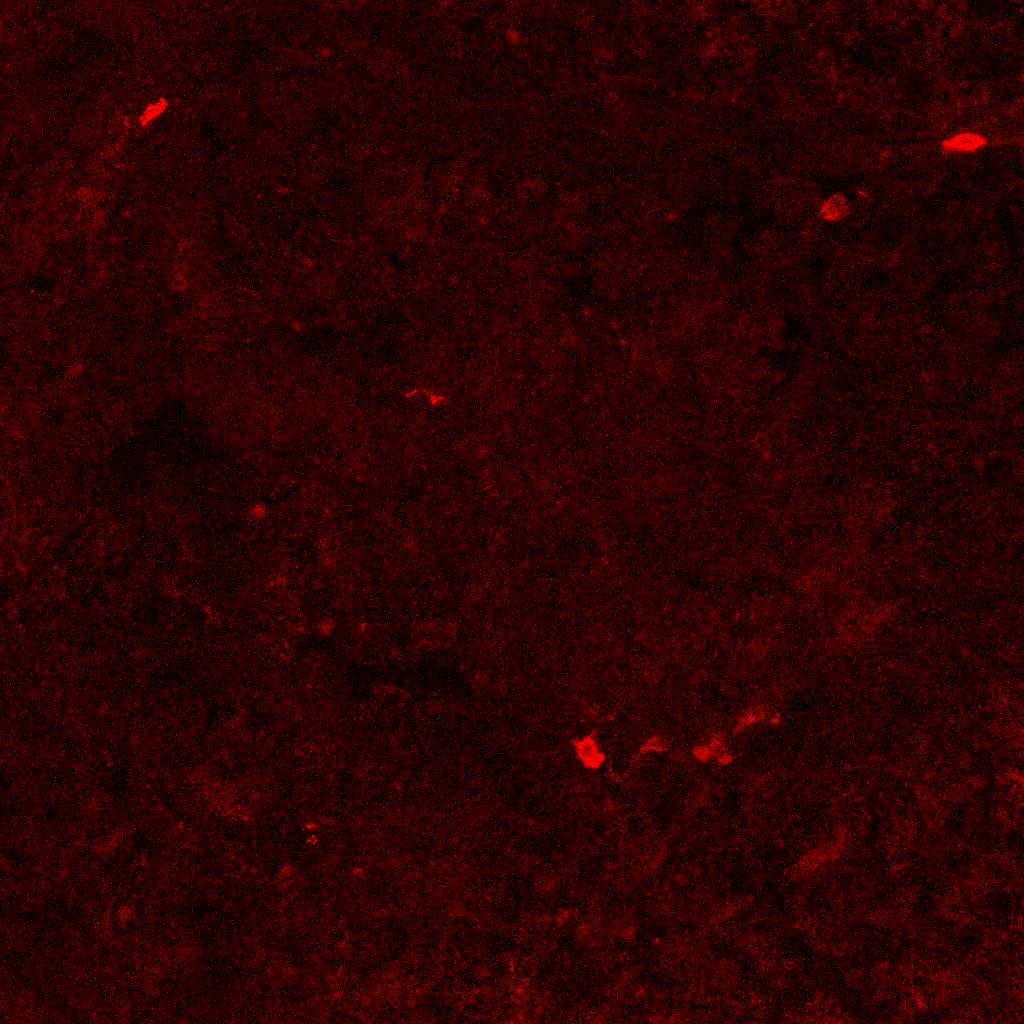

Supplement: Supplementary file 7 — Source Data for Figure 5 [file EMMM-14-e14759-s007.zip › Fig 5/Fig 5-images/GFP-CD3/Cd3 907 sema gfp mbp cd3 lymp001.tif]

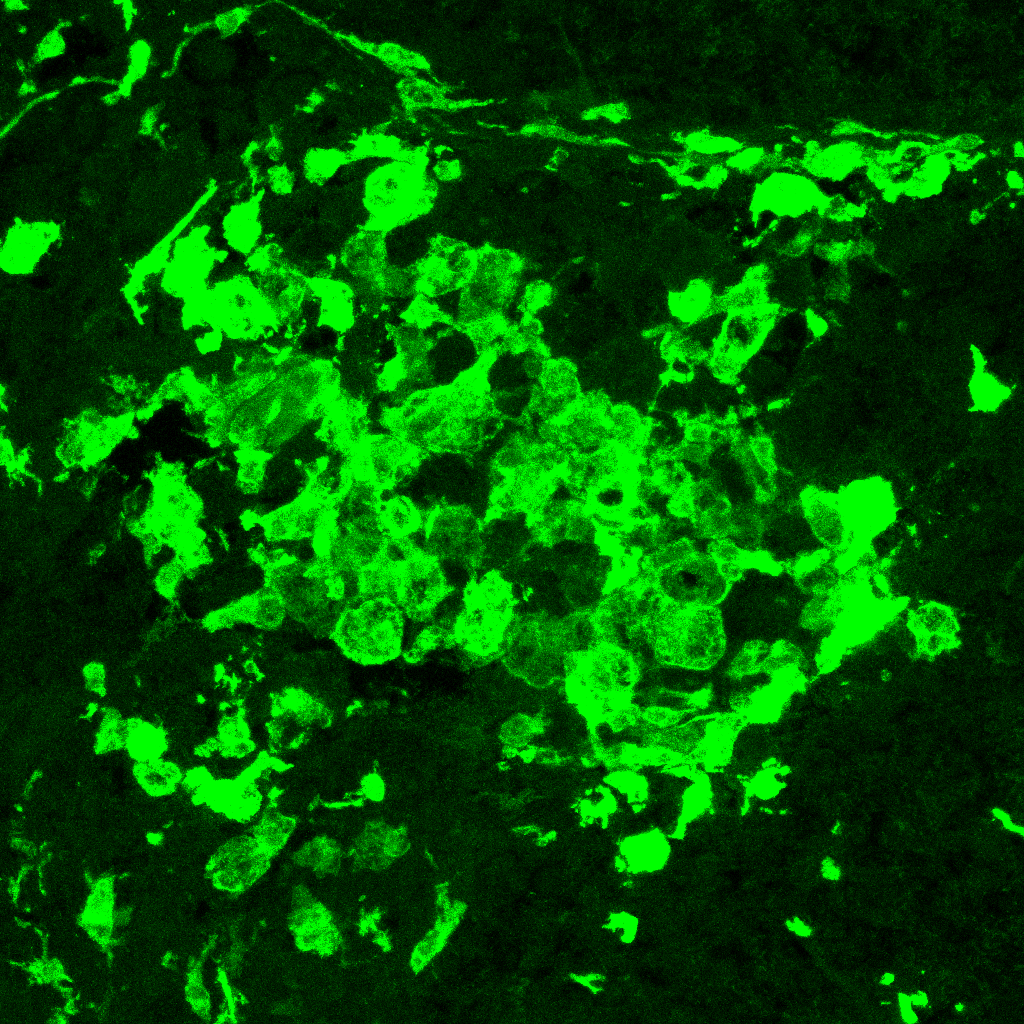

Supplement: Supplementary file 7 — Source Data for Figure 5 [file EMMM-14-e14759-s007.zip › Fig 5/Fig 5-images/GFP-CD3/GFP 907 sema gfp mbp cd3 lymp001.tif]

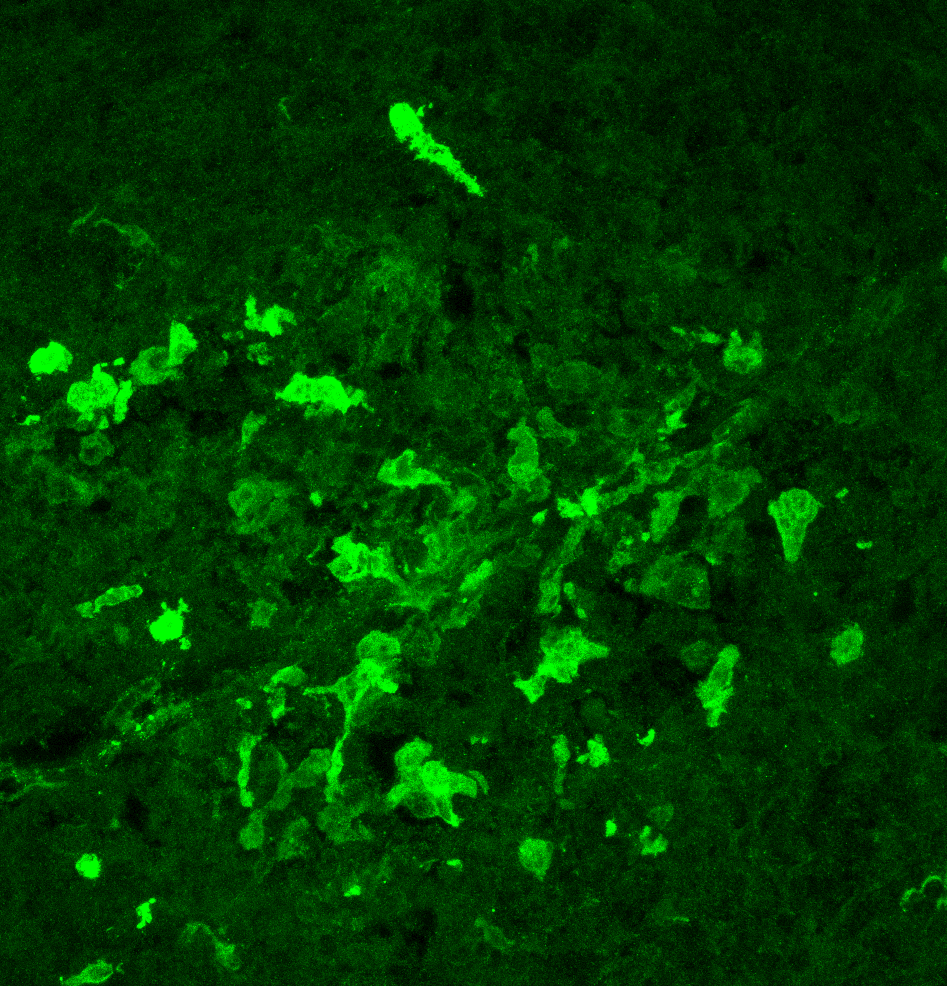

Supplement: Supplementary file 7 — Source Data for Figure 5 [file EMMM-14-e14759-s007.zip › Fig 5/Fig 5-images/GFP-P2Y12/GFP - 403 gfp 488 p2y12 647 MOG 555 IBA 594001.tif]

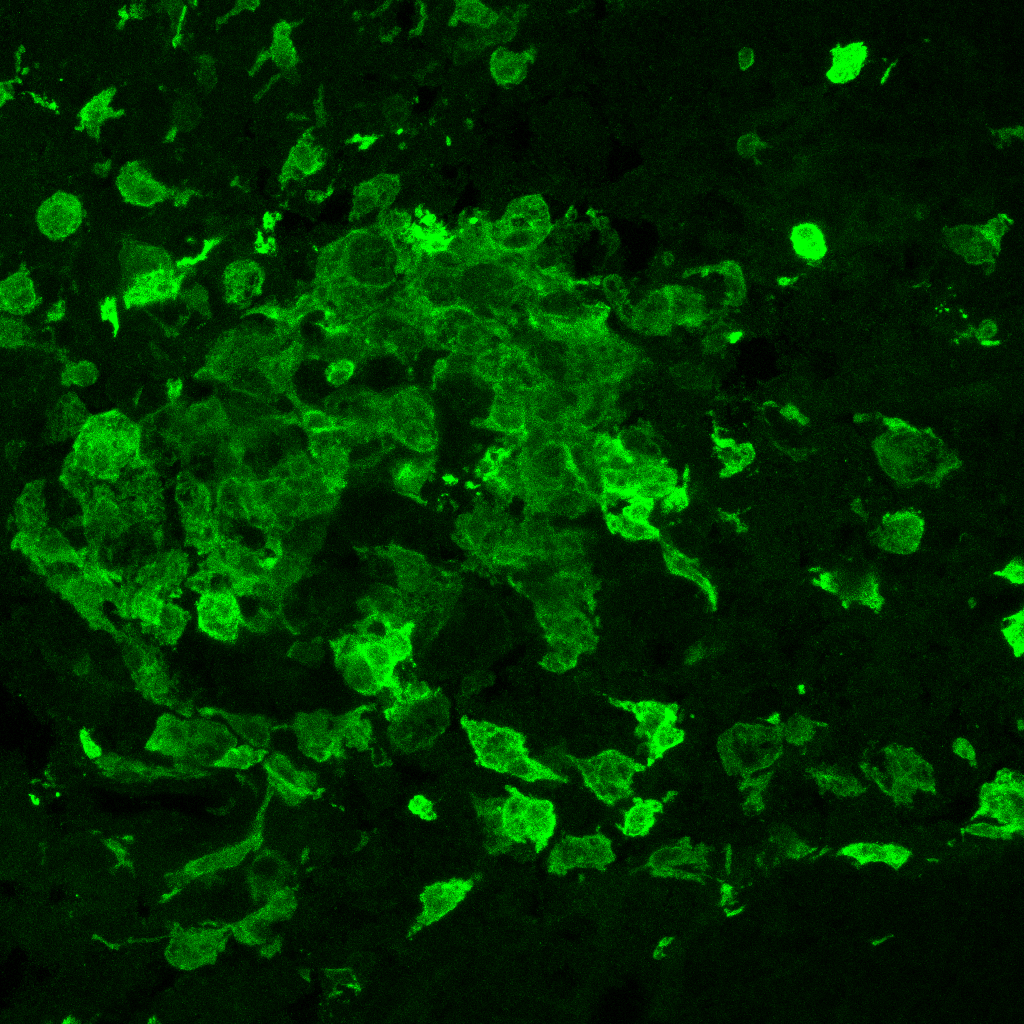

Supplement: Supplementary file 7 — Source Data for Figure 5 [file EMMM-14-e14759-s007.zip › Fig 5/Fig 5-images/GFP-P2Y12/GFP - 907 p2y 647 gfp 488 mog 555 iba1 594001.tif]

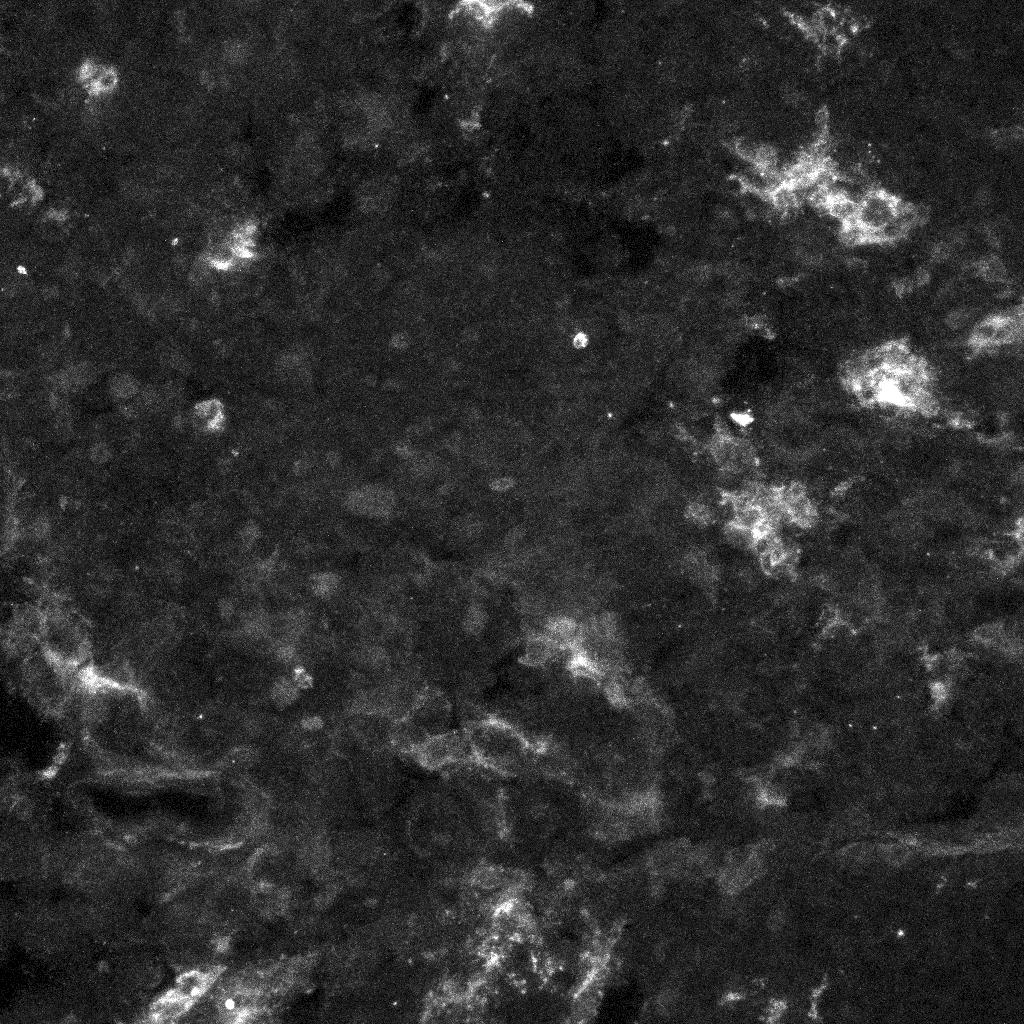

Supplement: Supplementary file 7 — Source Data for Figure 5 [file EMMM-14-e14759-s007.zip › Fig 5/Fig 5-images/GFP-P2Y12/P2Y- 907 p2y 647 gfp 488 mog 555 iba1 594001.tif]

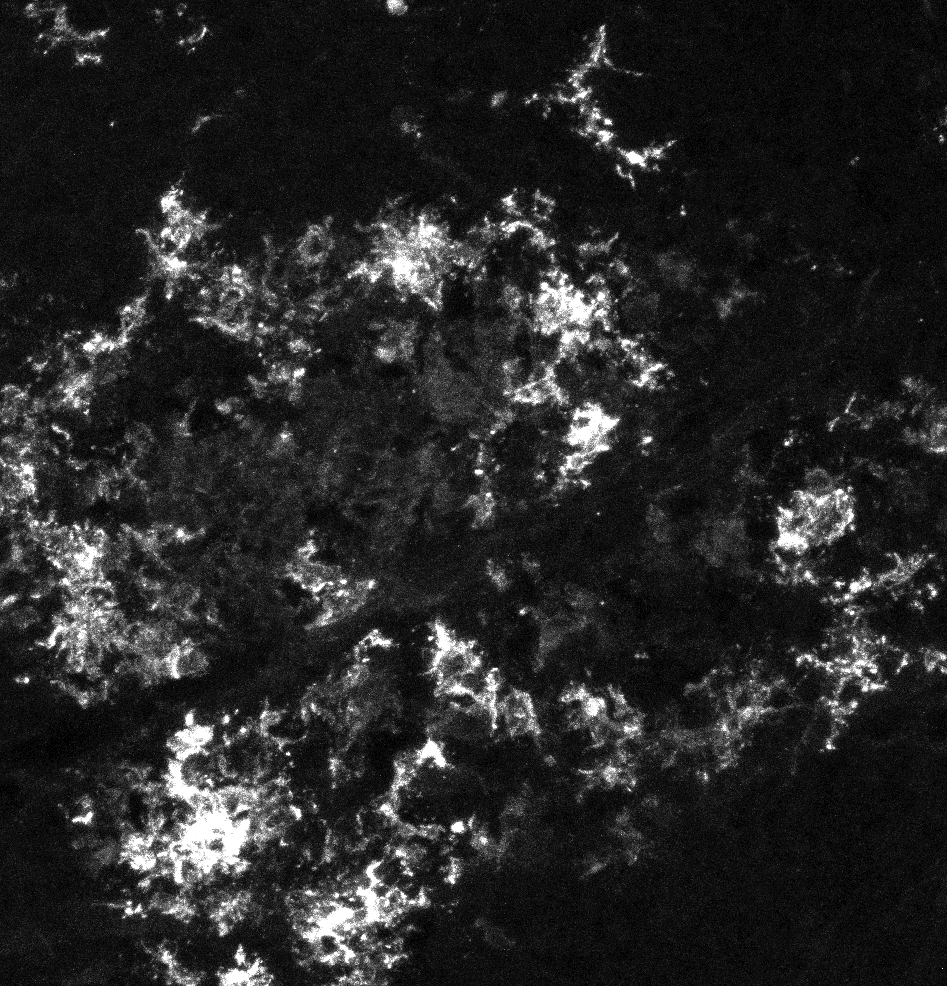

Supplement: Supplementary file 7 — Source Data for Figure 5 [file EMMM-14-e14759-s007.zip › Fig 5/Fig 5-images/GFP-P2Y12/P2Y12 - 403 gfp 488 p2y12 647 MOG 555 IBA 594001.tif]

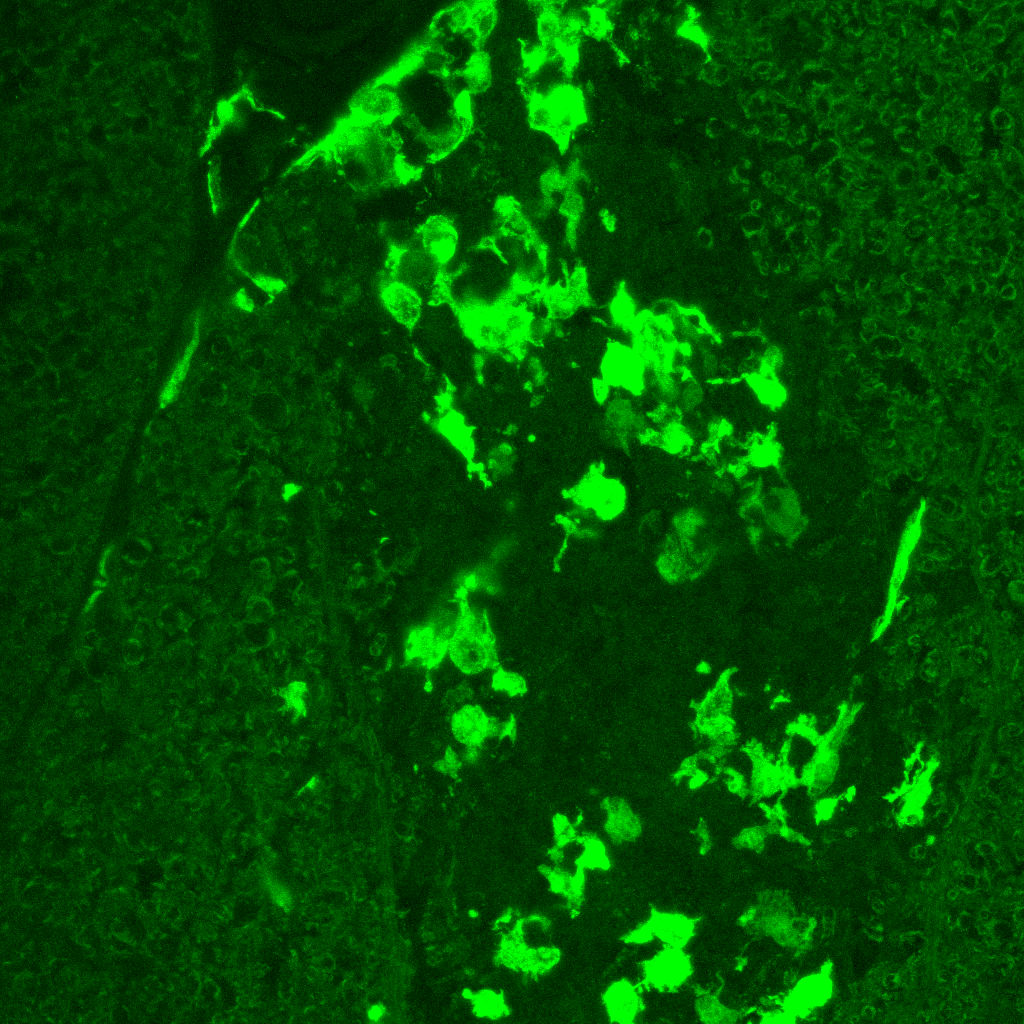

Supplement: Supplementary file 7 — Source Data for Figure 5 [file EMMM-14-e14759-s007.zip › Fig 5/Fig 5-images/illustrations GFP numbers/GFP and MBP 161 5-4.tif]

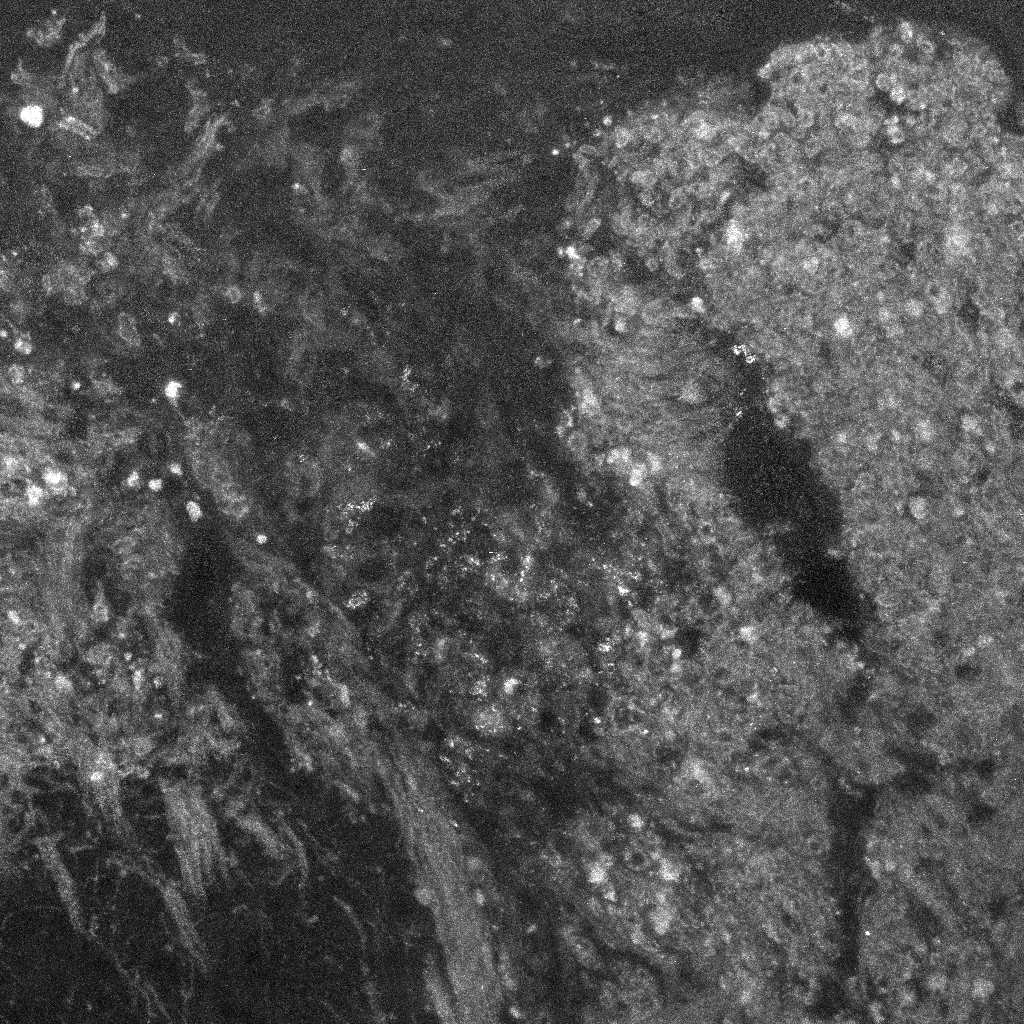

Supplement: Supplementary file 7 — Source Data for Figure 5 [file EMMM-14-e14759-s007.zip › Fig 5/Fig 5-images/illustrations GFP numbers/MBP 21B gfp rb iba 594 mbp 647x40001.tif]

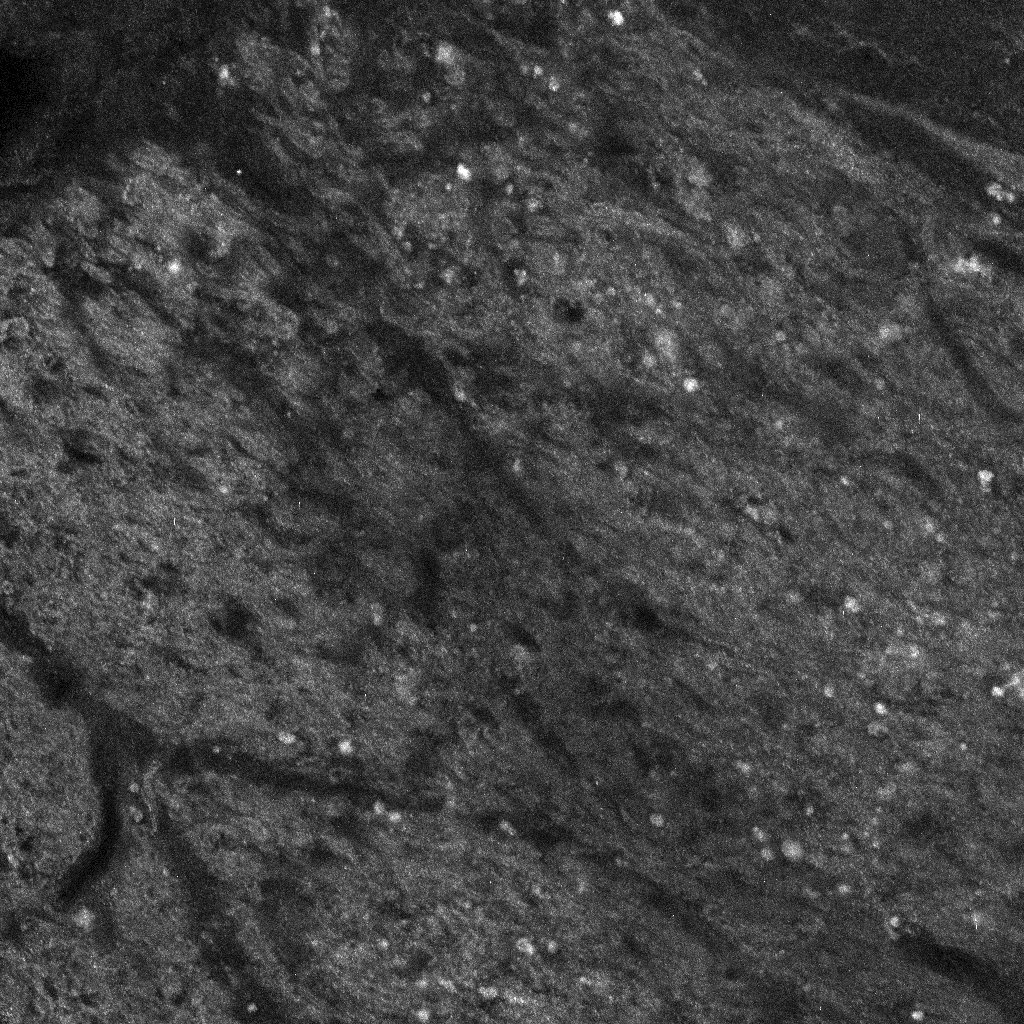

Supplement: Supplementary file 7 — Source Data for Figure 5 [file EMMM-14-e14759-s007.zip › Fig 5/Fig 5-images/illustrations GFP numbers/MBP 24 gfp rb iba 594 mbp 647x40001.tif]

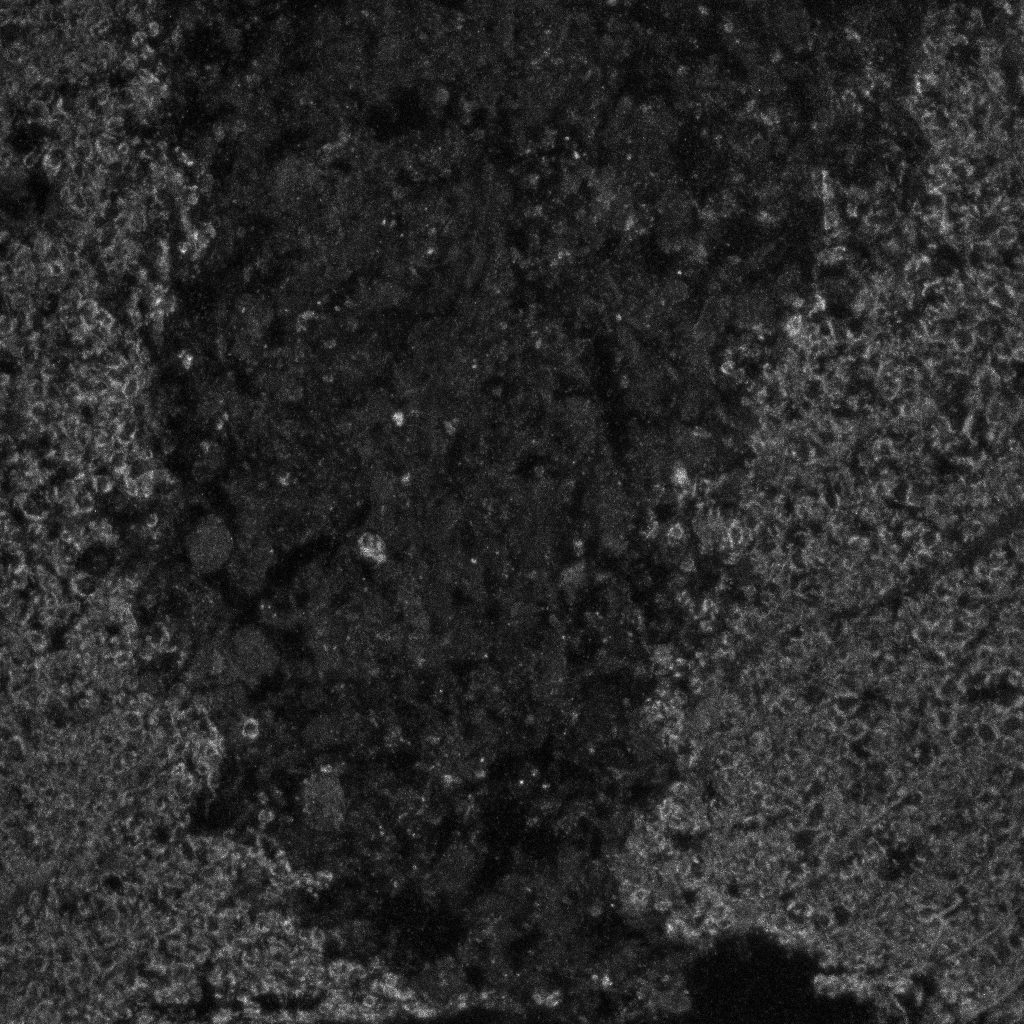

Supplement: Supplementary file 7 — Source Data for Figure 5 [file EMMM-14-e14759-s007.zip › Fig 5/Fig 5-images/illustrations GFP numbers/mbp-MAX_Sema Olig2.lif - 901 3-4A.tif]

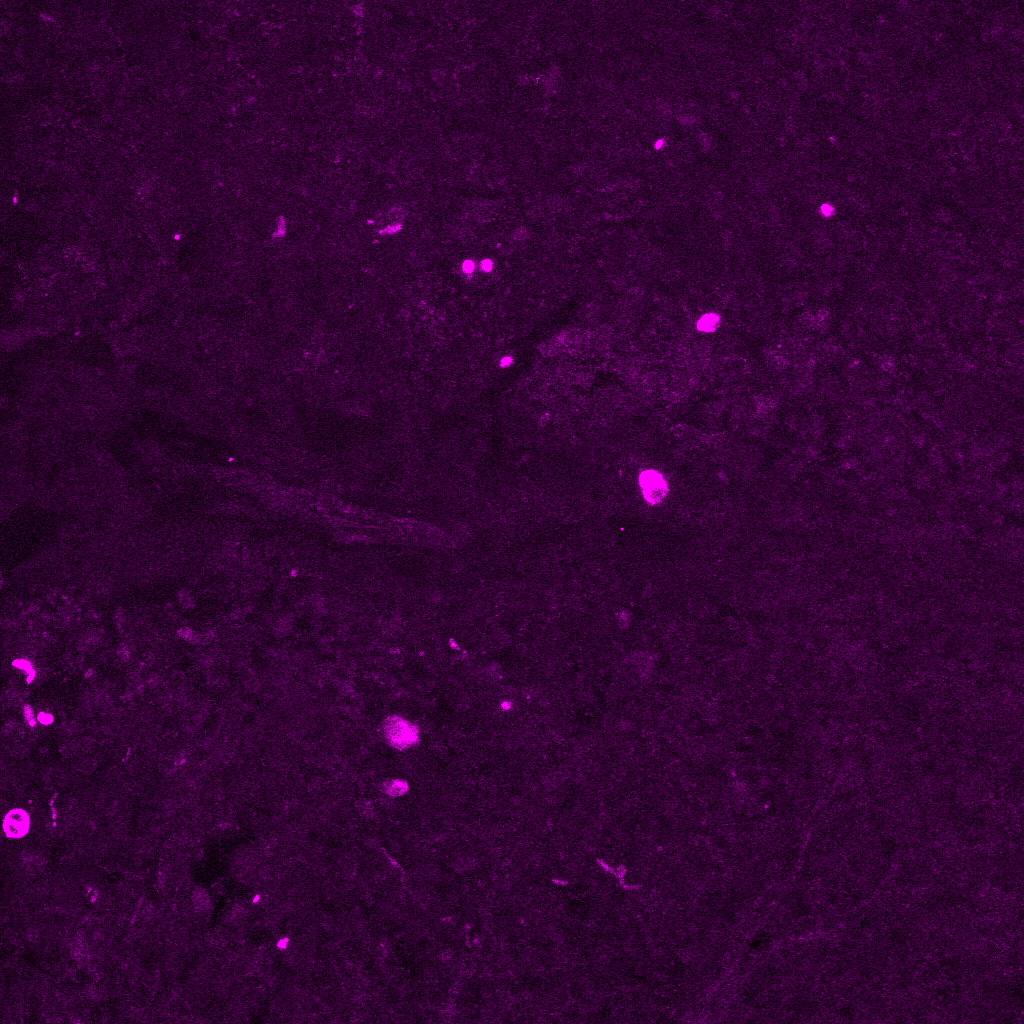

Supplement: Supplementary file 8 — Source Data for Figure 6 [file EMMM-14-e14759-s006.zip › Fig 6/Fig 6-images/casp3olig2/C3-MAX_sema 7d casp3olig2.lif - 907 ventral use 3-2 mbp555 casp3 594 olig2 647001.tif]

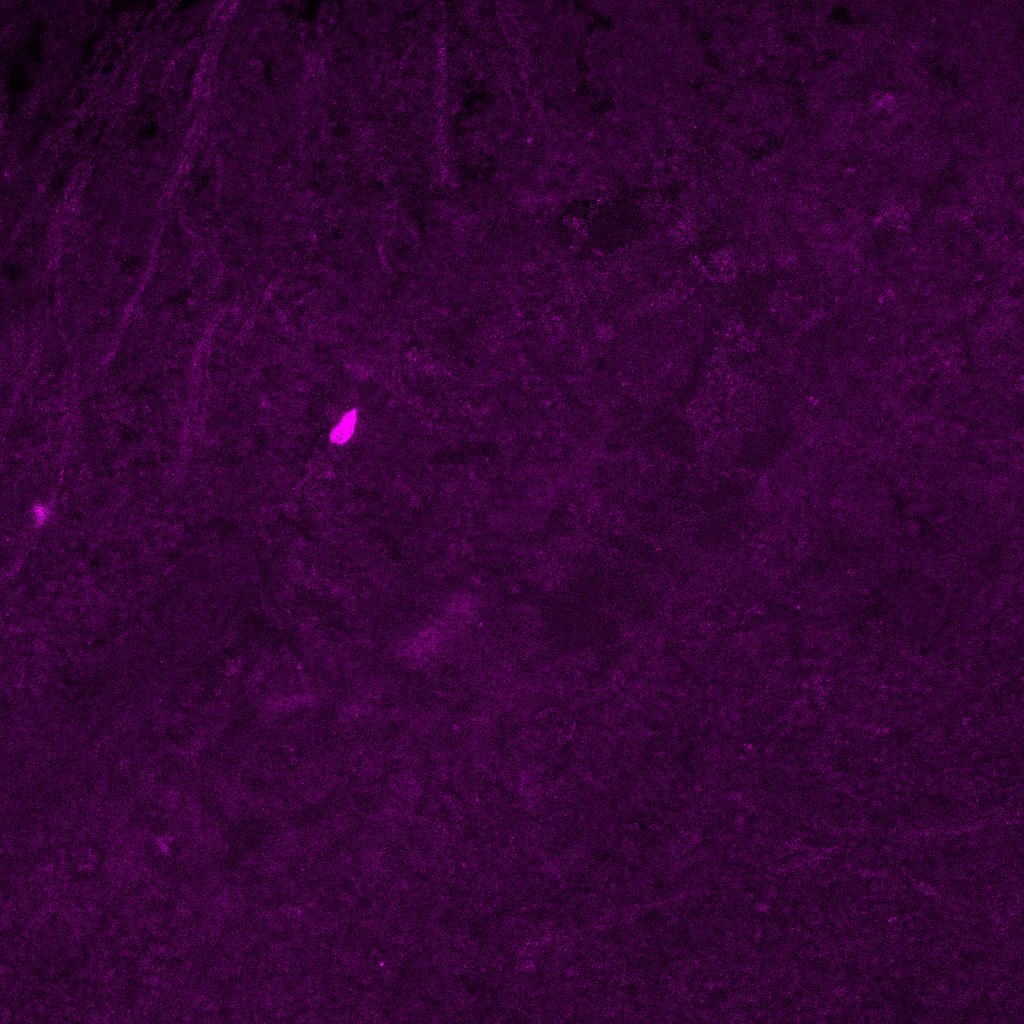

Supplement: Supplementary file 8 — Source Data for Figure 6 [file EMMM-14-e14759-s006.zip › Fig 6/Fig 6-images/casp3olig2/C3-MAX_sema 7d casp3olig2.lif - 908 4-2 mbp555 casp3 594 olig2 647001.tif]

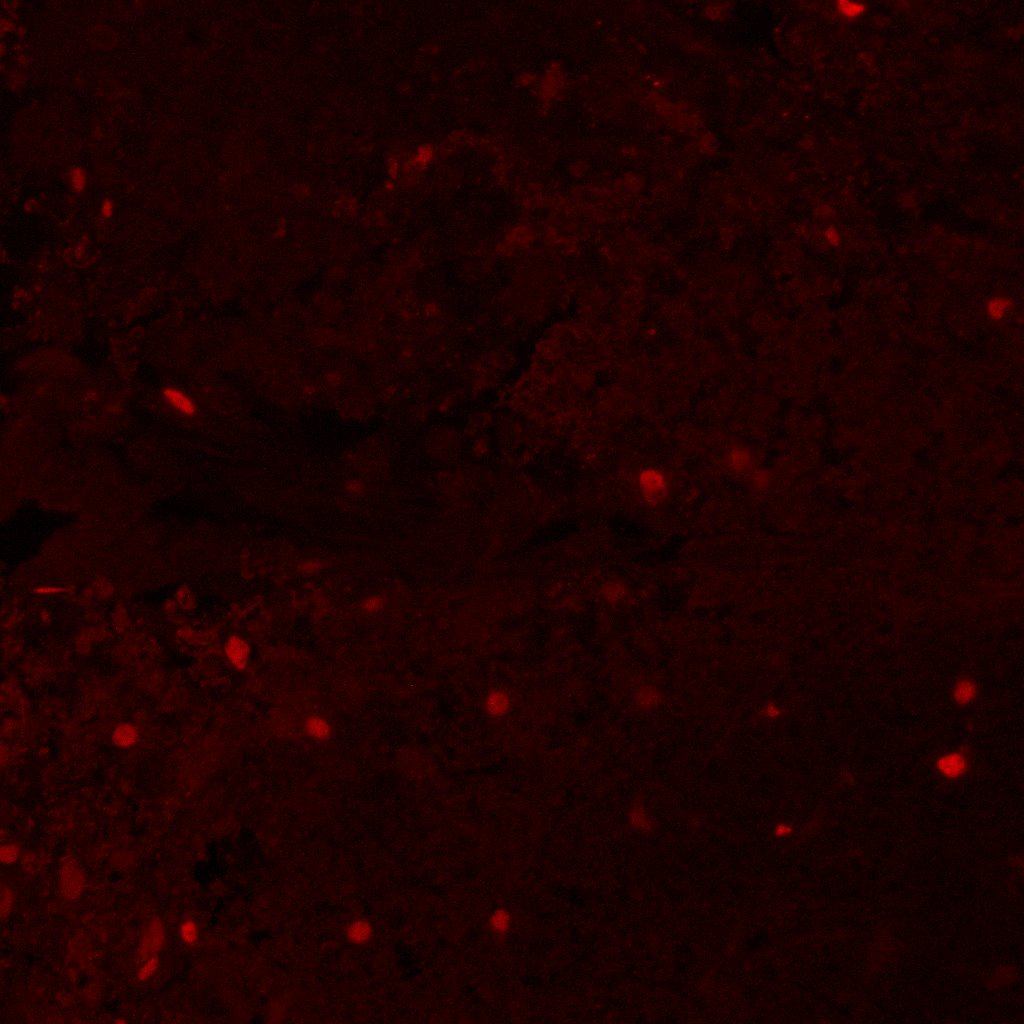

Supplement: Supplementary file 8 — Source Data for Figure 6 [file EMMM-14-e14759-s006.zip › Fig 6/Fig 6-images/casp3olig2/C4-MAX_sema 7d casp3olig2.lif - 907 ventral use 3-2 mbp555 casp3 594 olig2 647001.tif]

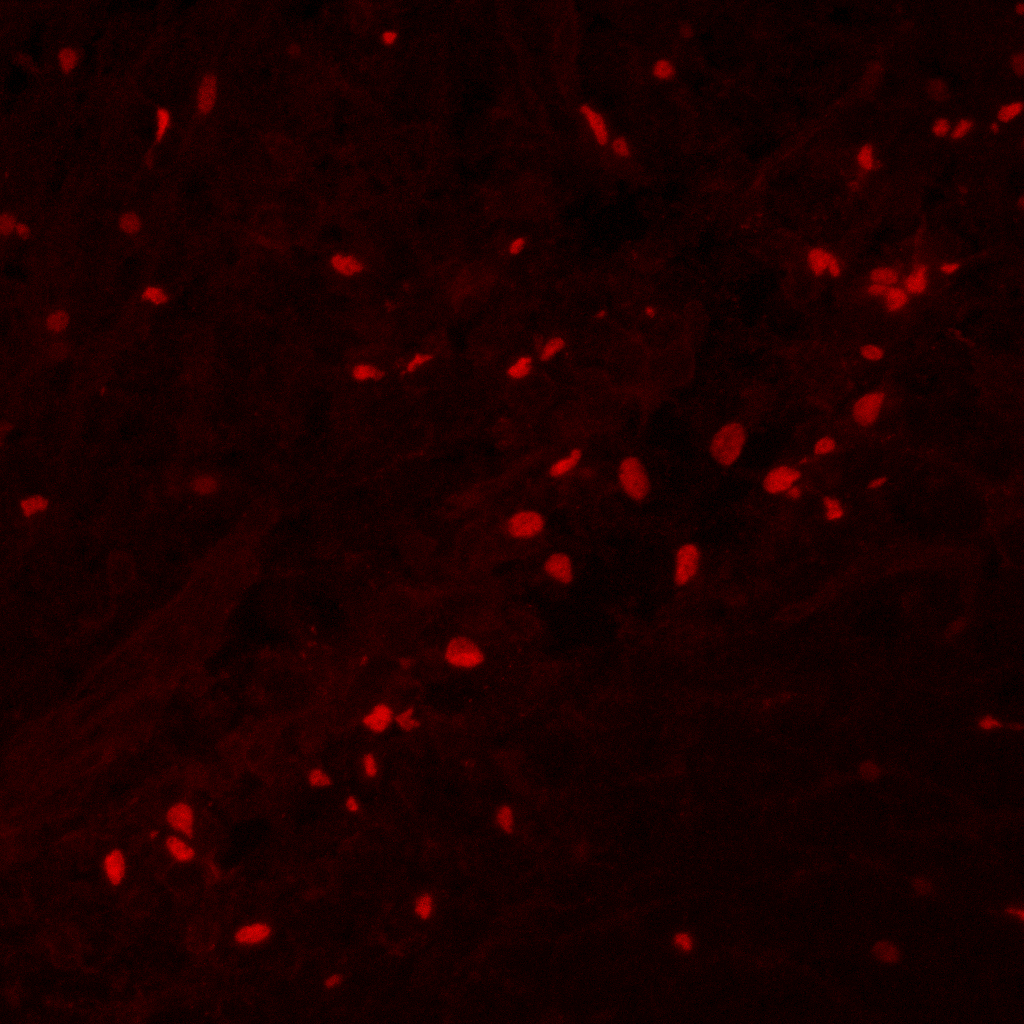

Supplement: Supplementary file 8 — Source Data for Figure 6 [file EMMM-14-e14759-s006.zip › Fig 6/Fig 6-images/casp3olig2/C4-MAX_sema 7d casp3olig2.lif - 908 4-2 mbp555 casp3 594 olig2 647001.tif]

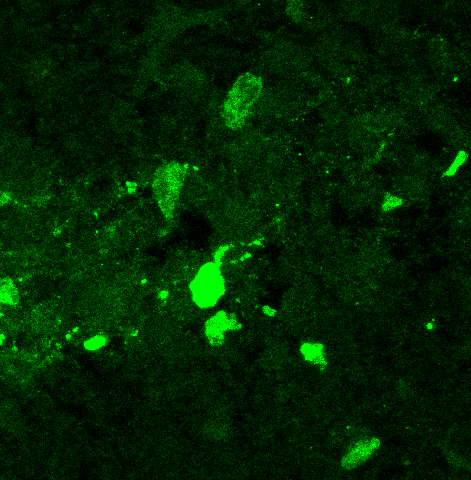

Supplement: Supplementary file 8 — Source Data for Figure 6 [file EMMM-14-e14759-s006.zip › Fig 6/Fig 6-images/Nkx2.2-GFP/GFP 897 NKX 594 KI67 647 MOG 555 GFP001-1.tif]

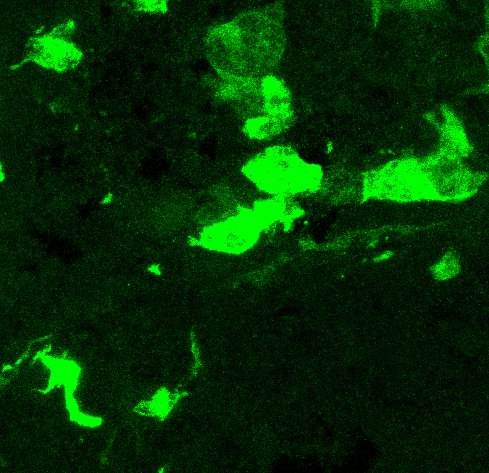

Supplement: Supplementary file 8 — Source Data for Figure 6 [file EMMM-14-e14759-s006.zip › Fig 6/Fig 6-images/Nkx2.2-GFP/GFP 907b VENT NKX 594 KI67 647 MOG 555 GFP001-1.tif]

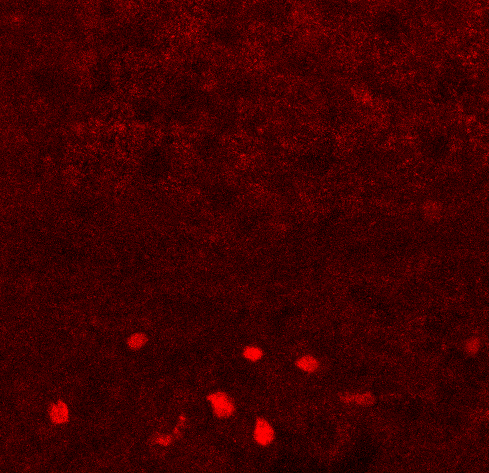

Supplement: Supplementary file 8 — Source Data for Figure 6 [file EMMM-14-e14759-s006.zip › Fig 6/Fig 6-images/Nkx2.2-GFP/Nkx 907b VENT NKX 594 KI67 647 MOG 555 GFP001-1.tif]

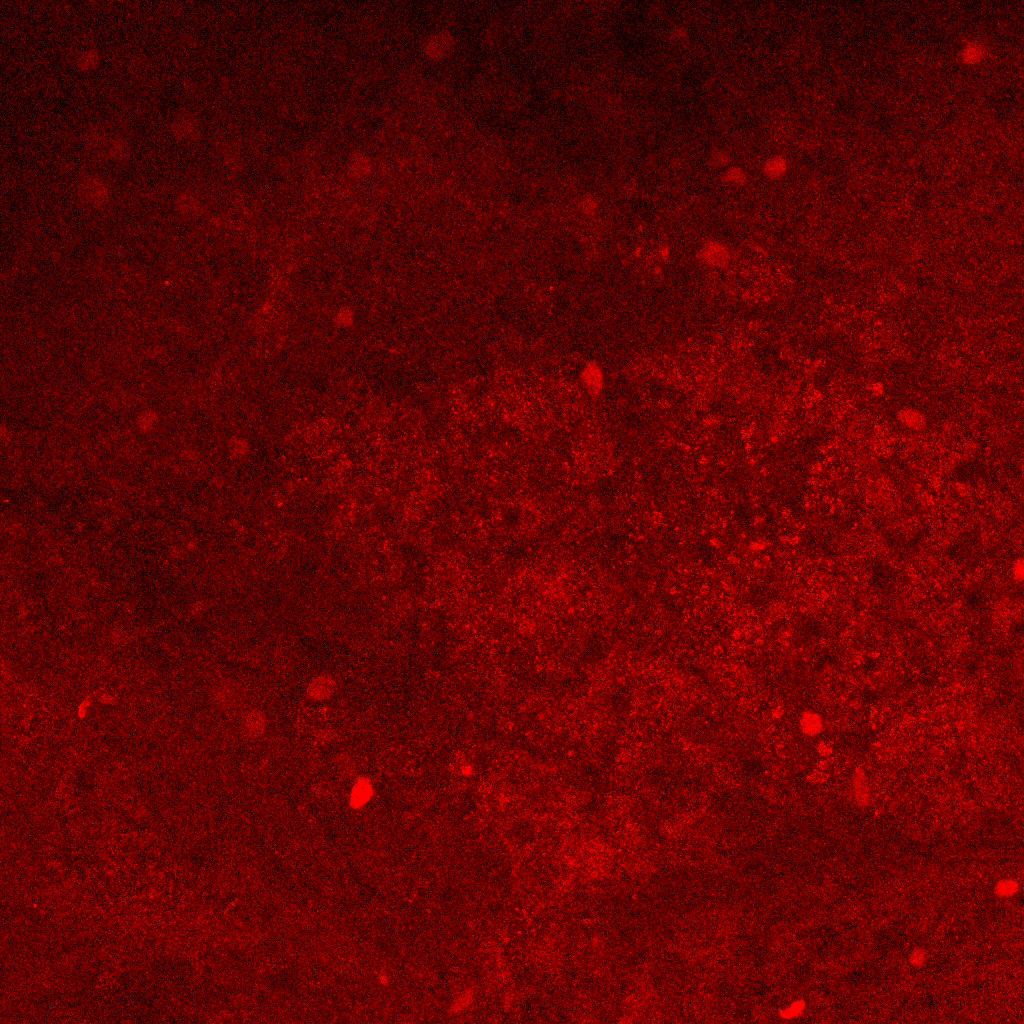

Supplement: Supplementary file 8 — Source Data for Figure 6 [file EMMM-14-e14759-s006.zip › Fig 6/Fig 6-images/NKX2.2-Ki67/C3-MAX_SEMA 7 D NKX 594 KI67 647 MOG 555 GFP.lif - 907 NKX 594 KI67 647 MOG 555 GFP001.tif]

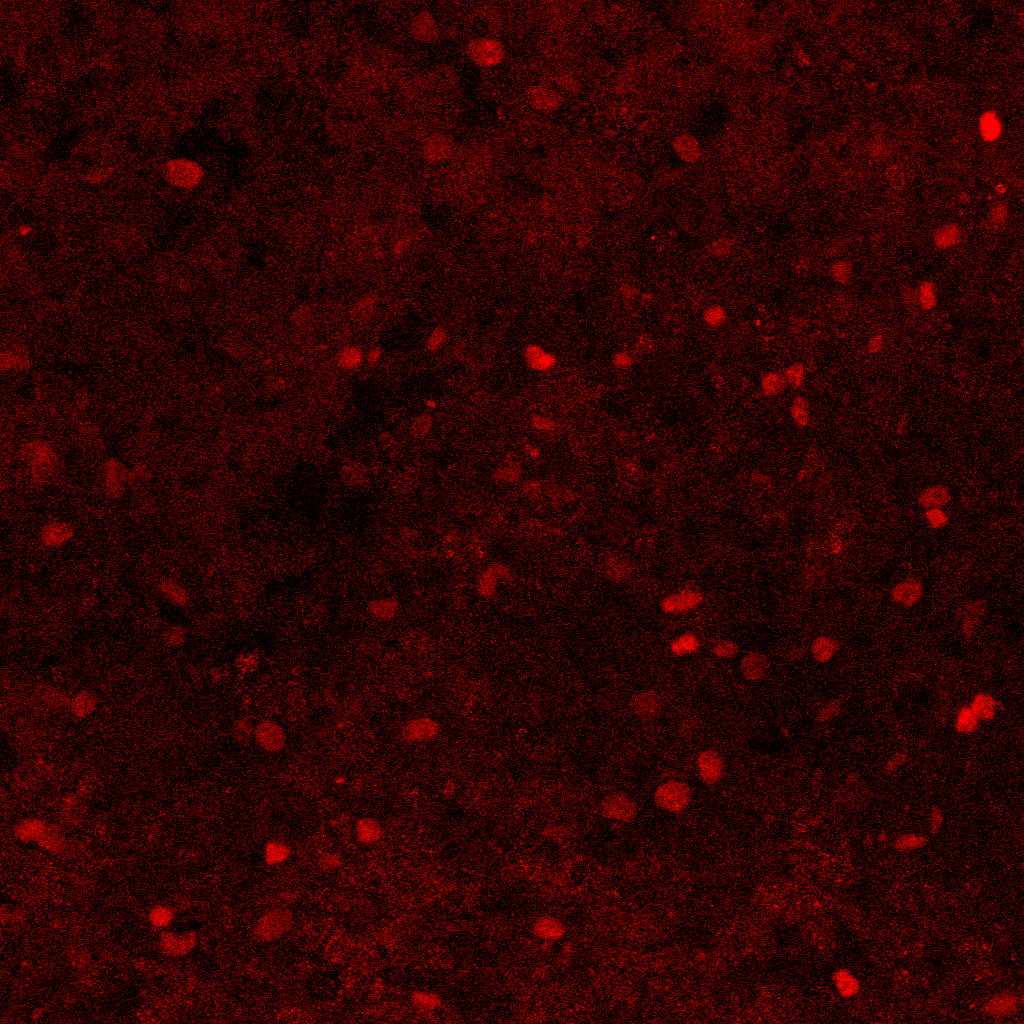

Supplement: Supplementary file 8 — Source Data for Figure 6 [file EMMM-14-e14759-s006.zip › Fig 6/Fig 6-images/NKX2.2-Ki67/C3-MAX_SEMA 7 D NKX 594 KI67 647 MOG 555 GFP.lif - 910b NKX 594 KI67 647 MOG 555 GFP001.tif]

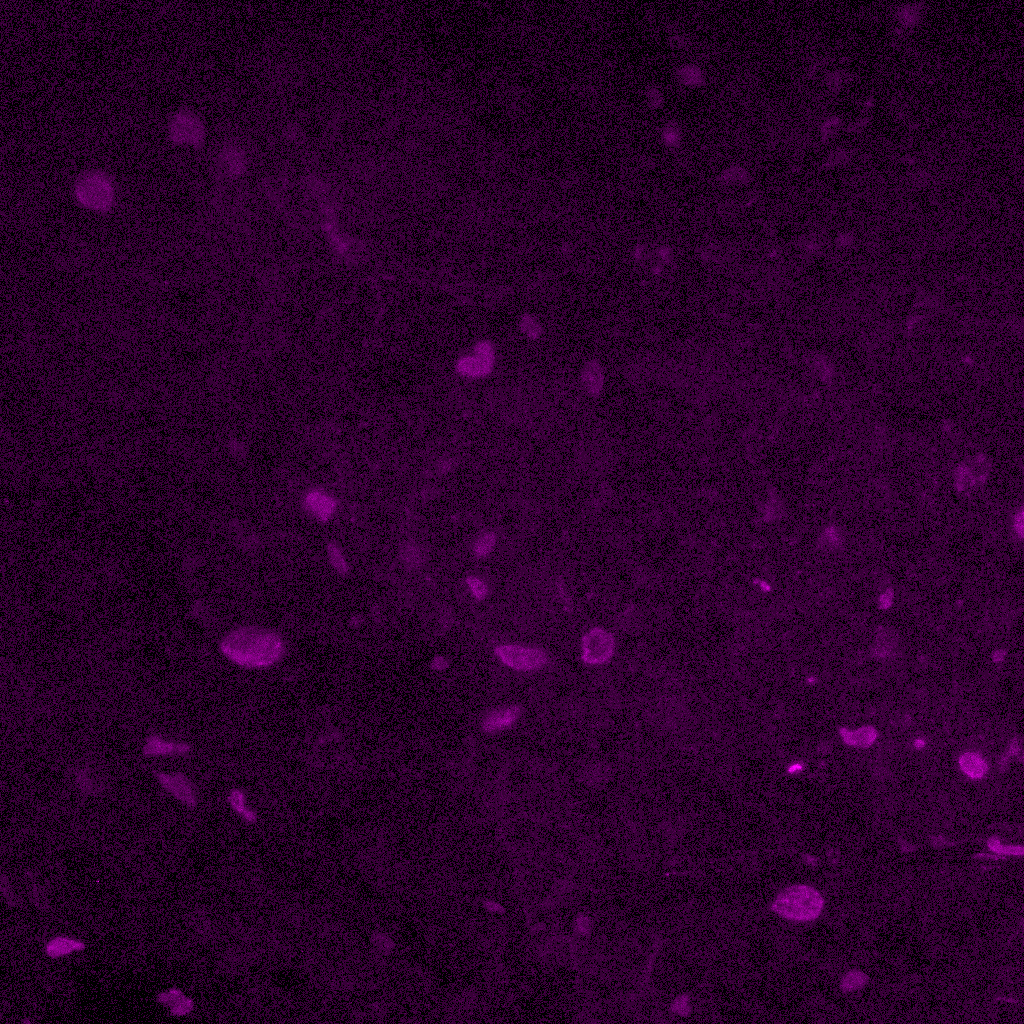

Supplement: Supplementary file 8 — Source Data for Figure 6 [file EMMM-14-e14759-s006.zip › Fig 6/Fig 6-images/NKX2.2-Ki67/C4-MAX_SEMA 7 D NKX 594 KI67 647 MOG 555 GFP.lif - 907 NKX 594 KI67 647 MOG 555 GFP001.tif]

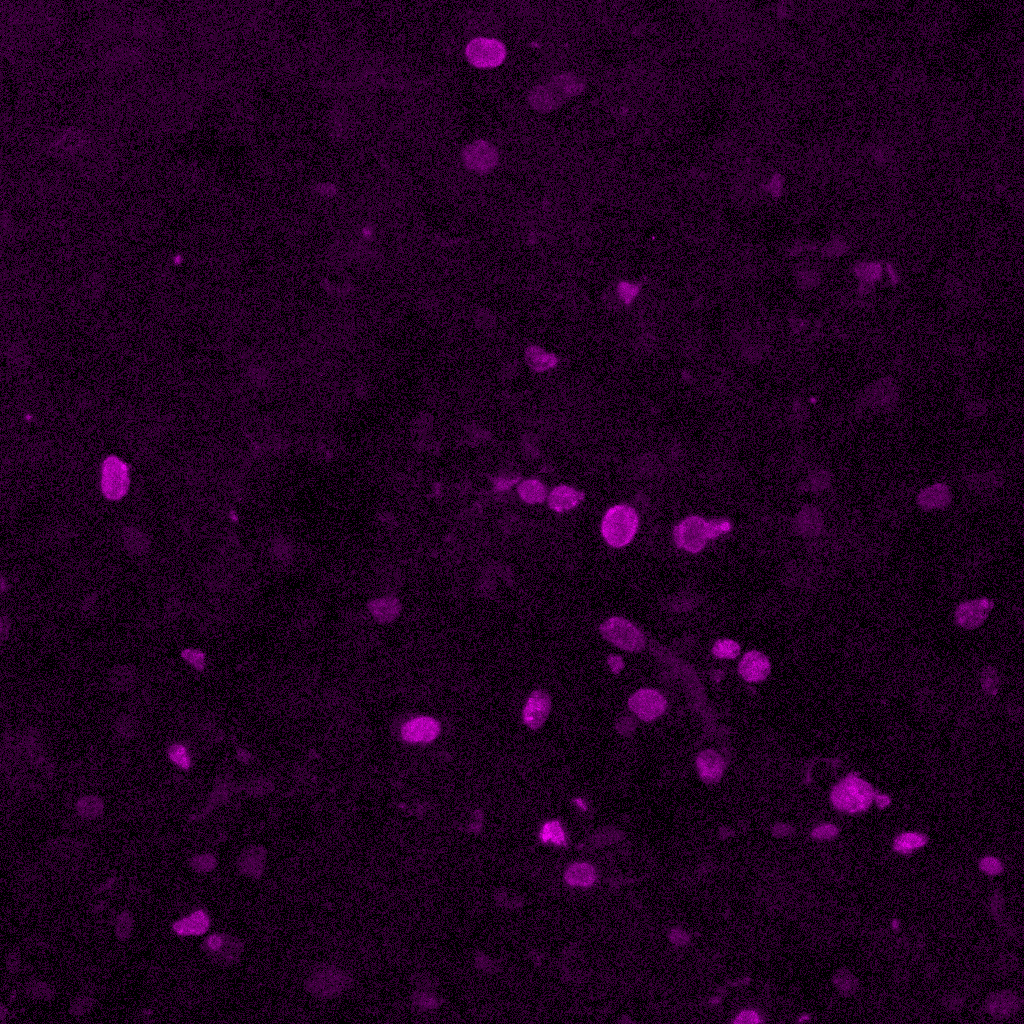

Supplement: Supplementary file 8 — Source Data for Figure 6 [file EMMM-14-e14759-s006.zip › Fig 6/Fig 6-images/NKX2.2-Ki67/C4-MAX_SEMA 7 D NKX 594 KI67 647 MOG 555 GFP.lif - 910b NKX 594 KI67 647 MOG 555 GFP001.tif]

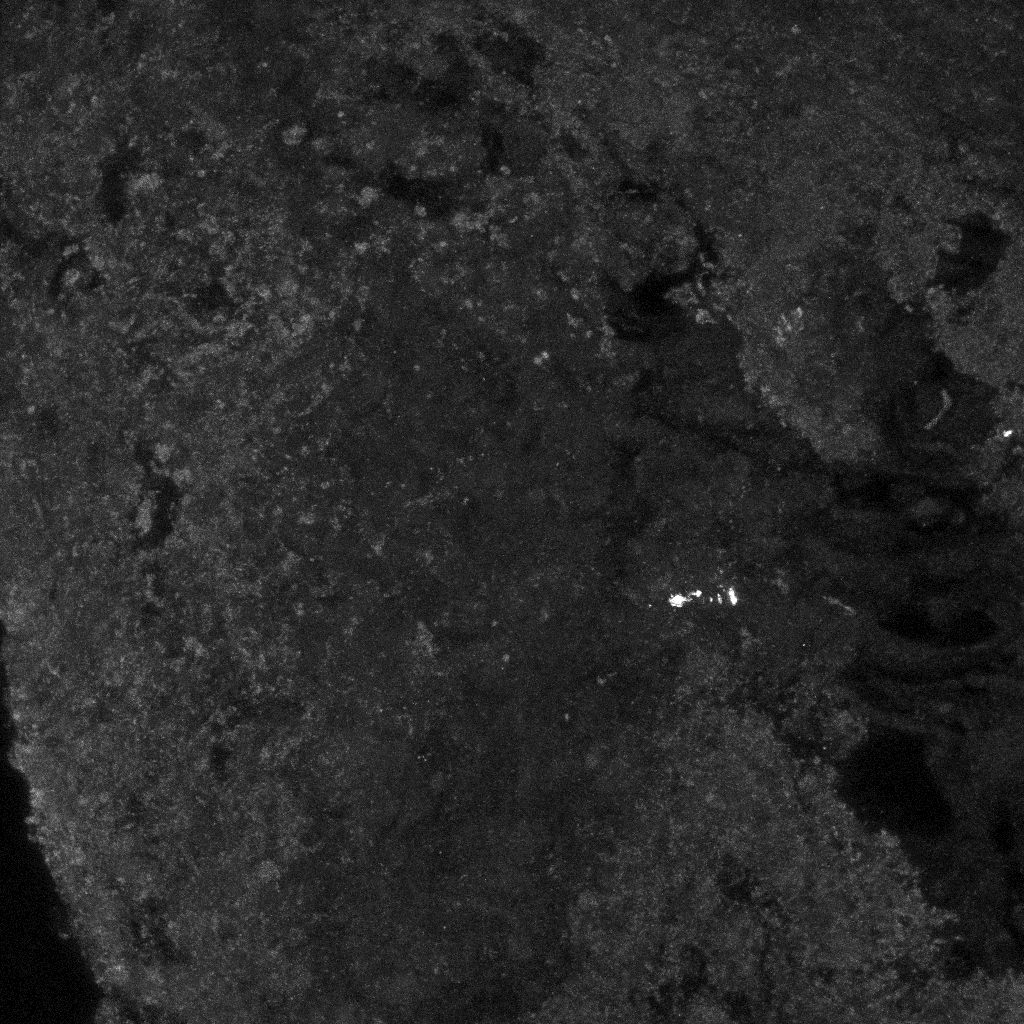

Supplement: Supplementary file 8 — Source Data for Figure 6 [file EMMM-14-e14759-s006.zip › Fig 6/Fig 6-images/Olig2/mbp-MAX_Sema Olig2.lif - 908 1-2.tif]

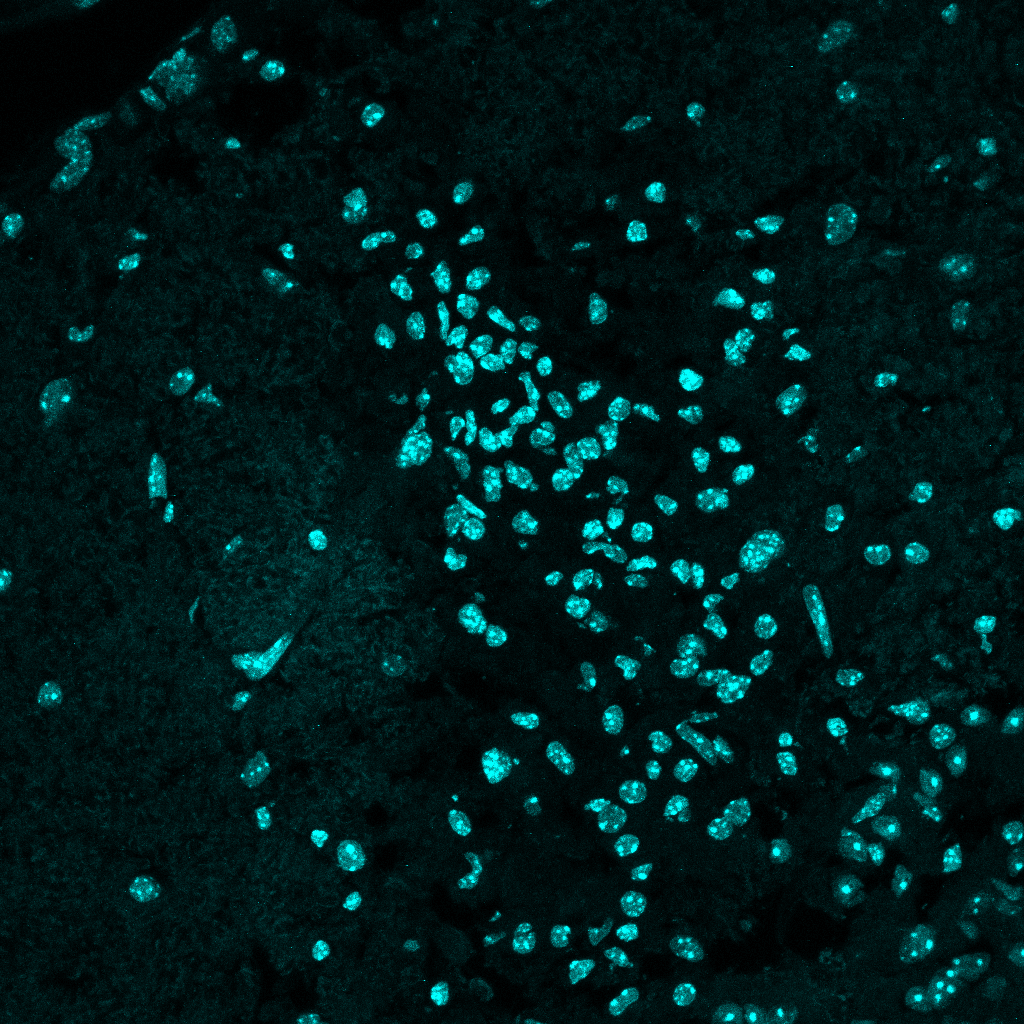

Supplement: Supplementary file 8 — Source Data for Figure 6 [file EMMM-14-e14759-s006.zip › Fig 6/Fig 6-images/PDGFRalpha/DAPI 897 2-2 PALPHA 555 MBP GFP002.tif]

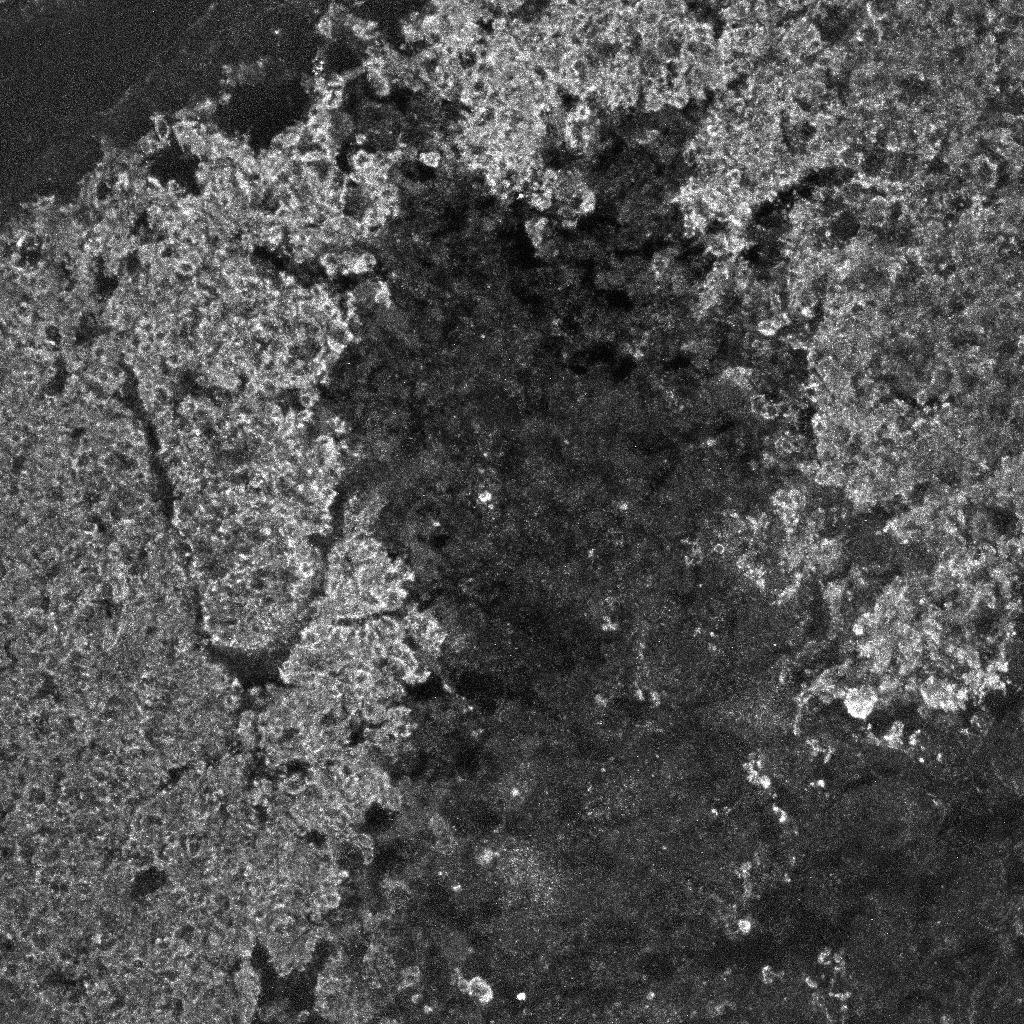

Supplement: Supplementary file 8 — Source Data for Figure 6 [file EMMM-14-e14759-s006.zip › Fig 6/Fig 6-images/PDGFRalpha/mbp 897 2-2 PALPHA 555 MBP GFP002.tif]

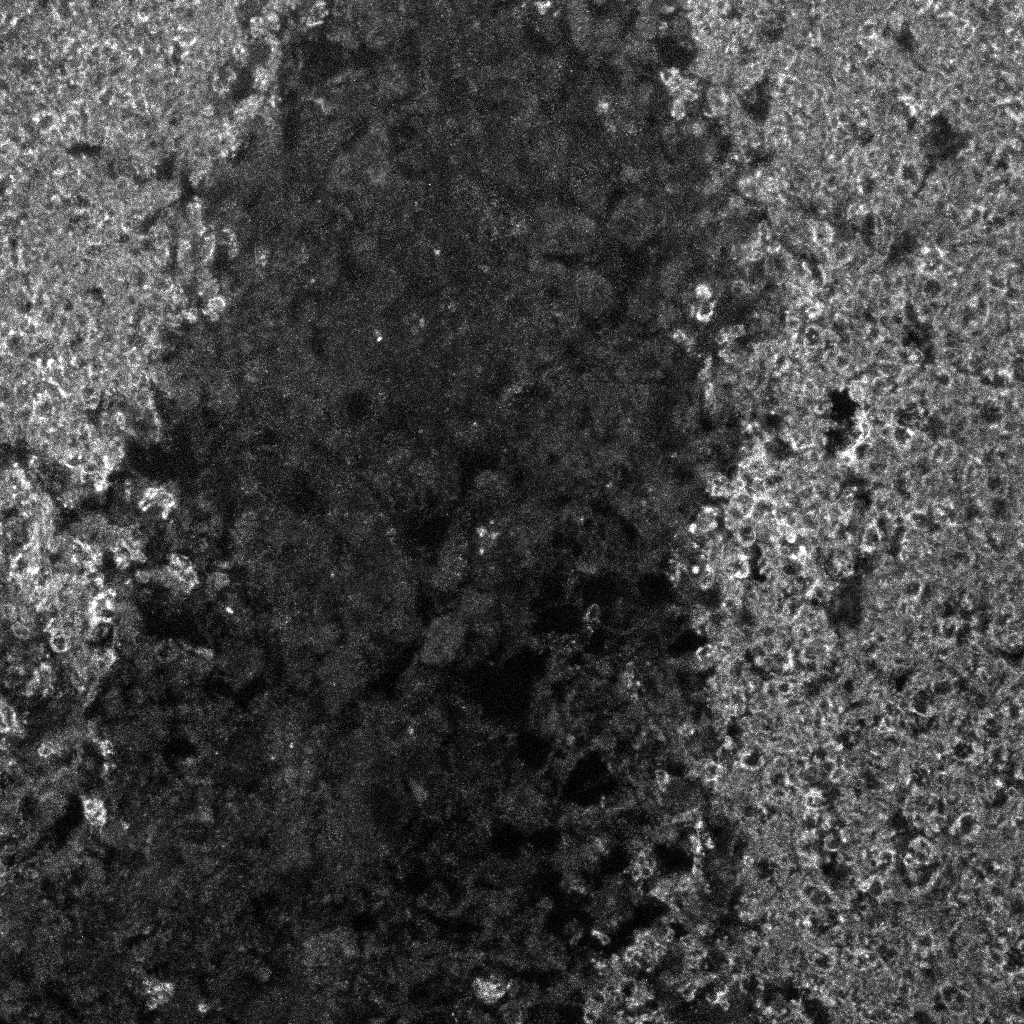

Supplement: Supplementary file 8 — Source Data for Figure 6 [file EMMM-14-e14759-s006.zip › Fig 6/Fig 6-images/PDGFRalpha/MBP 901 3-LAST PALPHA 555 MBP GFP001.tif]

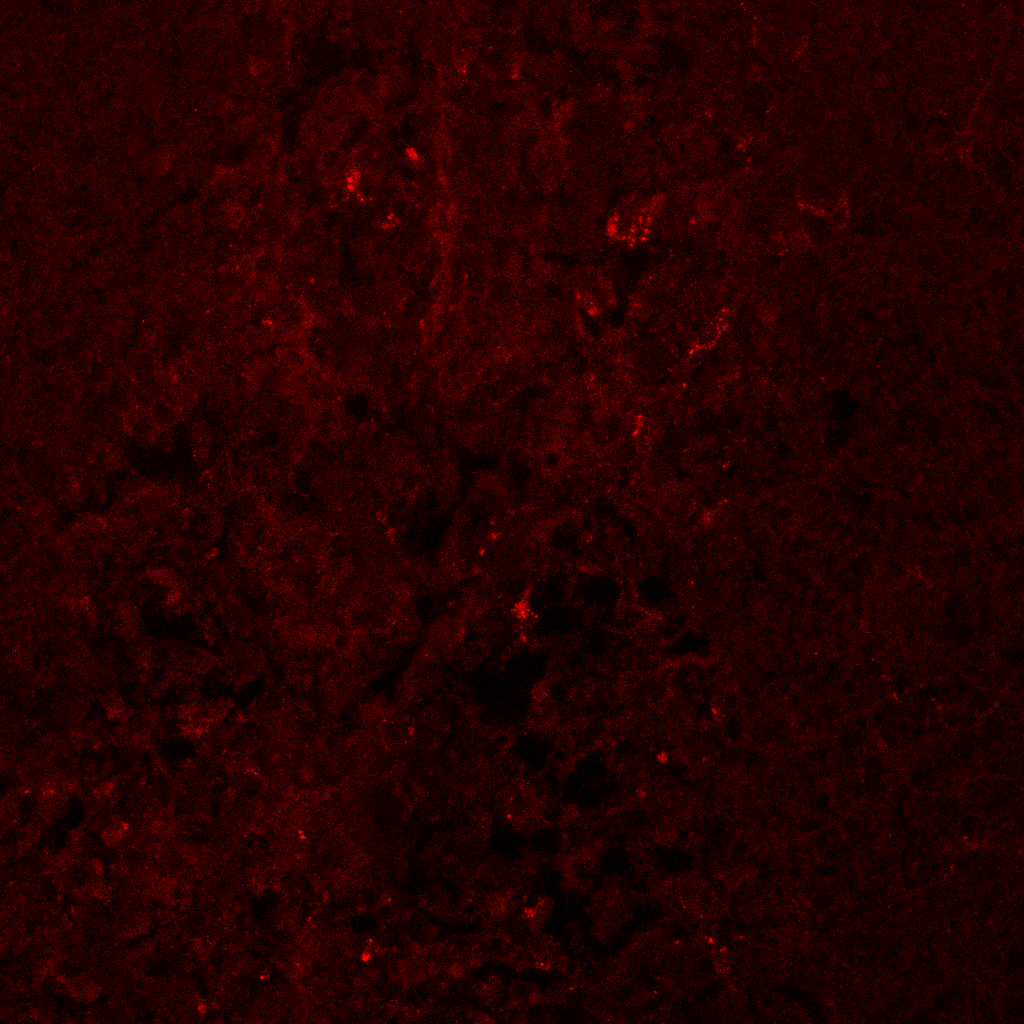

Supplement: Supplementary file 8 — Source Data for Figure 6 [file EMMM-14-e14759-s006.zip › Fig 6/Fig 6-images/PDGFRalpha/palpha - 901 3-LAST PALPHA 555 MBP GFP001.tif]

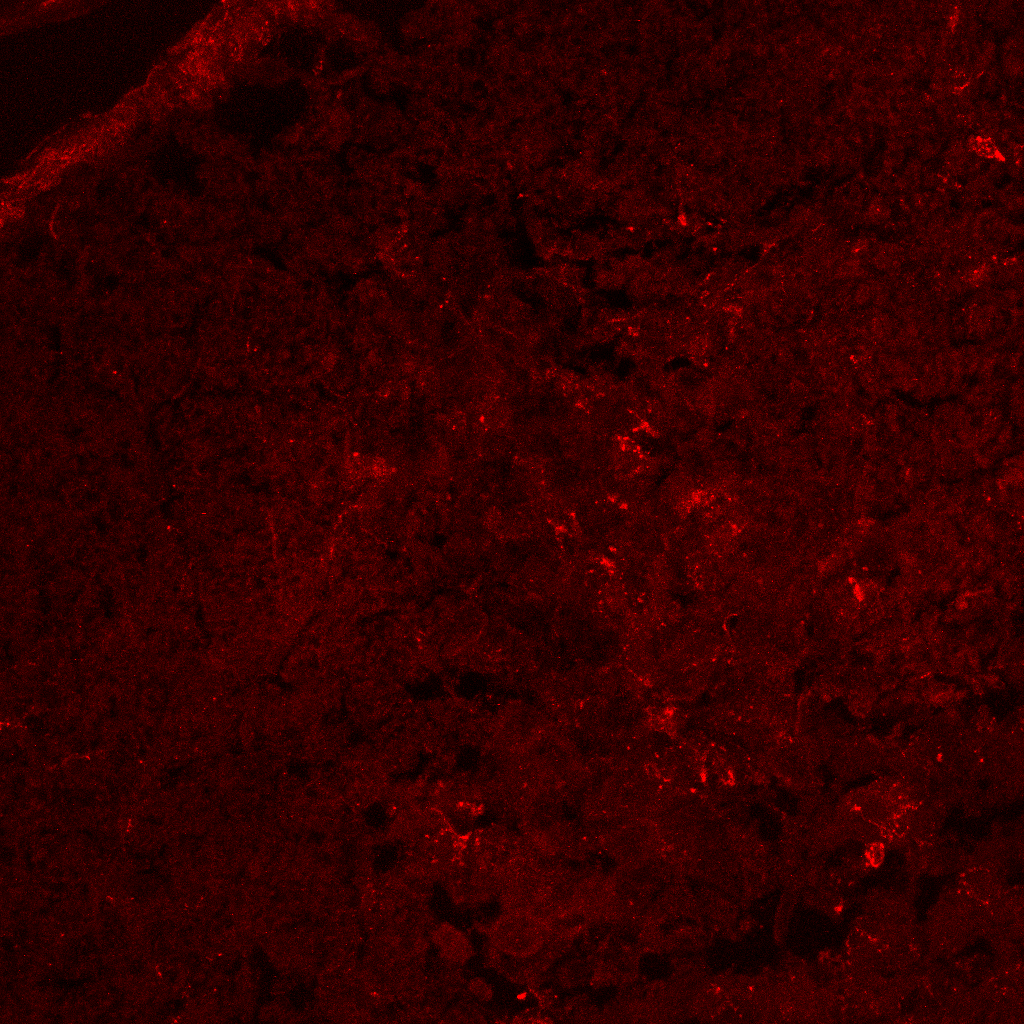

Supplement: Supplementary file 8 — Source Data for Figure 6 [file EMMM-14-e14759-s006.zip › Fig 6/Fig 6-images/PDGFRalpha/palpha-897 2-2 PALPHA 555 MBP GFP002.tif]

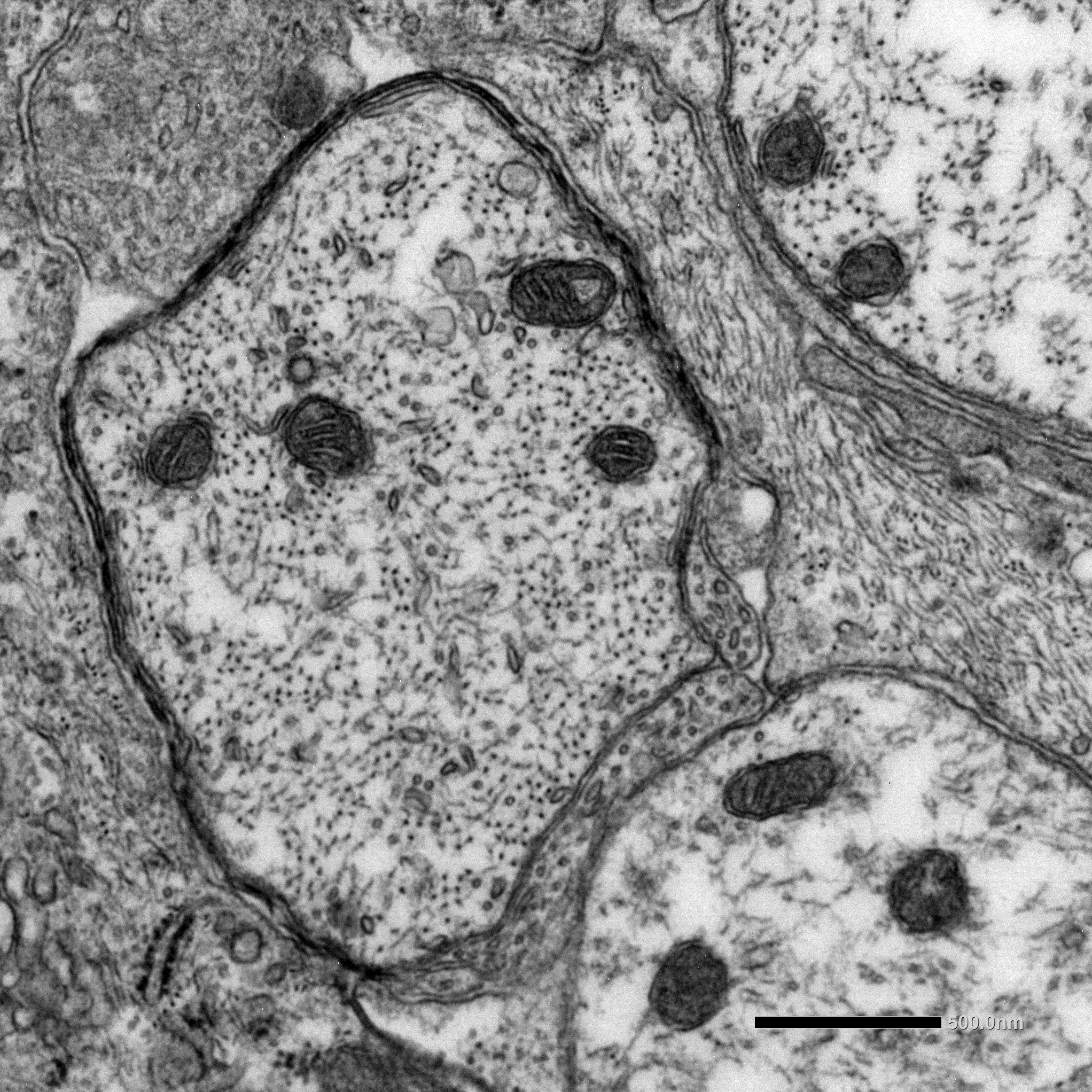

Supplement: Supplementary file 9 — Source Data for Figure 7 [file EMMM-14-e14759-s001.zip › Fig 7/Fig 7-images/26 feb a4-004.bmp]

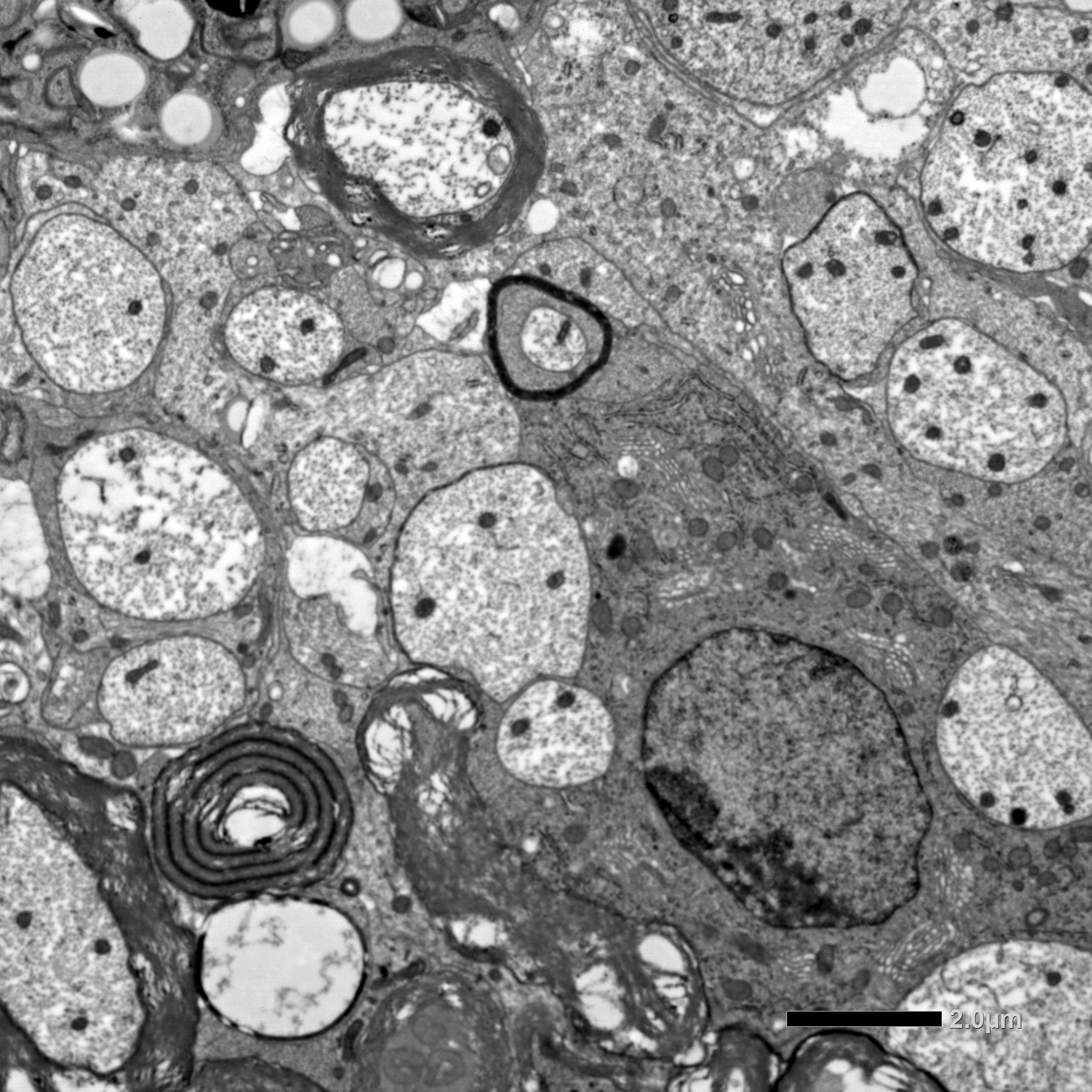

Supplement: Supplementary file 9 — Source Data for Figure 7 [file EMMM-14-e14759-s001.zip › Fig 7/Fig 7-images/26 feb a4-005 .bmp]

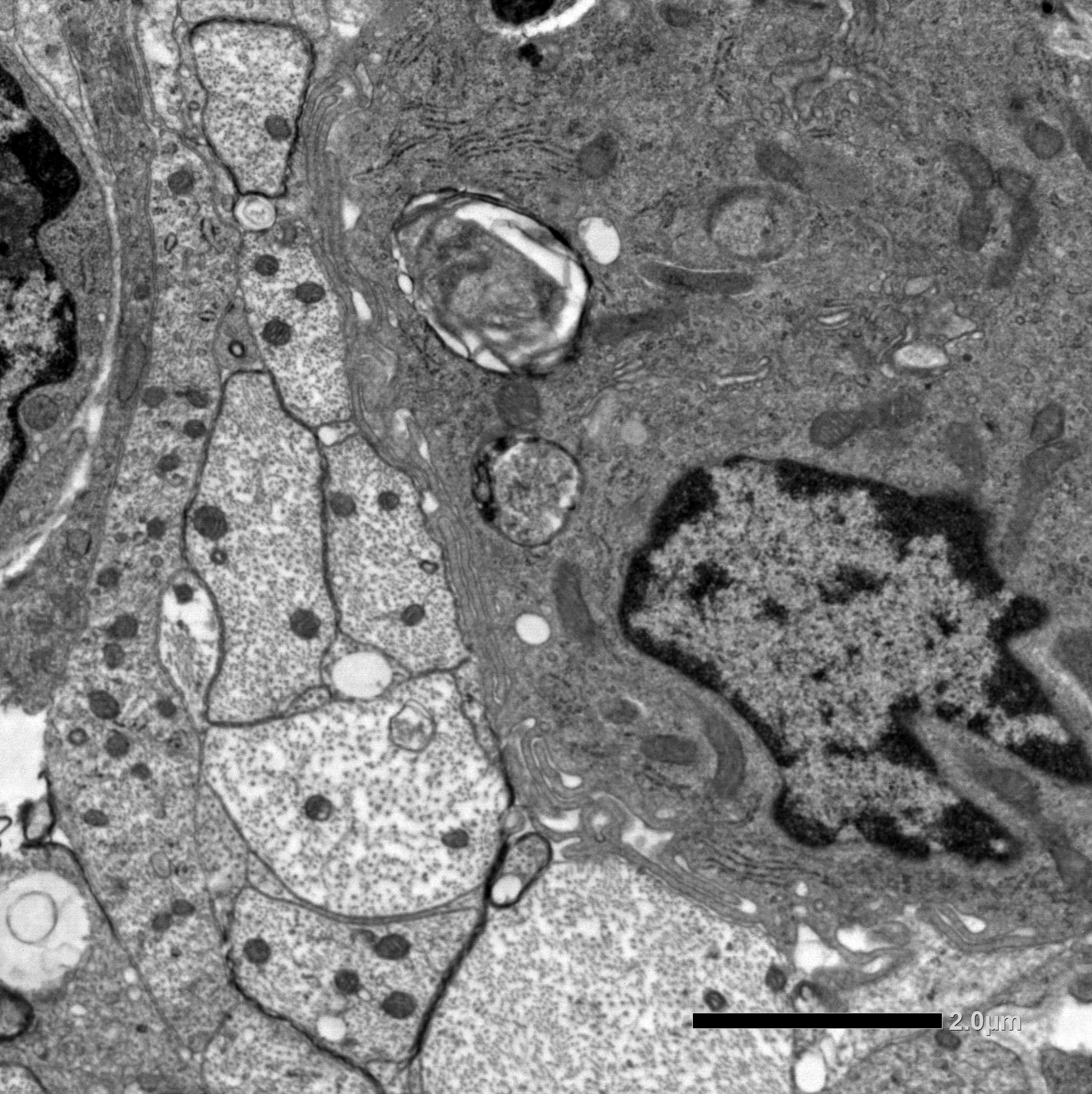

Supplement: Supplementary file 9 — Source Data for Figure 7 [file EMMM-14-e14759-s001.zip › Fig 7/Fig 7-images/5Mar_c-2-034_SA-MAG_X3000 (2).bmp]

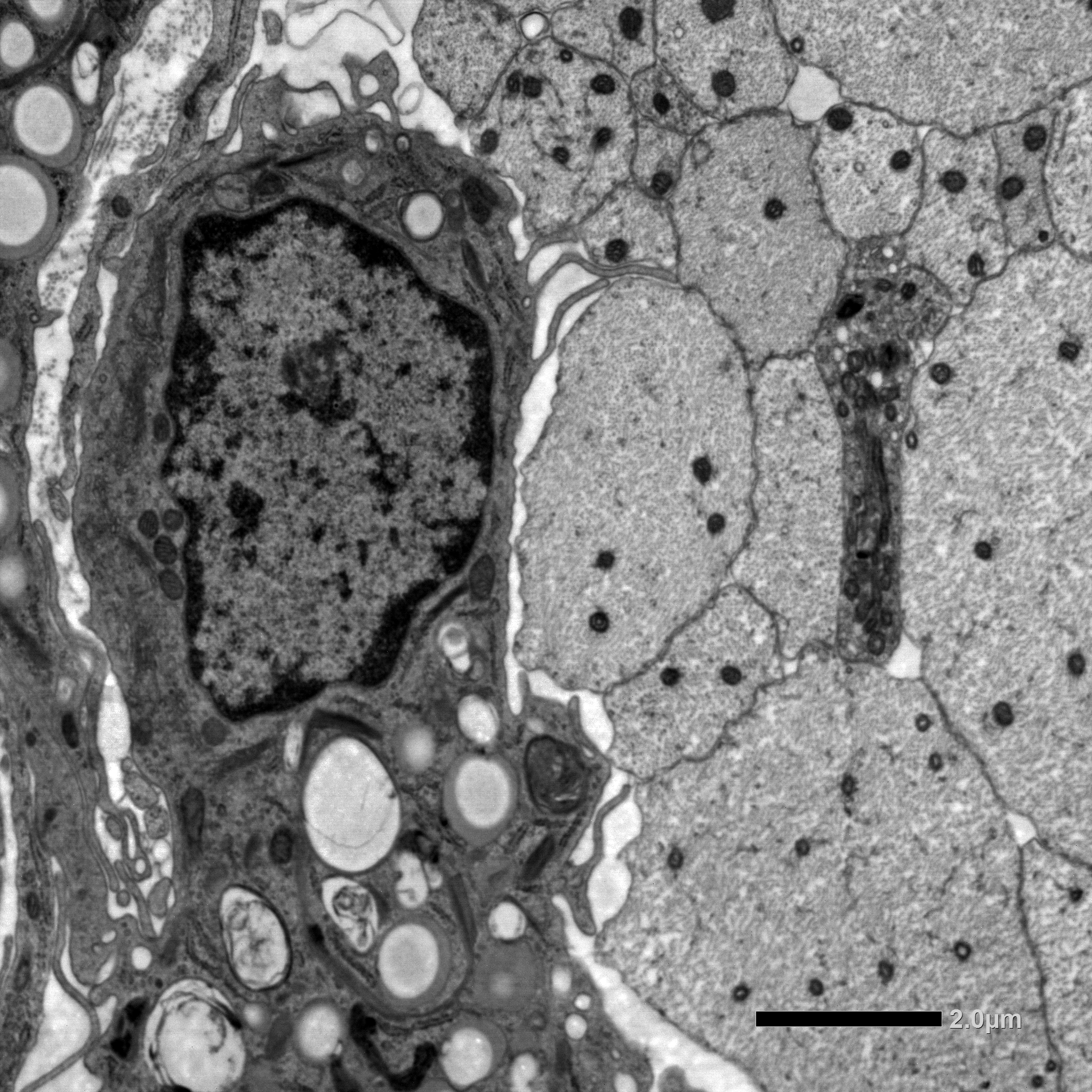

Supplement: Supplementary file 9 — Source Data for Figure 7 [file EMMM-14-e14759-s001.zip › Fig 7/Fig 7-images/5Mar_d-3-034_SA-MAG_X3000 .bmp]

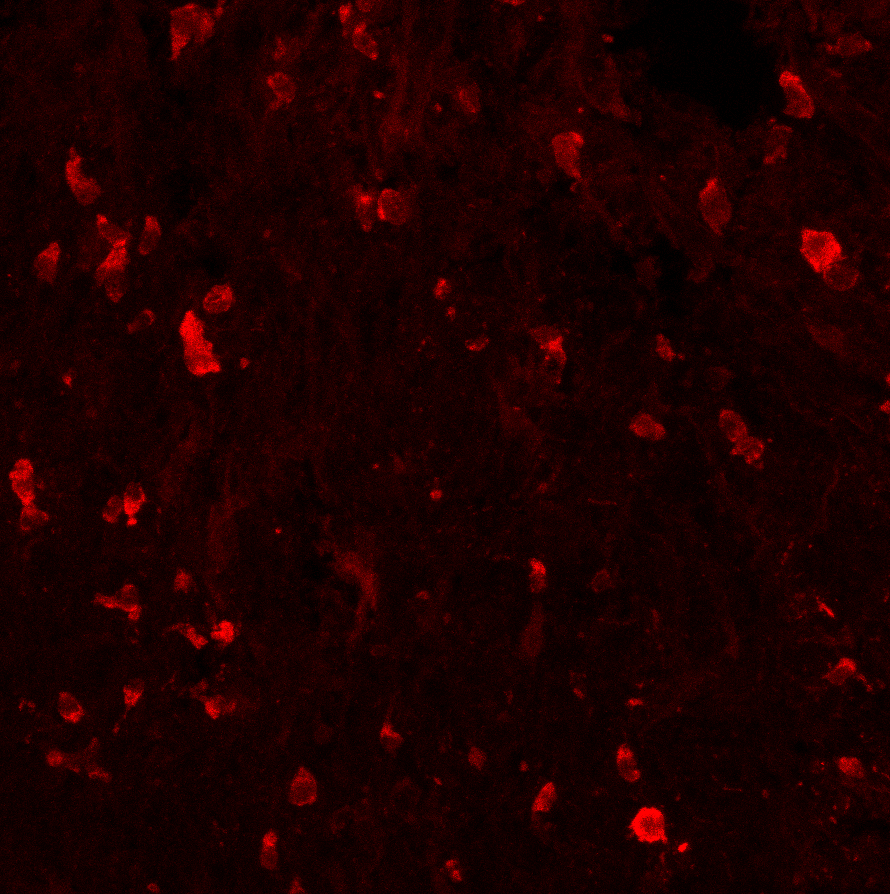

Supplement: Supplementary file 9 — Source Data for Figure 7 [file EMMM-14-e14759-s001.zip › Fig 7/Fig 7-images/APC 304 gfp488 apc 594 olig2 647 mbp 546001-1.tif]

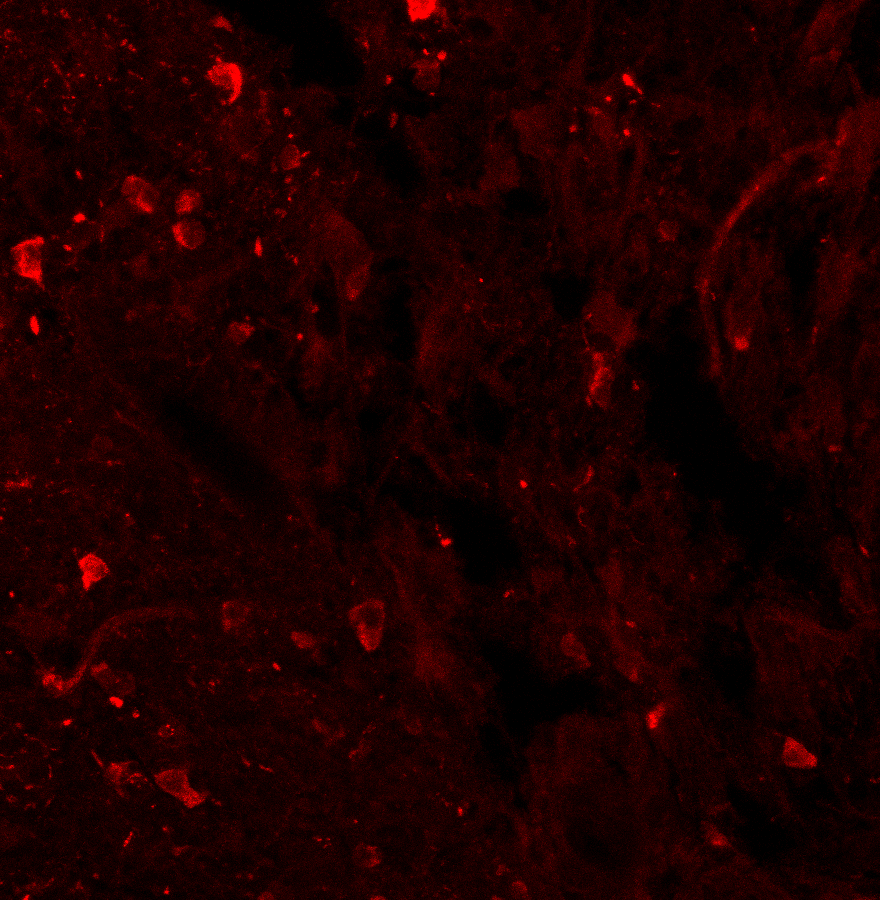

Supplement: Supplementary file 9 — Source Data for Figure 7 [file EMMM-14-e14759-s001.zip › Fig 7/Fig 7-images/APC 313 gfp488 apc 594 olig2 647 mbp 546002-2.tif]
