# Supplementary material for: Genetically modified macrophages accelerate myelin repair
Source: EMBO Mol Med. 2022 Jul 13;14(8):e14759. doi: 10.15252/emmm.202114759 (PMC9358396; doi:10.15252/emmm.202114759)
Supplement: Supplementary file 10 [file EMMM-14-e14759-s005.pdf]

EMBO Molecular Medicine

GENETICALLY-MODIFIED MACROPHAGES ACCELERATE MYELIN REPAIR

Marie-Stephane Aigrot, Clara Barthelemy, Sarah Moyon, Gaelle Dufayet-Chaffaud, Leire Izagirre-Urizar, Beatrix Gillet-Legrand, Satoru Tada, Laura Bayón-Cordero, Juan-Carlos Chara, Carlos Matute, Nathalie Cartier, Catherine Lubetzki, and Vanja Tepavcevic
DOI: 10.15252/emmm.202114759

Corresponding author(s): Vanja Tepavcevic (vanja.tepavcevic@gmail.com) , Catherine Lubetzki (catherine.lubetzki@aphp.fr), Nathalie Cartier (ncartier@askbio.com)

Review Timeline:

Submission Date:	22nd Jun 21
Editorial Decision:	21st Jul 21
Revision Received:	12th Apr 22
Editorial Decision:	9th May 22
Revision Received:	1st Jun 22
Accepted:	2nd Jun 22

Editor: Zeljko Durdevic

Transaction Report:

(Note: With the exception of the correction of typographical or spelling errors that could be a source of ambiguity, letters and reports are not edited. Depending on transfer agreements, referee reports obtained elsewhere may or may not be included in this compilation. Referee reports are anonymous unless the Referee chooses to sign their reports.)

1st Editorial Decision **21st Jul 2021**

21st Jul 2021

Dear Dr. Tepavcevic,

Thank you for the submission of your manuscript to EMBO Molecular Medicine. We have now received feedback from the three reviewers who agreed to evaluate your manuscript. As you will see from the reports below, the referees acknowledge the interest of the study but also raise important critique that should be addressed in a major revision. Focus of the revision should be on repeating the experiments with increased n numbers and elaboration on the clinical applicability of the approach. After our cross-commenting session it became clear that additional animal model is not required.

Further consideration of a revision that addresses reviewers' concerns in full will entail a second round of review. EMBO Molecular Medicine encourages a single round of revision only and therefore, acceptance or rejection of the manuscript will depend on the completeness of your responses included in the next, final version of the manuscript. For this reason, and to save you from any frustrations in the end, I would strongly advise against returning an incomplete revision.

We would welcome the submission of a revised version within three months for further consideration. However, we realize that the current situation is exceptional on the account of the COVID-19/SARS-CoV-2 pandemic. Please let us know if you require longer to complete the revision.

I look forward to receiving your revised manuscript.

Yours sincerely,

Zeljko Durdevic

***** Reviewer's comments *****

Referee #1 (Remarks for Author):

Tepavcevic et al provide data from an experimental system aimed at showing that Sema3F, a factor shown previously to enhance recruitment of OPCs, can be delivered in a clinically applicable manner to sites of demyelination in the CNS and promote remyelination. The approach is to systemically infuse monocytes that have been genetically-modified to overexpress Sema3F. The experimental paradigm involved deriving monocytes from Sema3F-lentiviral vector transduced HSCs (Sema3/GFPP vs GFP only control). Supernatants from these cells are shown to contain Sema3 and stimulate OPC migration (including from middle age animals) in a Neuropilin 2 (Nrp2, Sema3F receptor)-dependent fashion. They then assessed the OPC and remyelination response in an LPC toxin induced spinal cord lesion in chimeric animals with re-constituted SEMa-3 expressing monocytes.

One applauds the approach of seeking a clinically applicable therapy to promote remyelination. The study does provide a proof of principle that systemic myeloid cells arriving at lesion sites can deliver the molecules. The authors' use of middle age animals models clinical disease age.
A conceptual issue is that this approach would be clinically difficult to apply as it would require having individuals with chimeric hematologic states created in anticipation of lesions subsequently occurring. How would this approach apply to progressive/chronic MS?
Comments from the authors as to how they foresee clinical translation and what would be the optimal experimental model (LPC is an acute insult) would be appreciated.

Is there any information as to whether overexpression of Sema 3F alters the functional properties of monocytes within the immune system or interaction with body organs other than the CNS?
The authors have previously found that "Sema3F is detected in early MS lesions, it is not expressed in chronically demyelinated MS lesions". Would this reflect the polarization state of myeloid cells? Is Sema3F expression in the transfected macrophages altered dependent on their state of polarization?

A main experimental issue to consider is how robust is the enhanced remyelination effect?

With regard to the specific experimental studies:
Fig 1 ~47% of macrophages in LPC lesion in chimeric mice were GFP+ - the conclusion is that "both resident and blood-derived macrophages are recruited early to LPC lesions" - are the terms "resident macrophages" and "microglia" interchangeable?

Fig 2 - Migration with supernatants was 2-fold higher in Sema3F-GFP versus GFP (Fig. 2E), which was similar to the increase in migration induced by a recombinant Sema3F at 500ng/mL (2.2 fold compared to control). - is there a dose response for the Sema3F effect? - would greater or lesser transfection have been more effective?
SemaF induces ~a 2-fold increase in OPC migration- is this the most robust effect observed when compared with any other agents?

Fig 3 - immunohistochemical analyses of Sema3F expression in LPC lesions at 4 dpl (Fig. 3C-E) and 7 dpl showed a significant decrease in Sema3F- expressing cells in lesions induced in middle-aged and old (18 month-old) mice at 4 dpl (onset of OPC recruitment), as compared to young mice (Fig. 3F), which was partially due to a decrease in microglial/macrophage Sema3F expression (Fig. 3G). - Do macrophages from older mice migrate as well as younger mice - was this tested in vitro?

Fig 4 - there is rather low (22%) transfection efficiency in the Sema3F-GFP chimeric animals - would this limit how robust the functional effects would be?

Could B cell lineage cells be a significant source of Sema 3F - what relative levels do they secrete?

Fig 5 - there is a paucity of B cells in the LPC lesions - is this different from what is seen in MS lesions?

Fig 6 - related to in vitro questions, can one determine whether the increase in recruited OPCs is the maximum effect that one could achieve with the current Sema3F levels achieved using the available chimeric macrophages?

Fig 7 - shows the results of the initial remyelination process as measured by the proportion of axons surrounded by thin myelin sheaths (EM study). It is somewhat difficult to assess how robust the effect is in the SemaF group (10-30 % of axons have ensheathment with one "outlier"). Is the eventual remyelination similar or different between groups.
How robust is the Sema3F effect (or macrophage supernatant) effect if added to nanofiber assays?

Can one judge the extent of clearance of myelin debris in this model?

This group has transgenic mouse lines with a PDGF R promoter regulated reporter - could OPCs responses be more readily tracked in these mice rather than by immunostaining with conventional antibodies?

Referee #2 (Remarks for Author):

This paper builds on previous work from this group showing that semaphorins regulate oligodendrocyte precursor migration into demyelinating lesions. Here, they test the hypothesis that engineering infiltrating macrophages to express the attractant sema3F will enhance regeneration by stimulating the ingress of oligodendrocyte precursor cells. The methodology used; viral transfection of haematopoietic stem cells which were then transplanted into conditioned mice so as to reconstitute the myeloid lineage is sound. The results are clearly presented and show that the manipulation does increase precursor entry into the lesions. This is an important next step in our understanding of the potential therapeutic applications of semaphoris in MS and also an elegant proof of principle as to the use of macrophages as a delivery tool for bioactive molecules into demyelinating lesions.

There are some questions that should be considered to improve the manuscript

More data on the timing of the GFP positive macrophages into demyelinating lesions as required. The three day and seven day time points were apparently examined, but no data are shown. This data needs to be presented for these and later time points when remyelination is largely complete. At each time point, distinction between macrophages and microglia would be very helpful

In the lesions, where exactly are the GFP positive macrophages? To what extent do they infiltrate the demyelinated axons within these lesions, or are they confined to the perivascular regions? More sophisticated three-dimensional reconstructions would address this and would most helpful

The spatial distribution of remyelination following the entry of sema3F/GFP positive cells needs to be examined. Figure 6 shows rosettes of precursors around DAPI clusters. Does this show that the infiltrating precursors are in fact homing to clusters of cells expressing semaF rather than being distributed throughout the lesion, and does this in turn means that the remyelination is patchy rather than spread through the lesion?

Regarding the quantification of remyelination by both light and electron microscopy, nowhere did I see a statement that the observer doing the count was blinded as to whether or not the sections were from control or experimental animals. A clear statement confirming blinding is required, as are details of the methods used to ensure blinding. This is particularly important if remyelination is not uniformly spread, and details as to how the areas for analysis chosen should therefore also be included.

In the discussion, some consideration of how this might translate into therapy is required. In particular, will the genetically manipulated macrophages enter a chronic lesion in patients with progressive disease-the likely targets of such treatments? The experimental paradigm here mimics acute lesions very well, but these are not the patients that will require targeting in the clinic

Referee #3 (Remarks for Author):

In their manuscript Tepavčević and colleagues study the effect of hematopoietic stem cells (HSC) overexpressing Sema3F on remyelination in the lysolecithin model. They demonstrate that the Sema3F overexpressing HSC differentiate into monocytes and enter the lysolcithin lesions. Supernatants from Sema3F overexpressing HSC increase OPC migration in vitro. Mice injected with Sema3F overexpressing HSC display an increased number of Sema3F positive intralesional monocytes, increased numbers of oligodendrocytes and an accelerated remyelination.
This is a continuation of former work by the same group. It is an interesting study, the experiments are mostly well performed

and the manuscript is well written and easy to follow. However, I have some technical as well as some conceptual concerns.

1. Only relatively few animals per time point have been analyzed for the in vivo experiments. The significant increase in remyelination at 14 days appears to depend more or less on two animals (Fig. 7G). Therefore, one would like to see a confirmation of the results in a second independent experiment or in another demyelinating animal model.

2. The authors chose an animal model which remyelinates well and they observed a somewhat accelerated differentiation of oligodendrocytes as well as remyelination. However, especially in chronic MS lesions the tissue environment may inhibit remyelination. There is no animal model that mimics chronically demyelinated MS lesions; however the authors should consider choosing a more "challenging" animal model, e.g. lysolecithin lesions in older animals.

3. My third point is related to this. Remyelination works very well in the typical demyelinating animal models and is only somewhat slowed down in older animals. However, the situation in MS is more complex. Results from imaging studies suggest that the lesions of many patients display significant remyelination within the first five to six months after lesion formation. So, these patients are most likely not the ones which would benefit from a remyelination promoting therapy. However, there appears to be a subset of lesions which show limited remyelination even in initial lesion stages. More recent studies from initial MS lesions stages suggest that not the lack of mature oligodendrocytes per se contributes to limited remyelination but instead an impaired myelin sheath formation (at least at this lesion stage). At later disease / lesion stages, when remyelination is less frequently observed, the blood brain barrier is relatively closed and it will be questionable whether peripheral cells have a chance to access the CNS parenchyma. Therefore, the authors should discuss more critically which patients at which disease stage and which lesion type might benefit from such a remyelination promoting strategy. They should also discuss critically the limitations of their animal model.

Reviewer's comments

Referee#1:

Tepavcevic et al provide data from an experimental system aimed at showing that Sema3F, a factor shown previously to enhance recruitment of OPCs, can be delivered in a clinically applicable manner to sites of demyelination in the CNS and promote remyelination. The approach is to systemically infuse monocytes that have been genetically-modified to overexpress Sema3F. The experimental paradigm involved deriving monocytes from Sema3F-lentiviral vector transduced HSCs (Sema3/GFPP vs GFP only control). Supernatants from these cells are shown to contain Sema3 and stimulate OPC migration (including from middle age animals) in a Neuropilin 2 (Nrp2, Sema3F receptor)-dependent fashion. They then assessed the OPC and remyelination response in an LPC toxin induced spinal cord lesion in chimeric animals with re-constituted SEMa-3 expressing monocytes.

One applauds the approach of seeking a clinically applicable therapy to promote remyelination. The study does provide a proof of principle that systemic myeloid cells arriving at lesion sites can deliver the molecules. The authors' use of middle age animals models clinical disease age. We sincerely thank the reviewer for acknowledging our efforts to identify a clinically-applicable strategy to enhance oligodendroglial repopulation of MS lesions and remyelination. We hope that our proof of concept will inspire the design of novel advanced therapies for remyelination. A conceptual issue is that this approach would be clinically difficult to apply as it would require having individuals with chimeric hematologic states created in anticipation of lesions subsequently occurring. How would this approach apply to progressive/chronic MS?

One of the main challenges of MS therapeutics is to prevent chronic neurological deficit that arises because of axonal loss in lesions, which is in part due to failure of remyelination after consecutive demyelinating episodes. Such episodes, as we published before (Tepavcevic et al, 2014, doi: 10.1002/ana.24201), mainly lead to differentiation of local OPCs, but not proliferation which, at least in a subset of MS lesions, significantly decreases OPC numbers thus leading to progressive failure of remyelination and axonal degeneration. We think our strategy to increase OPC recruitment should be applied as early as possible (e.g. during relapsing remitting disease) to attract OPCs to the lesion and ensure that sufficient OPCs will be available during consecutive demyelinating phases to provide quick remyelination and thus neuroprotection. This type of therapy, just like any remyelination-promoting therapy, would have to be proposed either during the relapsing-remitting phase or in early progressive phase (i.e. before occurence of severe neuronal damage). Our hope is that neuroprotection achieved in this way will prevent/significantly delay transition to the progressive phase (treatment at relapsing-remitting stage) or reduce/stop further deficit development (treatment at early progressive stage) . While we have used chimeric mice to provide a proof of concept, we highlight that alternative strategies based on genetically-modified macrophages are possible. This is now clarified throughout the Discussion, particularly on pages 35-36.

Comments from the authors as to how they foresee clinical translation and what would be the optimal experimental model (LPC is an acute insult) would be appreciated.

We think that our work is providing an important proof of concept that genetically modified myeloid cells can increase myelin regeneration. This strategy could be implemented in several ways. In patients with aggressive disease, in which aHSCT is already applied, our protocol could be used, thus allowing not only to halt the destructive inflammatory process, but also to stimulate regeneration in damaged areas. In patients with milder forms of the disease, systemic injections of genetically-modified monocytes could be used. The use of lysolecithin model, in which demyelination is induced by a toxin (independent from immune response) and clearly separated in time from the remyelinating phase, has allowed us to clearly establish the regenerative effects of transgene-carrying macrophages (instead of interference with induction of demyelination). This would not have been possible with other models such as cuprizone or EAE, the induction of the latter being completely dependent on the immune system. Moreover, the timing of OPC recruitment, proliferation, and differentiation is very well defined in the lysolecithin model, which greatly facilitates studies of remyelination.

One possibility to test our strategy using transplantation of genetically-modified HSCs in a more translational model of MS would be to induce EAE using adoptive transfer of pathogenic T cells, isolated from non-chimeric donor mice. In this way, pathology induction would not be affected by the transgene in the host immune system, so that the effect specifically on neuroprotection/myelin regeneration could be evaluated. This next step however requires technical expertise in the induction of adoptive transfer EAE.

We think that it is crucial to test the applicability of systemic injections of genetically-modified monocytes (no preconditioning required), which should be done first in the lysolecithin model, and then in cuprizone and EAE (rat EAE and Biozzi mouse EAE being those with a significant demyelinating component). If these experiments show promising results, the best pre-clinical model to test systemic injections of genetically-modified myeloid cells would be marmoset EAE, which probably is the closest animal model of MS. These ideas are now summarized in the Discussion, page 36.

Is there any information as to whether overexpression of Sema 3F alters the functional properties of monocytes within the immune system or interaction with body organs other than the CNS?

This is an important question. We had already shown that Sema3F overexpressing monocytes successfully reach demyelinating lesions (Fig.5) and we now also show they clear the myelin debris equally well as control cells (newly added data, text on page 25, Appendix Fig.S3). In this new version, we have also analyzed GFP cell numbers in the heart, liver, lung, and the spleen. As mentioned in the text, we detected some GFP cells in the brain, similarly to previously published data using busulfan pre-conditioning, but the numbers were low and similar in GFP and Sema 3F mice. As expected, we have found numerous GFP+ cells in the spleen (secondary lymphoid organ that also contains myeloid cells), and in lower numbers in the lungs (contain alveolar and interstitial macrophages, and other immune cells). Numbers of GFP+ cells in these organs in the two groups were mainly dependent on the percentage of chimerism in the blood, as detected using correlation analyses, which suggests that

Sema3F expression did not significantly affect the accumulation of GFP positive cells in these organs (page 23).

It is important to mention that our goal was to provide the proof of concept that blood macrophages are reliable deliverers of therapeutic molecules to demyelinating lesions, for which we used a ubiquitous promoter to drive Sema3F expression. However, in clinical perspective, using advanced gene therapy tools, it may be possible to restrict the transgene expression by using promoters specific to phagocytic macrophages, to reduce/prevent the transgene delivery in healthy, non-lesioned tissues (now mentioned in Discussion, page 36).

The authors have previously found that "Sema3F is detected in early MS lesions, it is not expressed in chronically demyelinated MS lesions". Would this reflect the polarization state of myeloid cells? Is Sema3F expression in the transfected macrophages altered dependent on their state of polarization?

Our data in lysolecithin lesions (Fig. 3G) show that in young mice (efficient remyelination) Sema 3F expression by macrophages is the highest at 3 days post lesion, which is coincident with M1 polarization state that determines OPC proliferation (Miron et al., 2013, doi: 10.1038/nn.3469). In the transduced cells, we are forcing Sema 3F expression under ubiquitous promoter, so these cells will continuously express Sema 3F. The switch from M1 to M2 state has been reported as indispensable for OPC differentiation (Miron et al., 2013). While we have not tested this specific transition in Sema3F-overexpressing macrophages, the OPCs in Sema3F mice successfully differentiate (Fig.7), which, according to the above-mentioned paper, would not have happened if M2 macrophages had been significantly affected.

In chronic MS lesions, lack of Sema 3F expression actually appears associated with low numbers of inflammatory cells in the lesion core as well as with the lack of Sema 3F expression on scar-forming astrocytes (as opposed to reactive astrocytes during early demyelinating phase).

A main experimental issue to consider is how robust is the enhanced remyelination effect?

We have been able to increase OPC recruitment and accelerate the onset of remyelination using genetically-modified macrophages, with a more robust effect on OPC recruitment (fold increase at 7dpl for PDGFRα+ cells, 2.5 fold) than direct injections of Sema 3F-encoding lentiviral vector in the lesion area (1.3 fold increase at 7dpl for PDGFRα+ cells; Piaton et al., doi: 10.1093/brain/awr022). Thus, we think that the main contribution of our current work is the demonstration that this clinically-applicable strategy can result into similar benefits as previously described lentiviral injections of Sema3F, and therefore could be used to express a variety of different molecules of interest to stimulate myelin regeneration/neuroprotection.

With regard to the specific experimental studies:
Fig 1 ~47% of macrophages in LPC lesion in chimeric mice were GFP+ - the conclusion is that "both resident and blood-derived macrophages are recruited early to LPC lesions" - are the terms "resident macrophages" and "microglia" interchangeable?

We thank the Reviewer for pointing this out. In this case, yes, we were referring to both resident macrophages and microglia, we specify this now in the text (page 25).

However, in this new version of the manuscript, we have also specifically analyzed the cells labelled with a microglial marker P2Y12. (Fig.5V-Ñ, text on page 25)

Fig 2 - Migration with supernatants was 2-fold higher in Sema3F-GFP versus GFP (Fig. 2E), which was similar to the increase in migration induced by a recombinant Sema3F at 500ng/mL (2.2 fold compared to control). - is there a dose response for the Sema3F effect? In this manuscript and the previously published one (Piaton al 2011, doi: 10.1093/brain/awr022) we chose 500 ng/mL of the recombinant human Semaphorin 3F because this concentration produces 50% of the optimal binding response to immobilized Nrp2 - would greater or lesser transfection have been more effective?" A ½ dilution of our supernatant led to a similar increase in migration as recombinant Sema3F, which we consider effective. We did not perform serial dilutions as our migration experiments are performed using adult OPCs isolated by FACS from an OPC-reporter line, with limited OPC numbers obtained per experiment (approximately 300000 with young mice, 150000 for middle-aged mice). We perform migration assays in duplicates or triplicates for each condition, which means that it is extremely challenging to increase the numbers of conditions. Therefore, we used the protocol standardized in our previous publication on Sema 3F (Piaton et al., 2011, doi: 10.1093/brain/awr022).

SemaF induces ~a 2-fold increase in OPC migration- is this the most robust effect observed when compared with any other agents?

For example, using the same in vitro migration assay, Bonfanti and colleagues, 2017 (https://doi.org/10.1038/cddis.2017.256) showed that PDGF AA, a known OPC chemoattractant and mitogen, increases adult OPC migration by 1,3 or 1,5 fold, depending on the OPC subpopulation. Thus, we consider that a 2-fold increase in OPC migration in vitro can be considered at least as robust as that of PDGF AA.

Fig 3 - immunohistochemical analyses of Sema3F expression in LPC lesions at 4 dpl (Fig. 3C-E) and 7 dpl showed a significant decrease in Sema3F- expressing cells in lesions induced in middle-aged and old (18 month-old) mice at 4 dpl (onset of OPC recruitment), as compared to young mice (Fig. 3F), which was partially due to a decrease in microglial/macrophage Sema3F expression (Fig. 3G). - Do macrophages from older mice migrate as well as younger mice - was this tested in vitro?

This is an interesting question. We did not perform in vitro tests of macrophage migration, but we did investigate macrophage numbers in lysolecithin lesions in young versus middle-aged versus old mice. Our examination of Iba1+ labelling showed a small, non-significant decrease between young and middle-aged and old mice at 4 days post lesion, and no differences at 7 dpl (see graph below), which suggests that older macrophages show a slight delay in recruitment. This population (Iba1+) however includes both resident microglia/macrophages and monocyte-derived macrophages.

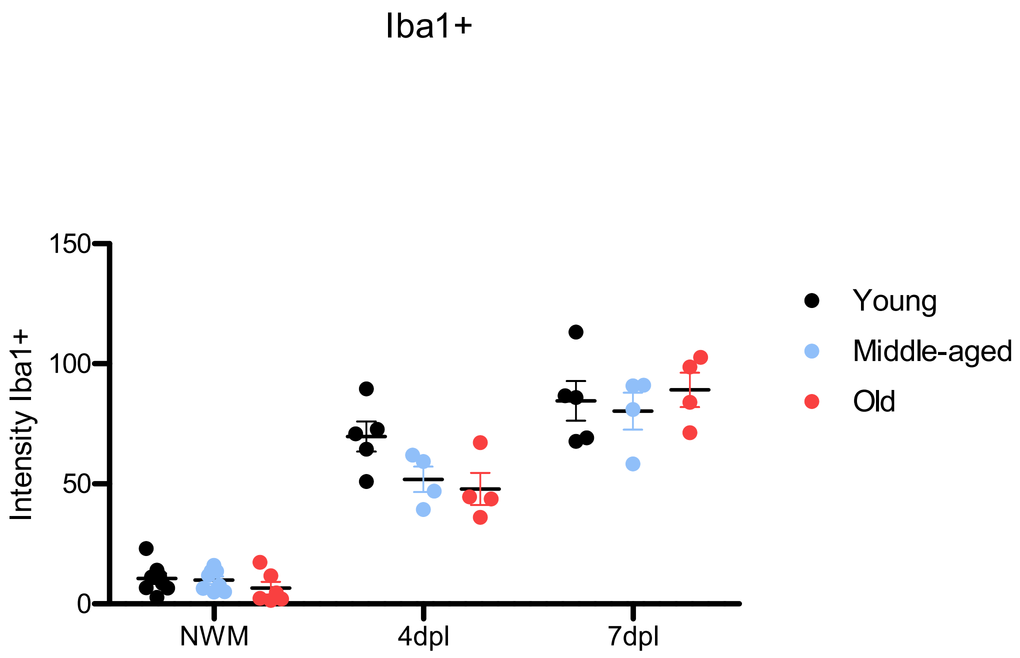

Regarding monocytes, using a parabiosis system in which they could detect blood-derived cells (originating from parabiotic partner blood) using GFP fluorescence, Ruck and colleagues (2012, doi: 10.1016/j.stem.2011.11.019) observed comparable partner-derived monocyte recruitment to demyelinating lesions with young versus old partners, suggesting that impaired macrophage migration was not a key factor underlying aged-induced impairment in remyelination. While this paper suggests that monocyte recruitment to lysolecithin lesions is not significantly affected with age, future studies should clearly investigate both microglia and monocyte recruitment to aged lesions in a non-parabiosis system.

Fig 4 - there is rather low (22%) transfection efficiency in the Sema3F-GFP chimeric animals - would this limit how robust the functional effects would be?

Even this low efficiency should significantly increase Sema3F concentration in the lesions, as this is overexpression. Importantly, this transduction efficiency is sufficient to detect a robust increase in Sema3F protein in the Sema3F-GFP transduced cell supernatant in vitro, which then, increases OPC migration in Boyden chamber assay.

Could B cell lineage cells be a significant source of Sema 3F - what relative levels do they secrete?

We are not aware of publications that show Sema3F expression by B cells. In our Sema3F-chimera lesions, as we show in Fig.5, there are very few B cells, and these are mostly GFP-negative so we do not think they contribute significantly to intrealesional Sema3F.

Fig 5 - there is a paucity of B cells in the LPC lesions - is this different from what is seen in MS lesions?

Yes, it is very different, only occasional B cells are recruited to LPC lesions as demyelination in this model is induced by LPC toxicity for membranes rather than being mediated by the immune system. In MS, a subset of lesions shows extensive infiltration of B cells. (Lucchinetti et al, 2000 doi: 10.1002/1531-8249(200006)47:6<707::aid-ana3>3.0.co;2-q)

Fig 6 - related to in vitro questions, can one determine whether the increase in recruited OPCs is the maximum effect that one could achieve with the current Sema3F levels achieved using the available chimeric macrophages?

While we cannot deduce whether we achieved a maximal increase in OPC recruitment, we can compare it to our previous work (Piaton et al., 2011, doi: 10.1093/brain/awr022) in which Sema3F-expressing lentivirus was directly injected in the lesion. At 7 dpl, the increase observed was of approximately 1.3 fold, which is less than what we observed in the current study (2.5 fold), probably because the number of intralesional transduced cells in our chimeric system is higher. Moreover, transgene expression after direct lentiviral intralesional injections is not immediate, while transduced monocytes/macrophages are already overexpressing Sema3F at the time of infiltration.

Fig 7 - shows the results of the initial remyelination process as measured by the proportion of axons surrounded by thin myelin sheaths (EM study). It is somewhat difficult to assess how robust the effect is in the SemaF group (10-30 % of axons have ensheathment with one "outlier"). Is the eventual remyelination similar or different between groups.

For the revision of this manuscript, we performed an experiment in which we sacrificed the mice at 18 dpl (to examine advancement in remyelination but prior to completion of this process, as it is much more difficult to assess acceleration once remyelination approaches completion in the control animals). We initially used a few animals to check whether we could see an advancement in remyelination in Sema3F chimeras at 18dpl, and we indeed confirmed it was the case (see image below, red arrows point to remyelinated axons).

first set 18 dpl

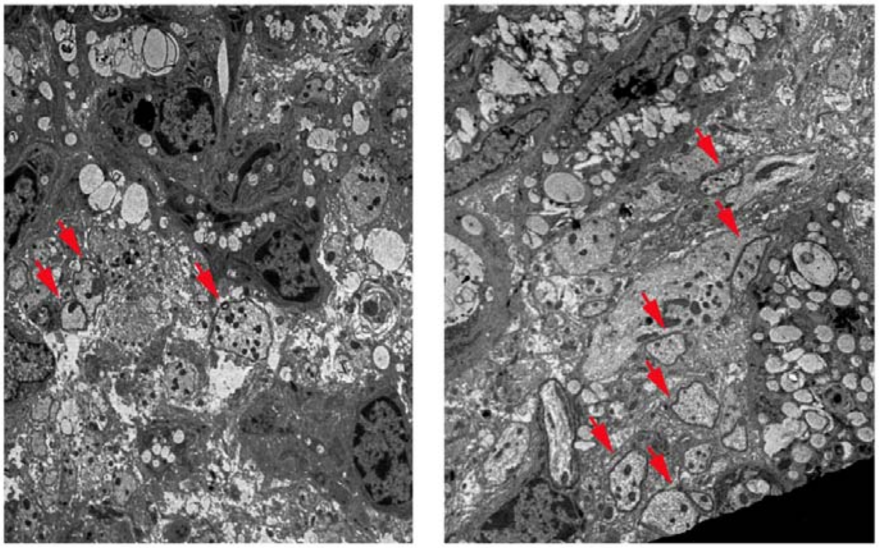

GFP mouse (15% total rem) Sema3F mouse (33% total rem)

In this preliminary experiment, we only had a few animals (other animals from this set were used to increase the "n" of our immunohistochemical analyses) so we could not perform statistical analyses of remyelination at 18 dpl. We then generated a new set of chimeras, all of which we perfused at 18 dpl and processed and included in resin-for electron microscopy. Unfortunately, the analyses of semi-thin sections revealed spongy vacuolation in the spinal cord that affected mostly gray matter but also the white matter. This pathology has been previously described as indicative of neurodegeneration and attributed to immunosuppression followed by viral reactivation/new infection. This was the first time it happened in our mice. The vacuolation in the white matter that was extensive in some mice prevented us from analyzing remyelination. Besides, these pathological mice are unsuitable for evaluation of remyelination. Thus, despite the extensive amount of work, material, and large number of animals invested in this long experiment (lesions are performed 10 weeks after HSC transplant), we were not able to complete the analyses and answer this question. Because repeating this experiment would have implied many more months, as we would have also had to order a new batch of viral vectors, we were not able to do it for this revision, unfortunately.

However, it is worth noting that after reinforcing our experiment at 14 dpl with a few additional mice (Fig.7), we observe a clear difference in the percentage of remyelinated axons. It is important to highlight that this accelerated remyelination could be very significant in MS by reducing the chances of axonal degeneration in inflammatory conditions.

How robust is the Sema3F effect (or macrophage supernatant) effect if added to nanofiber assays?

While nanofiber assay is very useful to evaluate the influence of extrinsic factors on OPC differentiation, it is not adapted to evaluate OPC recruitment.

Can one judge the extent of clearance of myelin debris in this model?

We have now performed these additional analyses and do not observe significant differences between the groups in the amounts of myelin debris (Appendix new Fig S3 and new text on page 25).

This group has transgenic mouse lines with a PDGF R promoter regulated reporter - could OPCs responses be more readily tracked in these mice rather than by immunostaining with conventional antibodies?

It is true that this line would be a valuable tool for imaging studies of OPC recruitment for example. However, because in the reporter line OPCs are GFP+, this means it would be incompatible with our system in which we use GFP tag to detect transduced hematopoietic cells in the lesions. It is also much easier to ensure sufficient mice of required age by purchasing wt mice for chimera experiments that require large numbers of animals.

Referee#2:

This paper builds on previous work from this group showing that semaphorins regulate

oligodendrocyte precursor migration into demyelinating lesions. Here, they test the hypothesis that engineering infiltrating macrophages to express the attractant sema3F will enhance regeneration by stimulating the ingress of oligodendrocyte precursor cells. The methodology used; viral transfection of haematopoietic stem cells which were then transplanted into conditioned mice so as to reconstitute the myeloid lineage is sound. The results are clearly presented and show that the manipulation does increase precursor entry into the lesions. This is an important next step in our understanding of the potential therapeutic applications of semaphoris in MS and also an elegant proof of principle as to the use of macrophages as a delivery tool for bioactive molecules into demyelinating lesions.

We thank the reviewer for this evaluation.

There are some questions that should be considered to improve the manuscript

More data on the timing of the GFP positive macrophages into demyelinating lesions as required. The three day and seven day time points were apparently examined, but no data are shown. This data needs to be presented for these and later time points when remyelination is largely complete. At each time point, distinction between macrophages and microglia would be very helpful.

Using actin-GFP chimeras, we established that transgene-carrying transplanted cells are recruited to the lesions as early as 3 dpl, and that similar numbers are present in the lesions at 7 dpl. Using lentivirally-transduced (GFP and Sema3F-GFP) chimeras we then studied the numbers of infiltrating GFP+ cells in both conditions at 7, 10, and 60 dpl (at 60 dpl remyelination is complete), as well as the dynamics of GFP+ macrophages, T cells and B cells, and these results are presented in Figure 5. In this new version of the manuscript, we have increased the number of animals analyzed at 7 and 10 dpl, and we have also included the analyses of microglia, using P2Y12 antibody as a marker (Fig. 5,text page 25). These new analyses clearly show that numbers of GFP cells (transduced infiltrating cells) and Iba1+ cells (both blood-born macrophages and resident macrophages/microglia) decrease with time, while microglial numbers remain fairly stable in both groups, suggesting that Sema3F mice show unchanged microglial reaction. It is, however, possible that some microglial cells downregulate P2Y12, as described for several specific microglial markers (P2Y12, TMEM119) in some pathological settings. We did not perform the 3dpl time point because it is too early to evaluate the recruitment and, as explained below (response to Referee 3-comment 1), generation of large numbers of chimeric mice is difficult in practical terms. We now also provide evidence that, at 7dpl, myelin debris clearance is comparable between the groups (text on page 25, Appendix Fig. S3), which indirectly shows that macrophage infiltration is not significantly altered.

In the lesions, where exactly are the GFP positive macrophages? To what extent do they infiltrate the demyelinated axons within these lesions, or are they confined to the perivascular regions? More sophisticated three-dimensional reconstructions would address this and would most helpful.

Macrophage infiltration in the lysolecithin model has been extensively characterized before, and these cells colonize the entire lesion and clean myelin debris, and do not remain confined to perivascular regions. We clearly observe this using electron microscopy as we detect high numbers of macrophages outside the perivascular space. As shown in Fig 5, the GFP+ cells are distributed throughout the area of

demyelination, and as mentioned in the manuscript, some infiltrate peri-lesional areas. Interestingly, it has been reported that busulfan pre-conditioning actually increases the colonization of normal, non-lesioned parenchyma by peripheral cells (now cited in the manuscript).

The spatial distribution of remyelination following the entry of sema3F/GFP positive cells needs to be examined. Figure 6 shows rosettes of precursors around DAPI clusters. Does this show that the infiltrating precursors are in fact homing to clusters of cells expressing semaF rather than being distributed throughout the lesion, and does this in turn means that the remyelination is patchy rather than spread through the lesion?

In this new version of the manuscript, we are providing images of Nkx2.2 labelled-OPCs and GFP+ cells that indeed show OPC presence next to Sema3F-expressing GFP+ macrophages (Fig. 6I; text on page 28). Moreover, at the early remyelination time point analyzed (14 dpl), remyelination did seem patchy, and we could observe remyelinated axons in the close proximity of macrophages (see image below, now included in Fig.7). However, because this was TEM analyses, we could not analyze whether these were GFP+ macrophages. What we think is most important, is that the recruitment of transduced macrophages to the lesions will specifically increase Sema3F levels in these areas compared to the perilesional tissue and thus mobilize perilesional OPCs towards the areas of demyelination.

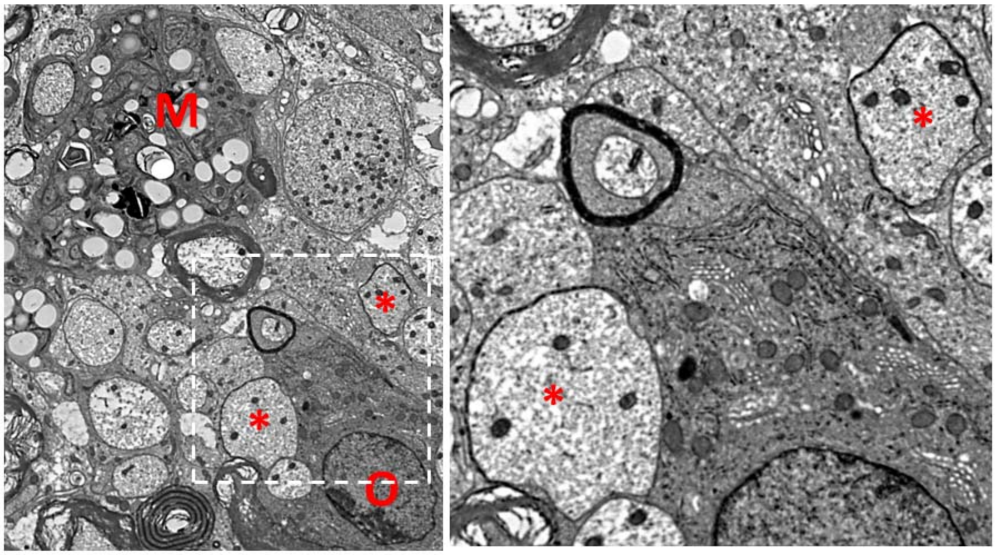

M-Myelin debris-filled macrophage O-Reactive oligodendroglial cell

* Axons undergoing remyelination

Regarding the quantification of remyelination by both light and electron microscopy, nowhere did I see a statement that the observer doing the count was blinded as to whether or not the sections were from control or experimental animals. A clear statement confirming blinding is required, as are details of the methods used to ensure

blinding. This is particularly important if remyelination is not uniformly spread, and details as to how the areas for analysis chosen should therefore also be included.

Thank you pointing this out, we have now included this statement in the Methods section. We have also highlighted that, for the analyses, EM images are taken to cover the whole demyelinated area (which is easily identified because non-demyelinated tissue has a very thick myelin), and remyelinated and demyelinated axons are counted. We did not focus the analyses on a specific region within the lesions. This is a standard method we use to quantify remyelination.

In the discussion, some consideration of how this might translate into therapy is required. In particular, will the genetically manipulated macrophages enter a chronic lesion in patients with progressive disease-the likely targets of such treatments? The experimental paradigm here mimics acute lesions very well, but these are not the patients that will require targeting in the clinic

This is a very important point that we have also addressed in response to Reviewer 1. What we would like to highlight (it has been reinforced in the current version of the manuscript) is that we think that stimulating remyelination as early as possible is crucial to prevent cumulative axonal loss and the transition to progressive disease. As explained throughout discussion, successive episodes of demyelination/remyelination, typical in MS, likely exhaust the lesional OPC pool, particularly with age, when OPC recruitment efficiency decreases. Moreover, the first page of the Discussion illustrates why quick arrival of OPCs is required for successful remyelination. Thus, by using macrophages as vehicles of Sema3F, we hope to prevent the chronicity of demyelination by ensuring quick arrival of progenitor cells to demyelinated axons (while axons are still permissive to remyelination), which will increase the chances of remyelination thus reducing axonal loss and the development of progression. This pro-regenerative therapy should be associated to an immunomodulatory component targeting auto-reactive lymphocytes to limit further relapses. Ideally, patients should be treated during active disease (relapsing phase, which would be the OPTIMAL phase for any pro-remyelinating treatments, while axons are still viable) to prevent the chronicity of demyelination and consequently, axonal loss and progression, which is what current immunomodulatory treatments are not achieving.

Regarding progressive disease, we think that important benefits could be achieved in terms of neuroprotection. Even though extensive bouts of white matter inflammation are mostly absent in this phase, neuropathological studies suggest that lesions in progressive patients, particularly chronic active lesions, still contain macrophages, so we think that genetically-modified macrophages should reach these lesions. Importantly, it has been demonstrated that busulfan, the pre-conditioning treatment, enhances long-term brain engraftment of peripheral macrophages in the absence of blood-brain barrier breakdown, which explains the therapeutic benefits obtained in patients with leukodystrophies, in which grafted, genetically modified blood-born cells contribute to permanent expression of therapeutic proteins in the brain. Permanent engraftment of transgene-carrying macrophages in patients with MS could be very useful, not only to overexpress pro-remyelinating molecules, but also neurotrophic factors that may prevent general white and gray matter degeneration seen at progressive stages. This is reiterated throughout Discussion and particularly reinforced in newly added text on pages 35-36. In addition, as mentioned above, we want to highlight that our work is only the first step in investigating myeloid cell gene therapies for MS, and we think that it represents an important proof of concept to inspire such

novel clinical strategies.

Referee #3 (Remarks for Author):

In their manuscript Tepavčević and colleagues study the effect of hematopoietic stem cells (HSC) overexpressing Sema3F on remyelination in the lysolecithin model. They demonstrate that the Sema3F overexpressing HSC differentiate into monocytes and enter the lysolcithin lesions. Supernatants from Sema3F overexpressing HSC increase OPC migration in vitro. Mice injected with Sema3F overexpressing HSC display an increased number of Sema3F positive intralesional monocytes, increased numbers of oligodendrocytes and an accelerated remyelination.
This is a continuation of former work by the same group. It is an interesting study, the experiments are mostly well performed and the manuscript is well written and easy to follow. We thank the reviewer for this positive evaluation.

However, I have some technical as well as some conceptual concerns.

1. Only relatively few animals per time point have been analyzed for the in vivo experiments. The significant increase in remyelination at 14 days appears to depend more or less on two animals (Fig. 7G). Therefore, one would like to see a confirmation of the results in a second independent experiment or in another demyelinating animal model. This is a valid point. Somewhat low numbers of animals in the initial version of the manuscript were due to the fact that many donor mice and large amounts of lentiviral vectors are required to generate sufficient cells for transplantation. In addition, busulfan treatment is associated with variable amounts of mortality of recipient mice, thus further reducing the final number of chimeras. To prepare this revised version, we have generated several new sets of animals (new, independent experiments) and used these to complete all immunohistochemical analyses of immune cell infiltration and OPC recruitment and differentiation (7 and 10 dpl, Figures 5, 6, and 7). We did not have enough animals, nor enough time (these experiments last for months) to increase the "n" at 60dpl, but at this time point OPC recruitment/differentiation/remyelination are completed which is why we preferred to reinforce the 7 and 10 dpl time-points. Because of increased "n", the statistical difference between GFP and Sema3F groups has now increased. We have also added 1-2 animals per group in the analyses of remyelination at 14 dpl (Figure 7).

As mentioned above in the response to Reviewer 1, comment on Figure 7, we have also performed a separate experiment in which we sacrificed 12 animals (6 per group) at 18 dpl to evaluate the advancement of remyelination (we had previously sacrificed some animals at this time point and confirmed a more advanced remyelination compared to 14dpl, increased in Sema3F mice). However, as mentioned above, the spinal cord tissue of the animals from the new experiment showed generalized spongiform vacuolation that we believe is the result of immunosuppression by busulfan and subsequent viral infection/reactivation of previous infection. Thus, we could not study remyelination in these animals, as explained in detail in the response to reviewer 1, comment 7. As detailed above, the generation of chimeric mice is extremely costly and long, and repeating this experiment would take us at least 6 more months. We do sincerely hope however, that the currently presented experiments with increased "n" will be sufficient to convince the reviewers that our approach is effective in increasing OPC recruitment and accelerating OPC differentiation/remyelination.

Regarding a different model of remyelination, we were very happy that the editorial decision letter (after cross-commenting session with the reviewers) clearly stated that another model was not necessary because, as we mention in the Discussion, models such as EAE or cuprizone are largely incompatible with HSC transplantation for 2 main reasons:

1. Transgene-carrying cells may likely interfere with pathology induction (immune system dependent, particularly in EAE), for which we would not be able to clearly establish a pro-regenerative effect. In the lysolecithin model, this is possible because demyelination is immune-system independent as it is induced by lysolecithin toxicity for membranes. Importantly, the advantage of lysolecithin lesions is that the timing of OPC recruitment, proliferation, differentiation, and remyelination is well defined, which makes it possible to pinpoint the specific phase of myelin regeneration affected by the treatment.

2. These two models are classified as « severe » animal protocols (significant animal suffering/mortality risk) which is why combining them with busulfan injections (side effects sometimes including mortality) would not be considered ethical. We honestly believe that lysolecithin is an adequate model of demyelination to investigate a pro-remyelinating effect of the procedure, and in terms of this study, provides a proof-of concept that myeloid blood cells could be exploited to reliably deliver pro-regenerative molecules to demyelinating CNS lesions.

2. The authors chose an animal model which remyelinates well and they observed a somewhat accelerated differentiation of oligodendrocytes as well as remyelination. However, especially in chronic MS lesions the tissue environment may inhibit remyelination. There is no animal model that mimics chronically demyelinated MS lesions; however the authors should consider choosing a more "challenging" animal model, e.g. lysolecithin lesions in older animals.

As mentioned above in the response to Reviewer 2, we would like to highlight (it has been reinforced in the current version of the manuscript, particularly in the Discussion) that we hope that treating patients during active disease (relapsing phase, which would be the OPTIMAL phase for treatment) will prevent the chronicity of demyelination by ensuring quick arrival of numerous progenitor cells and remyelination. Thus our strategy should hopefully prevent/reduce chronic demyelination, which is what current immunomodulatory treatments are not achieving. In addition, as specified above, this strategy may be useful to stimulate remyelination in expanding lesions in progressive MS (macrophages on actively demyelinating borders) and/or to enhance neuroprotection by overexpressing neurotrophic factors. We do not think our strategy would be applicable to chronically demyelinated silent lesion core that, in any case, is characterized by extensive axonal loss.

Regarding the comment on age, our animals are approximately 5-6 months old at the time of demyelination, which is older than the usual age used in these experiments (2-3 months) and, in our opinion, provide a proof of concept that genetically-modified macrophages deliver regenerative molecules to the sites of demyelination. While using old animals would probably be a valuable addition to our data, this is technically challenging in terms of chimera generation, for the reasons explained above.

3. My third point is related to this. Remyelination works very well in the typical

demyelinating animal models and is only somewhat slowed down in older animals. However, the situation in MS is more complex. Results from imaging studies suggest that the lesions of many patients display significant remyelination within the first five to six months after lesion formation. So, these patients are most likely not the ones which would benefit from a remyelination promoting therapy. However, there appears to be a subset of lesions which show limited remyelination even in initial lesion stages. More recent studies from initial MS lesions stages suggest that not the lack of mature oligodendrocytes per se contributes to limited remyelination but instead an impaired myelin sheath formation (at least at this lesion stage). At later disease / lesion stages, when remyelination is less frequently observed, the blood brain barrier is relatively closed and it will be questionable whether peripheral cells have a chance to access the CNS parenchyma. Therefore, the authors should discuss more critically which patients at which disease stage and which lesion type might benefit from such a remyelination promoting strategy. They should also discuss critically the limitations of their animal model.

These are all excellent, highly relevant comments that perfectly reflect the difficulties of using animal models to study potential pro-remyelinating strategies in MS.

The large body of literature on neuropathology of early MS demonstrates the existence of several subtypes of demyelinated lesions:

a) those in which mature oligodendrocytes are preserved

b) those in which OPCs but not mature oligodendrocytes are present, and

c) those in which oligodendroglial cells (oligodendrocytes and OPCs) are lacking.

Our strategy would clearly benefit those lesions with complete oligodendroglial depletion (c). However, we think that it would also benefit those lesions in which OPCs are present but do not differentiate (b), or in which they have differentiated but do not remyelinate. It is possible (based on findings in animal models) that, in these lesions, OPCs have arrived to the lesions too late, once the stimulatory conditions for differentiation and remyelination are no longer present (described on the first page of the Discussion). These stimulatory conditions consist of axons that are still permissive for remyelination (as demyelinated axons with time undergo changes, such as the expression of PSA-NCAM, that render them non-permissive for myelination) and presence of acute inflammation that, paradoxically, has been shown to stimulate remyelination, which means that the best timing for remyelination is shortly after demyelination. Thus, improving/accelerating OPC arrival to the lesions during active demyelination using transgene-carrying macrophages, particularly in middle-aged individuals (onset of progression) should bring OPCs to the axons at the right time (quickly) and increase their differentiation and the likelihood of remyelination.

Regarding lesions that contain mature oligodendrocytes, it has been recently shown that OPCs remyelinate more efficiently and accurately than mature oligodendrocytes (Neely et al., 2022; doi: 10.1038/s41593-021-01009-x), which suggests that stimulating OPC-mediated remyelination in these lesions may be useful. In addition, mature oligodendrocytes express netrin-1, a molecule that, as we showed (Tepavcevic et al., 2014) prevents OPC recruitment and reduces remyelination, suggesting that presence of dysfunctional oligodendrocytes in lesions will prevent/reduce OPC recruitment. Thus, overexpressing Sema 3F in these lesions may overcome the inhibitory influence of netrin-1- expressing dysfunctional oligos on OPC recruitment and promote remyelination by newly arrived OPCs. As we and others showed before, the relative

levels of OPC chemoattractants versus OPC chemorepellents are important determinants of OPC numbers in MS lesions.

While these questions and issues have been mentioned in the Discussion, detailed analyses would significantly lengthen the manuscript. We have recently written a review article on OPC recruitment in MS that is currently being evaluated for publication, in which all these issues are addressed in detail.

To summarize, because repeated episodes of demyelination that occur in MS may progressively exhaust the lesional OPC pool, stimulating efficient and fast OPC recruitment should ensure OPC replenishment and quick arrival to demyelinated axons, particularly with age, when OPC recruitment becomes less efficient. Thus, using our Sema 3F strategy, our main goal is to prevent the transition from acute stages to chronically demyelinated stages by stimulating remyelination. We now specify that our target for stimulating OPC recruitment are active lesions (in RRMS) to prevent progression, and expanding lesions (chronic active, progressive MS) to limit the damage (page 35).

With regard to the use of hematopoietic/myeloid gene therapy in chronic progressive disease, we have answered this question to the Reviewer 2 above, the paragraph preceding Reviewer 3 comments:" Regarding progressive disease ……."

We have modified the Discussion to include these comments (pages 35-36), as well as the specification that lysolecithin model is the model of acute demyelination (page 32).

We thank all 3 Reviewers for their insightful comments and suggestions that, we consider, have benefited our manuscript. We hope you will find our revised article suitable for publication in EMBO Molecular Medicine.

1st Revision - Editorial Decision 9th May 2022

9th May 2022

Dear Dr. Tepavcevic,

Thank you for the submission of your revised manuscript to EMBO Molecular Medicine. I am pleased to inform you that we will be able to accept your manuscript pending the following final amendments:

1) Please address all the minor concerns raised by the referees #1 and #2.
2) We note that you currently have together with you, a total of 3 co-corresponding authors. Is that correct? Do you confirm equal contribution of these 3 people, able to take full responsibility for the paper and its content? If so, please provide ORICD IDs for Catherine Lubetzki and Nathalie Cartier. While there is no limit per se to the number of co-corresponding authors, 3 is rare, and may not reflect as intended to the community.
3) In the main manuscript file, please do the following:
- Correct/answer the track changes suggested by our data editors by working from the attached document. Where there is n=2, please show raw values from both measurements and define the nature of the replicates, biological or technical. Please also avoid statistical analyses when n=2. Please refer to the "Author Guidelines" for more information.
https://www.embopress.org/page/journal/17574684/authorguide#statisticalanalysis
- Please shorten the abstract to max. 175 words. Please check "Author Guidelines" for more information.
https://www.embopress.org/page/journal/17574684/authorguide#textformat
- Remove figures from the main manuscript file and leave figure legends at the end of the manuscript.
- Please make sure that all special characters display well.
- Remove "data not shown" (p. 19).
- Move the M&M section after "Discussion".
- We are using the CRediT author contribution format, which allows for systematic, machine readable author contributions and thus more effective research assessment, while also allowing addition of any relevant further information for each author. CRediT replaces the author contribution section. Please use the free text box to provide more detailed descriptions. Please check "Author Guidelines" for more information.
https://www.embopress.org/page/journal/17574684/authorguide#authorshipguidelines
- Merge "Funding" section with "Acknowledgements".
- Raw data from large-scale datasets (RNA sequencing) should be deposited in one of the relevant databases and made freely available prior the publication of the manuscript. Use the following format to report the accession number of your data:

The datasets produced in this study are available in the following databases:
[data type]: [full name of the resource] [accession number/identifier] ([doi or URL or identifiers.org/DATABASE:ACCESSION])

Please check "Author Guidelines" for more information.
https://www.embopress.org/page/journal/17574684/authorguide#availabilityofpublishedmaterial
- Correct the reference citation. In the reference list, citations should be listed in alphabetical order. Where there are more than 10 authors on a paper, 10 will be listed, followed by "et al.". Please check "Author Guidelines" for more information.
https://www.embopress.org/page/journal/17574684/authorguide#referencesformat
4) Conflict of interest: Rename "Conflict of interest" to "Disclosure Statement & Competing Interests". We updated our journal's competing interests policy in January 2022 and request authors to consider both actual and perceived competing interests. Please review the policy https://www.embopress.org/competing-interests and update your competing interests if necessary.
5) Appendix: Please rename figures to Appendix Figure S1 etc. Also, correct appendix figure nomenclature in the main text.
6) The Paper Explained: Please add it to the main manuscript file.
7) Synopsis: Please check your synopsis text and image, revise it if necessary and submit its final versions with your revised manuscript. Please be aware that in the proof stage minor corrections only are allowed (e.g., typos).
8) Funding: INSERM and NeurATRIS is missing in our submission system. Please make sure that information about all sources of funding are complete in both our submission system and in the manuscript.
9) Source data: We encourage you to include the source data for figure panels that show essential data. Numerical data should be provided as individual .xls or .csv files (including a tab describing the data). For blots or microscopy, uncropped images should be submitted (using a zip archive if multiple images need to be supplied for one panel). Please check "Author Guidelines" for more information. https://www.embopress.org/page/journal/17574684/authorguide#sourcedata
10) For more information: There is space at the end of each article to list relevant web links for further consultation by our readers. Could you identify some relevant ones and provide such information as well? Some examples are patient associations, relevant databases, OMIM/proteins/genes links, author's websites, etc...
11) As part of the EMBO Publications transparent editorial process initiative (see our Editorial at http://embomolmed.embopress.org/content/2/9/329), EMBO Molecular Medicine will publish online a Review Process File (RPF) to accompany accepted manuscripts. This file will be published in conjunction with your paper and will include the anonymous referee reports, your point-by-point response and all pertinent correspondence relating to the manuscript. Let us know whether you agree with the publication of the RPF and as here, if you want to remove or not any figures from it prior to publication. Please note that the Authors checklist will be published at the end of the RPF.

12) Please provide a point-by-point letter INCLUDING my comments as well as the reviewer's reports and your detailed responses (as Word file).

I look forward to reading a new revised version of your manuscript as soon as possible.

***** Reviewer's comments *****

Referee #1 (Comments on Novelty/Model System for Author):

rather novel approach to enhance repair in the CNS but still early to predict if it will translate

Referee #1 (Remarks for Author):

The authors provide a well thought out response to the issues raised dealing with both conceptual and experimental issues raised.

An interesting and challenging conceptual issue as raised by all reviewers is how will one translate the current approach of delivering a gene repair-promoting product in the actual setting of MS. This reviewer found to concept of having a source of such molecules persistent in the CNS so as to be ready to be delivered when a focal demyelinating lesion develops to be particular

interest. This was more clearly stated in the revision.
One would like some expansion of this concept by adding some further comments.
-How long would the trasnsfected macrophages persist in the CNS either if they access the normal CNs or after a demyelinating lesion. Data from Fig 5 (day 60) would suggest that survival would be limited - is this correct?
- The other suggested experimental models especially EAE would be of interest ie would macrophages recruited as part of an ongoing inflammatory response persist indefinitely and be ready for another demyelinating episode?
-Would macrophages access an area with an established glial scar?

The authors respond clearly to the issue of why the lysolecithin model was chosen,
They also provide requested comparative data on the robustness of the Sema delivery by macrophages effect with other delivery systems and other myelination enhancers. One hopes the effects will be sufficient translate into the clinics.

One appreciates the extensive work done and the technical problems encountered (vacuolation toxicity) in trying to analyze later time point in terms of myelination. One accepts this will not be part of the current manuscript.

Referee #2 (Remarks for Author):

This manuscript has been improved by responses to the comments of all three reviewers. I only have three minor suggestions now to further improve the paper

First, the authors should provide more detail on how blinding was achieved in the analysis of remyelination. At the moment they simply say that analysis was performed "blindly". I suspect that those performing future meta-analyses of the remyelination literature will value greater detail in those papers to which they are going to ascribe the greatest weight. So, I suggest an explicit statement as to how the analysis was performed in such a way that the observer/counter was completely unaware of the experimental condition being analysed

Second, I think an opportunity is being missed by not performing a 3-D reconstruction of the relationship between the transduced macrophages and OPCs within the lesions. This wouldn't require electron microscopy, so that the transduced and normal macrophages could be distinguished. Such an image would provide a powerful visual summary of the potential impact and importance of the work

Third, the new figure S3 showing no difference in myelin debris ingestion by the macrophages is very helpful and should be included in the main figures

Referee #3 (Remarks for Author):

The authors have addressed all my comments.

***** Reviewer's comments *****

Referee #1 (Comments on Novelty/Model System for Author):

rather novel approach to enhance repair in the CNS but still early to predict if it will translate

Referee #1 (Remarks for Author):

The authors provide a well thought out response to the issues raised dealing with both conceptual and experimental issues raised. We thank the reviewer for this evaluation.

An interesting and challenging conceptual issue as raised by all reviewers is how will one translate the current approach of delivering a gene repair-promoting product in the actual setting of MS. This reviewer found to concept of having a source of such molecules persistent in the CNS so as to be ready to be delivered when a focal demyelinating lesion develops to be particular interest. This was more clearly stated in the revision.
One would like some expansion of this concept by adding some further comments.
-How long would the trasnsfected macrophages persist in the CNS either if they access the normal CNs or after a demyelinating lesion. Data from Fig 5 (day 60) would suggest that survival would be limited - is this correct?

Our data suggest that transgene-carrying macrophages progressively leave demyelinating lesions, even though, as indicated in the manuscript, we did observe persistence of some GFP+ cells both in lesions and non-lesioned tissue at 60 dpl. We do not have enough data to deduce whether these cells die or leave the CNS. We have included a paragraph with these reflections on page 17 in the Discussion section.

However, it is interesting to mention that busulfan, the pre-conditioning treatment, enhances CNS engraftment of bone-marrow derived cells, as mentioned in the manuscript, which could explain the persistence of transgene-carrying cells at late time points.
- The other suggested experimental models especially EAE would be of interest ie would macrophages recruited as part of an ongoing inflammatory response persist indefinitely and be ready for another demyelinating episode?

Previous studies, as referenced in the manuscript, suggest that blood- derived cells can establish within the CNS following busulfan treatment. It is thus possible that these cells could react to another demyelinating episode, which would be of particular interest for progressive MS, when immune infiltration from the blood into the brain is reduced. Otherwise, in active MS, transgene-carrying cells present in the blood during this consecutive episode could also infiltrate the tissue. In the previous revision, we addressed these issues on page 19, and have now added additional comments (in red) for further clarifications.
-Would macrophages access an area with an established glial scar?
This is a very important point. It is thought that macrophages are recruited to the areas of demyelination early, prior to the formation of glial scar, so we hope that Sema3F-carrying macrophages will enable us to stimulate OPC recruitment prior to glial scar formation. Lesions with an established astrocytic scar are usually chronic lesions with very little, if any, inflammatory demyelinating activity and extensive axonal loss, which as we indicated in the Discussion, are not suitable targets for our strategy. We have added a sentence to clarify this issue, on page 19: *"In this manner, OPC recruitment would be stimulated prior to the formation of glial scar, to promote remyelination thus preventing/reducing axonal loss."*

What we hope to achieve, as mentioned in the Discussion, is to prevent the formation of chronic lesions by stimulating early OPC recruitment during demyelinating phase.
The authors respond clearly to the issue of why the lysolecithin model was chosen, They also provide requested comparative data on the robustness of the Sema delivery by macrophages effect with other delivery systems and other myelination enhancers.

One hopes the effects will be sufficient translate into the clinics.

One appreciates the extensive work done and the technical problems encountered (vacuolation toxicity) in trying to analyze later time point in terms of myelination. One accepts this will not be part of the current manuscript. We sincerely thank the reviewer for this comment.

Referee #2 (Remarks for Author):

This manuscript has been improved by responses to the comments of all three reviewers. We thank the Reviewer for this evaluation.

I only have three minor suggestions now to further improve the paper

First, the authors should provide more detail on how blinding was achieved in the analysis of remyelination. At the moment they simply say that analysis was performed "blindly". I suspect that those performing future meta-analyses of the remyelination literature will value greater detail in those papers to which they are going to ascribe the greatest weight. So, I suggest an explicit statement as to how the analysis was performed in such a way that the observer/counter was completely unaware of the experimental condition being analysed We are now providing this information in detail in Methods section, on page 29. For EM: "*Ultrathin sections were cut by one investigator and imaged and quantified by different investigators. The investigator who cut the ultrathin sections placed the grids in the grid box in random order. The investigator in charge of imaging acquired the images and named them using the grid-box well identifier. After quantification, the identity of the grid was revealed."* For IHC (page 28) *"The investigator in charge of imaging acquired the images and named them using the mouse unique identificator (without age or condition). Quantification was performed blinded, the identity/age/condition of each mouse was revealed after quantification*."

Second, I think an opportunity is being missed by not performing a 3-D reconstruction of the relationship between the transduced macrophages and OPCs within the lesions. This wouldn't require electron microscopy, so that the transduced and normal macrophages could be distinguished. Such an image would provide a powerful visual summary of the potential impact and importance of the work

We are grateful to the Reviewer for inciting us to perform these analyses. We find this representation more powerful than the images we provided previously. Thus, the 3D representations of Olig2 (OPC) and GFP co-labelings of the lesions shown in the Figure 6E-F are now provided as panels 6E'-F'. We have also included the 3D reconstruction of Sema3F-overexpressing lesion in our graphical abstract.

Third, the new figure S3 showing no difference in myelin debris ingestion by the macrophages is very helpful and should be included in the main figures

We thank the Reviewer for this appreciation. However, the figure that would most suitably accommodate the Oil Red O data would be the Figure 5 (immune status of Sema3F lesions), but this figure has so many panels already that we even had to add non-English letters Ç and Ñ. We feel that Oil Red O data on their own are not sufficient for a separate main figure, so if it is OK with the Reviewer and the Editors, we would prefer to keep these data in a Supplementary Figure, or if Editors find it more suitable, we can include it as Extended Figure 1.

Referee #3 (Remarks for Author):

The authors have addressed all my comments. We thank the Reviewer for this evaluation.

2nd Jun 2022

Dear Dr. Tepavcevic,

We are pleased to inform you that your manuscript is accepted for publication and is now being sent to our publisher to be included in the next available issue of EMBO Molecular Medicine.

Please read below for additional IMPORTANT information regarding your article, its publication and the production process.

Congratulations on your interesting work,

Zeljko Durdevic

EMBO Press Author Checklist

Corresponding Author Name: Vanja Tepavcevic	
Journal Submitted to: EMBO Molecular Medicine	
Manuscript Number: EMM-2021-14759v3	

USEFUL LINKS FOR COMPLETING THIS FORM
[The EMBO Journal - Author Guidelines]
[EMBO Reports - Author Guidelines]
[Molecular Systems Biology - Author Guidelines]
[EMBO Molecular Medicine - Author Guidelines]

Reporting Checklist for Life Science Articles (updated January 2022)
This checklist is adapted from Materials Design Analysis Reporting (MDAR) Checklist for Authors. MDAR establishes a minimum set of requirements in transparent reporting in the life sciences (see Statement of Task: 10.31222/osf.io/9sm4x). Please follow the journal's guidelines in preparing your manuscript.
Please note that a copy of this checklist will be published alongside your article.

Abridged guidelines for figures
1. Data
The data shown in figures should satisfy the following conditions:
- the data were obtained and processed according to the field's best practice and are presented to reflect the results of the experiments in an accurate and unbiased manner.
- ideally, figure panels should include only measurements that are directly comparable to each other and obtained with the same assay.
- plots include clearly labeled error bars for independent experiments and sample sizes. Unless justified, error bars should not be shown for technical replicates.
- if n<5, the individual data points from each experiment should be plotted. Any statistical test employed should be justified.
- Source Data should be included to report the data underlying figures according to the guidelines set out in the authorship guidelines on Data Presentation.

2. Captions
Each figure caption should contain the following information, for each panel where they are relevant:
- a specification of the experimental system investigated (eg cell line, species name).
- the assay(s) and method(s) used to carry out the reported observations and measurements.
- an explicit mention of the biological and chemical entity(ies) that are being measured.
- an explicit mention of the biological and chemical entity(ies) that are altered/varied/perturbed in a controlled manner.
- the exact sample size (n) for each experimental group/condition, given as a number, not a range;
- a description of the sample collection allowing the reader to understand whether the samples represent technical or biological replicates (including how many animals, litters, cultures, etc.).
- a statement of how many times the experiment shown was independently replicated in the laboratory.
- definitions of statistical methods and measures:
  - common tests, such as t-test (please specify whether paired vs. unpaired), simple χ2 tests, Wilcoxon and Mann-Whitney tests, can be unambiguously identified by name only, but more complex techniques should be described in the methods section;
  - are tests one-sided or two-sided?
  - are there adjustments for multiple comparisons?
  - exact statistical test results, e.g., P values = x but not P values < x;
  - definition of 'center values' as median or average;
  - definition of error bars as s.d. or s.e.m.

Please complete ALL of the questions below.
Select "Not Applicable" only when the requested information is not relevant for your study.

Materials

Newly Created Materials	Information included in the manuscript?	In which section is the information available? (Reagents and Tools Table, Materials and Methods, Figures, Data Availability Section)
New materials and reagents need to be available; do any restrictions apply?	Not Applicable	

Antibodies	Information included in the manuscript?	In which section is the information available? (Reagents and Tools Table, Materials and Methods, Figures, Data Availability Section)
For **antibodies** provide the following information:<br>- Commercial antibodies: RRID (if possible) or supplier name, catalogue number and or/clone number<br>- Non-commercial: RRID or citation	Yes	Materials and Methods

DNA and RNA sequences	Information included in the manuscript?	In which section is the information available? (Reagents and Tools Table, Materials and Methods, Figures, Data Availability Section)
**Short novel DNA or RNA including primers, probes**: provide the sequences.	Not Applicable	

Cell materials	Information included in the manuscript?	In which section is the information available? (Reagents and Tools Table, Materials and Methods, Figures, Data Availability Section)
**Cell lines:** Provide species information, strain. Provide accession number in repository **OR** supplier name, catalog number, clone number, and/**OR** RRID.	Yes	Materials and methods
**Primary cultures:** Provide species, strain, sex of origin, genetic modification status.	Yes	Materials and methods
Report if the cell lines were recently **authenticated** (e.g., by STR profiling) and tested for mycoplasma contamination.	Not Applicable	

Experimental animals	Information included in the manuscript?	In which section is the information available? (Reagents and Tools Table, Materials and Methods, Figures, Data Availability Section)
**Laboratory animals or Model organisms:** Provide species, strain, sex, age, genetic modification status. Provide accession number in repository **OR** supplier name, catalog number, clone number, **OR** RRID.	Yes	Materials and methods
**Animal observed in or captured from the field:** Provide species, sex, and age where possible.	Not Applicable	
Please detail **housing and husbandry conditions**.	Yes	Materials and methods

Plants and microbes	Information included in the manuscript?	In which section is the information available? (Reagents and Tools Table, Materials and Methods, Figures, Data Availability Section)
**Plants:** provide species and strain, ecotype and cultivar where relevant, unique accession number if available, and source (including location for collected wild specimens).	Not Applicable	
**Microbes:** provide species and strain, unique accession number if available, and source.	Not Applicable	

Human research participants	Information included in the manuscript?	In which section is the information available? (Reagents and Tools Table, Materials and Methods, Figures, Data Availability Section)
If collected and within the bounds of privacy constraints report on age, sex and gender or ethnicity for all study participants.	Not Applicable	

Core facilities	Information included in the manuscript?	In which section is the information available? (Reagents and Tools Table, Materials and Methods, Figures, Data Availability Section)
If your work benefited from core facilities, was their service mentioned in the acknowledgments section?	Yes	Materials and methods, Acknowledgements

Design

Study protocol	Information included in the manuscript?	In which section is the information available? (Reagents and Tools Table, Materials and Methods, Figures, Data Availability Section)
If study protocol has been **pre-registered, provide DOI in the manuscript**. For clinical trials, provide the trial registration number **OR** cite DOI.	Not Applicable	
Report the **clinical trial registration number** (at ClinicalTrials.gov or equivalent), where applicable.	Not Applicable	

Laboratory protocol	Information included in the manuscript?	In which section is the information available? (Reagents and Tools Table, Materials and Methods, Figures, Data Availability Section)
Provide DOI OR other citation details if **external detailed step-by-step protocols** are available.	Not Applicable	

Experimental study design and statistics	Information included in the manuscript?	In which section is the information available? (Reagents and Tools Table, Materials and Methods, Figures, Data Availability Section)

Include a statement about **sample size** estimate even if no statistical methods were used.	Yes	Results, Figure Legends
Were any steps taken to minimize the effects of subjective bias when allocating animals/samples to treatment (e.g. **randomization procedure**)? If yes, have they been described?	Yes	Materials and Methods
Include a statement about **blinding** even if no blinding was done.	Yes	Materials and Methods
Describe **inclusion/exclusion criteria** if samples or animals were excluded from the analysis. Were the criteria pre-established?<br><br>If sample or data points were omitted from analysis, report if this was due to attrition or intentional exclusion and provide justification.	Not Applicable	
For every figure, are **statistical tests** justified as appropriate? Do the data meet the assumptions of the tests (e.g., normal distribution)? Describe any methods used to assess it. Is there an estimate of variation within each group of data? Is the variance similar between the groups that are being statistically compared?	Yes	Materials and Methods-Statistical Analyses

Sample definition and in-laboratory replication	Information included in the manuscript?	In which section is the information available?<br>(Reagents and Tools Table, Materials and Methods, Figures, Data Availability Section)
In the figure legends: state number of times the experiment was **replicated** in laboratory.	Yes	Figure legends
In the figure legends: define whether data describe **technical or biological replicates**.	Yes	Figurelegends

Ethics

Ethics	Information included in the manuscript?	In which section is the information available?<br>(Reagents and Tools Table, Materials and Methods, Figures, Data Availability Section)
Studies involving **human participants**: State details of **authority granting ethics approval** (IRB or equivalent committee(s), provide reference number for approval.	Not Applicable	
Studies involving **human participants**: Include a statement confirming that **informed consent** was obtained from all subjects and that the experiments conformed to the principles set out in the WMA Declaration of Helsinki and the Department of Health and Human Services Belmont Report.	Not Applicable	
Studies involving **human participants:** For publication of **patient photos**, include a statement confirming that consent to publish was obtained.	Not Applicable	
Studies involving experimental **animals**: State details of **authority granting ethics approval** (IRB or equivalent committee(s), provide reference number for approval. Include a statement of compliance with ethical regulations.	Yes	Materials and Methods
Studies involving **specimen and field samples:** State if relevant **permits** obtained, provide details of authority approving study; if none were required, explain why.	Not Applicable	

Dual Use Research of Concern (DURC)	Information included in the manuscript?	In which section is the information available?<br>(Reagents and Tools Table, Materials and Methods, Figures, Data Availability Section)
Could your study fall under dual use research restrictions? Please check biosecurity documents and list of **select agents and toxins** (CDC): https://www.selectagents.gov/sat/list.htm	Not Applicable	
If you used a select agent, is the security level of the lab appropriate and reported in the manuscript?	Not Applicable	
If a study is subject to dual use research of concern regulations, is the name of the **authority granting approval and reference number** for the regulatory approval provided in the manuscript?	Not Applicable	

Reporting

The MDAR framework recommends adoption of discipline-specific guidelines, established and endorsed through community initiatives. Journals have their own policy about requiring specific guidelines and recommendations to complement MDAR.

Adherence to community standards	Information included in the manuscript?	In which section is the information available?<br>(Reagents and Tools Table, Materials and Methods, Figures, Data Availability Section)
State if relevant guidelines or checklists (e.g., **ICMJE, MIBBI, ARRIVE, PRISMA**) have been followed or provided.	Yes	Material and Methods
For **tumor marker prognostic studies,** we recommend that you follow the **REMARK** reporting guidelines (see link list at top right). See author guidelines, under 'Reporting Guidelines'. Please confirm you have followed these guidelines.	Not Applicable	
For **phase II and III randomized controlled trials**, please refer to the **CONSORT** flow diagram (see link list at top right) and submit the CONSORT checklist (see link list at top right) with your submission. See author guidelines, under 'Reporting Guidelines'. Please confirm you have submitted this list.	Not Applicable	

Data Availability

Data availability	Information included in the manuscript?	In which section is the information available?<br>(Reagents and Tools Table, Materials and Methods, Figures, Data Availability Section)
Have **primary datasets** been deposited according to the journal's guidelines (see 'Data Deposition' section) and the respective accession numbers provided in the Data Availability Section?	Not Applicable	
Were **human clinical and genomic datasets** deposited in a public access-controlled repository in accordance to ethical obligations to the patients and to the applicable consent agreement?	Not Applicable	
Are **computational models** that are central and integral to a study available without restrictions in a machine-readable form? Were the relevant accession numbers or links provided?	Not Applicable	
If publicly available data were reused, provide the respective **data citations in the reference list**.	Yes	Materials and Methods, Data availability, References
